# A genome-wide association study of social trust in 33,882 Danish blood donors

**DOI:** 10.1038/s41598-024-51636-0

**Published:** 2024-01-16

**Authors:** Celia Burgos Sequeros, Thomas Folkmann Hansen, David Westergaard, Ioannis Louloudis, Sebastian Kalamajski, Timo Röder, Palle Duun Rohde, Michael Schwinn, Line Harder Clemmensen, Maria Didriksen, Mette Nyegaard, Henrik Hjalgrim, Kaspar René Nielsen, Mie Topholm Bruun, Sisse Rye Ostrowski, Christian Erikstrup, Susan Mikkelsen, Erik Sørensen, DBDS Genomic Consortium, Karina Banasik, Jakob Bay, Jens Kjærgaard Boldsen, Thorsten Brodersen, Søren Brunak, Kristoffer Burgdorf, Mona Ameri Chalmer, Maria Didriksen, Khoa Manh Dinh, Joseph Dowsett, Christian Erikstrup, Bjarke Feenstra, Frank Geller, Daniel Gudbjartsson, Thomas Folkmann Hansen, Lotte Hindhede, Henrik Hjalgrim, Rikke Louise Jacobsen, Gregor Jemec, Bitten Aagaard Jensen, Katrine Kaspersen, Bertram Dalskov Kjerulff, Lisette Kogelman, Margit Anita Hørup Larsen, Ioannis Louloudis, Agnete Lundgaard, Susan Mikkelsen, Christina Mikkelsen, Ioanna Nissen, Mette Nyegaard, Sisse Rye Ostrowski, Ole Birger Vestager Pedersen, Alexander Pil Henriksen, Palle Duun Rohde, Klaus Rostgaard, Michael Schwinn, Kari Stefansson, Hreinn Stefánsson, Erik Sørensen, Unnur Thorsteinsdóttir, Lise Wegner Thørner, Mie Topholm Bruun, Henrik Ullum, Thomas Werge, David Westergaard, Ole Birger Vestager Pedersen, Søren Brunak, Karina Banasik, Giuseppe Nicola Giordano

**Affiliations:** 1 Translational Disease Systems Biology, Novo Nordisk Foundation Center for Protein Research, Faculty of Health and Medical Sciences, University of Copenhagen, Copenhagen, Denmark; 2 Department of Neurology, Danish Headache Center, Copenhagen University Hospital - Rigshospitalet, Glostrup, Denmark; 3 Methods and Analysis, Statistics Denmark, Copenhagen, Denmark; 4 Department of Gynecology and Obstetrics, Copenhagen University Hospital Hvidovre, Copenhagen, Denmark; 5 Genetic and Molecular Epidemiology Unit, Department of Clinical Sciences, CRC, Lund University Diabetes Centre, Malmö, Sweden; 6 Genomic Medicine, Department of Health Science and Technology, Aalborg University, Gistrup, Denmark; 7 Department of Clinical Immunology, Copenhagen University Hospital - Rigshospitalet, Copenhagen, Denmark; 8 Department of Applied Mathematics and Computer Science, Technical University of Denmark, Kongens Lyngby, Denmark; 9 The Danish Cancer Institute, Copenhagen, Denmark; 10 Department of Epidemiology Research, Statens Serum Institut, Copenhagen, Denmark; 11 Department of Haematology, Rigshospitalet, Copenhagen, Denmark; 12 Department of Clinical Medicine, Faculty of Health and Medical Sciences, University of Copenhagen, Copenhagen, Denmark; 13 Department of Clinical Immunology, Zealand University Hospital, Køge, Denmark; 14 Clinical Immunology Research Unit, Department of Clinical Immunology, Odense University Hospital, Odense, Denmark; 15 Department of Clinical Immunology, Aarhus University Hospital, Aarhus, Denmark; 16 Department of Clinical Medicine, Aarhus University, Aarhus, Denmark; 17 deCODE Genetics, Reykjavík, Iceland; 18 Department of Dermatology, Zealand University Hospital, Roskilde, Denmark; 19 Department of Clinical Immunology, Aalborg University Hospital, Aalborg, Denmark; 20 Statens Serum Institut, Copenhagen, Denmark; 21 Institute of Biological Psychiatry, Mental Health Centre, Sct. Hans, Copenhagen University Hospital, Roskilde, Denmark

**Keywords:** Behavioural genetics, Genome-wide association studies

## Abstract

Social trust is a heritable trait that has been linked with physical health and longevity. In this study, we performed genome-wide association studies of self-reported social trust in n = 33,882 Danish blood donors. We observed genome-wide and local evidence of genetic similarity with other brain-related phenotypes and estimated the single nucleotide polymorphism-based heritability of trust to be 6% (95% confidence interval = (2.1, 9.9)). In our discovery cohort (n = 25,819), we identified one significantly associated locus (lead variant: rs12776883) in an intronic enhancer region of *PLPP4*, a gene highly expressed in brain, kidneys, and testes. However, we could not replicate the signal in an independent set of donors who were phenotyped a year later (n = 8063). In the subsequent meta-analysis, we found a second significantly associated variant (rs71543507) in an intergenic enhancer region. Overall, our work confirms that social trust is heritable, and provides an initial look into the genetic factors that influence it.

## Introduction

Social trust is an abstract attitude toward other people, especially strangers, which has been shown to independently predict good health and longevity^1–3^. Most noticeably, individuals who trust strangers seem to have a reduced risk of major adverse cardiovascular events (MACE) compared with individuals who do not^4^. According to the psychosocial hypothesis, trust could mitigate uncertainty and stress in unfamiliar interactions^5^, while distrust could heighten perceived stressors and lead to an overstimulation of the hypothalamic–pituitary–adrenal (HPA) axis during social encounters^6^. Long-term, this could result in chronically elevated blood cortisol, increased platelet cohesion, and arterial plaque formation, all of which increase the risk of MACE, and thus, earlier death^7^. The link between mental and cardiovascular health is not unique to social trust; it has also been recognized in large epidemiological studies on depression, chronic psychological stress, post-traumatic stress disorder, and anxiety^8–11^.

Social trust can be conceptualized as an intrinsic quality of the individual, a collective state facilitated by a safe, organized and honest society, or a combination of the two^12^. How it is measured varies accordingly^13^. At the individual level, it is unclear what builds trust. Some suggest that it is the outcome of cumulative experiences, both positive and negative^14,15^; others propose that it is a moral resource that is established early in life and remains stable over time^16,17^. Longitudinal data reveal that trust can be perturbed by stressful events, but often returns to baseline levels after the stress has passed^18^.

Social trust has been shown to be heritable. Twin studies on the more widely investigated Big Five personality domains^19^ (extraversion, agreeableness, openness, conscientiousness, and neuroticism), partition genetic effects, shared environmental (familial) and non-shared (unique) experiences, and estimate that around 40% of the variance in these traits are explained by genetic effects^20,21^. The heritability of social trust specifically has been evaluated in Australian^22^, Swedish^23^, English^24^ and Dutch^25^ twins, and estimates range broadly from 5 to 50%, likely owing to the definition of the trust phenotype, its underlying complex genetics, the assumptions of twin study designs, and the diversity of study populations. A single genome-wide association study (GWAS) of social trust has been performed before by Wootton et al.^24^, but the sample size of n < 1700 was underpowered to detect any significant associations with genetic sequence variants.

Several studies have also explored candidate genes that, based on their previously observed function, are likely to play a part in pro-social and trust behaviors. Albeit inconsistent, results have drawn attention to the genes encoding the serotonin transporter (*SLC6A4*)^26,27^, the oxytocin receptor (*OXTR*)^24,28–30^, *CD38*^29,31^*,* and the arginine vasopressin receptor 1A (*AVPR1A*)^32^.

Thus, we build on previous evidence that genetics influence social trust by performing the largest GWAS to date on a self-reported measure of this phenotype among 33,882 individuals from the Danish Blood Donor Study (DBDS)^33,34^. By identifying associated sequence variants, we aim to begin revealing the biological mechanisms underlying social trust, which may later be of use in validating its observed connection with physical health.

## Results

### Characteristics of the discovery cohort

**Figure 1:**
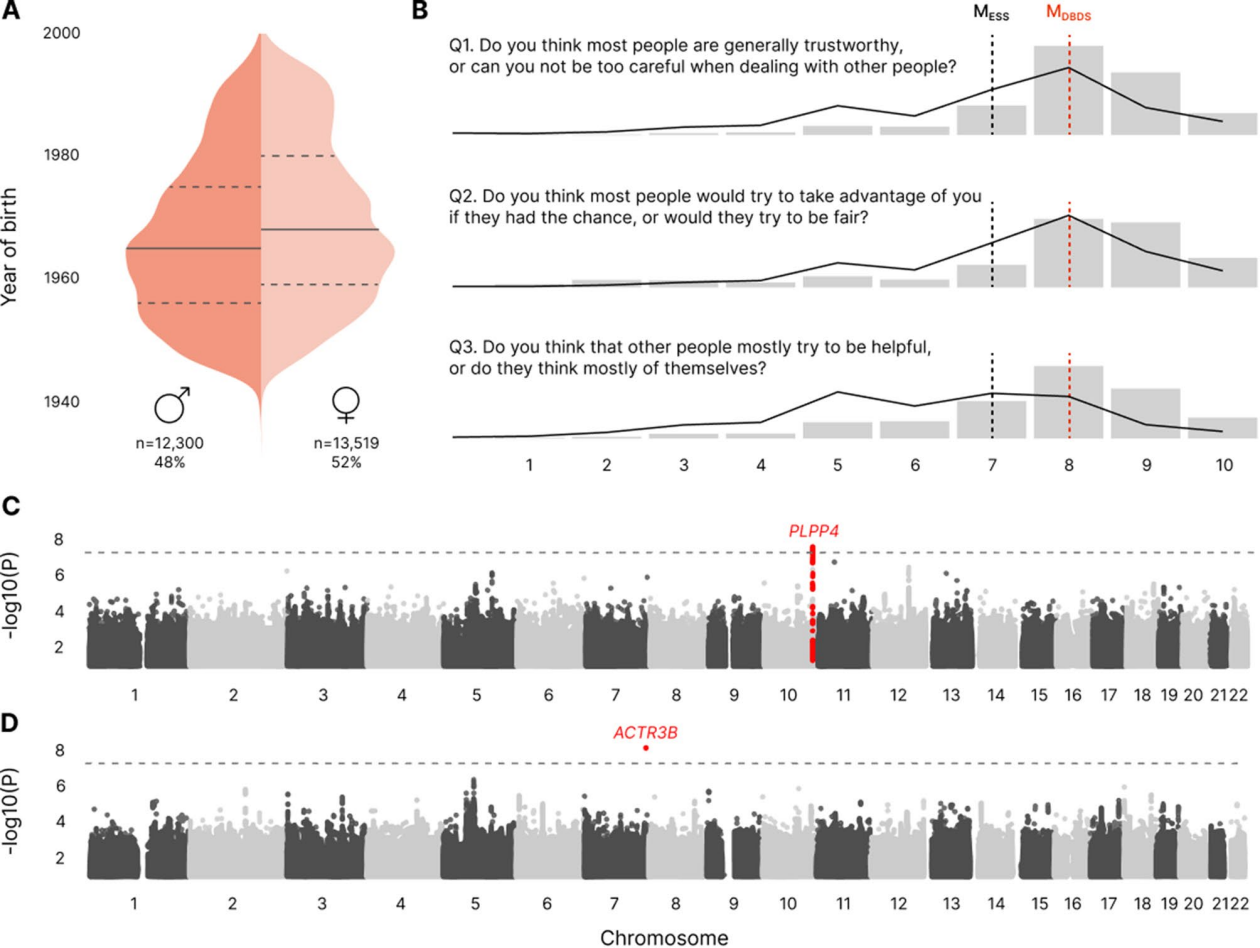
Social trust in the DBDS. (**A**) Sex and year of birth distributions in the discovery cohort. Solid lines represent the median year of birth (1968 and 1965 for men and women, respectively); dashed lines represent the 25th and 75th quartiles. (**B**) The three social trust questionnaire items and their responses in the DBDS discovery cohort (gray bars; n = 25,819; median in red) and a random sample of the Danish population (black line; n = 10,369; source: European Social Survey; median in black). (**C**) Manhattan plot of associations between genetic sequence variants and social trust in the discovery cohort. (**D**) Manhattan plot of associations between genetic sequence variants and social trust in the meta-analysis. In (**C**, **D**), the horizontal dotted line represents the Bonferroni-corrected 5e−08 significance threshold. The genomic loci found to be associated with social trust (see Table 1) are colored in red and the closest protein-coding gene is indicated.

The discovery cohort comprised 25,819 participants from the DBDS, for which the level of self-reported social trust was collected using an electronic questionnaire administered in May 2021. The cohort included 52% women and the median age was 56 years (quartiles: 45–65) (Fig. 1A).

### Social trust in the DBDS

We used three validated questionnaire items from the European Social Survey to capture the level of social trust in our cohort. These questions were originally designed to measure trust as an individual-level attitude^35^, but arguably also partly reflect the societal context in which they are asked. Figure 1B holds the three items and the distributions of their responses, on a scale from one to ten, in the discovery cohort. Principal component (PC) analysis found that the first PC captured 66.8% of the variance and was positively correlated with all three items, while the second and third PCs gathered the remaining inter-item differences (Supplementary Fig. 5). In order to retain the full multidimensionality and later facilitate replicability, the overall level of social trust was determined by calculating the average score of the three items. The cohort showed a median social trust score of eight (quartiles: seven-nine), while the general Danish population consistently reports a median score of seven (quartiles: six-eight) when measured with the same three items through the European Social Survey (Fig. 1B, Supplementary Table 1).

### Genome-wide association study (GWAS) of social trust in the discovery cohort

We tested > 17 million common single-nucleotide polymorphisms (SNPs) and found one locus associated with social trust in the discovery cohort (Fig. 1C, Table 1). This locus contained six intronic sequence variants within the Phospholipid Phosphatase 4 gene (*PLPP4,* chr10:122,216,466–122,351,577 in the hg19 genome build), all genome-wide significant (lowest *p* value = 2.4e−08). Variants displayed comparable effects on the phenotype (Supplementary Fig. 6), and similar minor allele frequencies that were consistent with that of other non-Finnish North-western European populations in gnomAD^36^ and of a Danish genome reference panel (DanMAC5^37^; Supplementary Table 2). All were in close linkage disequilibrium (LD, r^2^ > 0.89) with the lead variant (rs12776883), which we focused the subsequent analyses on. The SNP-based heritability of social trust in the discovery cohort amounted to h^2^_SNP_ = 5.4% (95% confidence interval, CI = (1.5, 9.3)).

**Table 1:** Sequence variants significantly associated with social trust in the discovery cohort or the meta-analysis.

Lead sequence variant	Genomic position (hg19)	Alt/ref alleles	MAF	Genes	Other genome-wide significant variants tested in the locus (LD with lead SNP r^2^ > 0.89)	Study	Beta	*p* value
rs12776883	chr10:122340062	T/C	0.27	*PLPP4, LINC01561, LINC02930*	rs17633662, rs76443833, rs17634034, rs11597267, rs34954484	Discovery	− 0.1	2.4e−08
						Internal replication	0.028	0.363
						Meta-analysis	− 0.07	1.76e−05
rs71543507	chr7:152619890	G/A	0.09	*ACTR3B, ENSG00000286565*	–	Discovery	0.14	1.17e−06
						Internal replication	0.163	1.11e−03
						Meta-analysis	0.15	6.9e−09

Genes shown are those either linked through eQTL activity to the lead variant or physically closest to it. In the case of rs71543507, we report the two closest genes, as the first is a very poorly characterized gene not available in the NCBI RefSeq dataset.

*alt/ref alleles* Alternative/reference alleles, *MAF* Minor allele frequency, *LD* Linkage disequilibrium.

### Internal replication

For internal replication of our findings, we used a smaller, independent sample of 8063 DBDS participants who completed the same questionnaire one year later (July 2022). The replication cohort displayed similar demographic characteristics as the discovery cohort (Supplementary Fig. 2) but was analyzed separately due to the considerable difference in the country’s social situation at the two time points (May 2021 coincided with the end of the second COVID-19 lockdown in Denmark, while July 2022 marked a year since the full removal of sanitary restrictions). We did not observe any sequence variants associated with social trust in the replication cohort and were unable to replicate the *PLPP4* association signal of the discovery cohort (Supplementary Figs. 8 and 10). The genetic correlation between the two studies was not significantly different from zero (r_g_ = 1.6, 95% CI = (− 3.7, 6.9), *p* value = 0.55) and out of bounds likely due to the combined low heritability (see Methods).

### Meta-analysis

We then performed a meta-analysis of the results of the discovery and internal replication GWAS. We found a single genome-wide significant sequence variant (rs71543507, Fig. 1D, Table 1) in an intergenic region of chromosome 7, 2.4kB upstream of a long intergenic non-protein coding RNA gene (*ENSG00000286565*, chr7:152,622,318–152,634,066 in hg19), and 67kB downstream of the actin related protein 3B-coding gene (*ACTR3B,* chr7:152,456,837–152,552,463 in hg19). *ENSG00000286565* was not available in the NCBI RefSeq^38^ database as of writing, due to its novelty and poor characterization. There was no support for the signal from other variants in the area; the LD of the ten surrounding variants were r^2^ < 0.4 (Supplementary Fig. 9). The observed MAF of rs71543507 (Table 1) was consistent with that of other non-Finnish North-western European populations in gnomAD and of DanMAC5 (Supplementary Table 2). The *PLPP4* signal did not pass the genome-wide significance threshold (Supplementary Fig. 10). Based on the results of the meta-analysis, the SNP-based heritability estimate of social trust increased to h^2^_SNP_ = 6% (95% CI = (2.1, 9.9)).

### External replication

To the best of our knowledge, social trust has only been measured once before in a genomic cohort (n < 1700) by Wootton et al.^24^. This study measured trust with a single item (“In general, I think people can be trusted”) and responses were given on a dichotomous scale, either yes or no. We compared the findings of our meta-analysis with the top 50 signals available from this study and found inconsistent concordance in effect sizes and *p* values (Supplementary Table 7). For example, of the 22 reported sequence variants (20 present in our dataset) in the contactin-associated protein 2 gene (*CNTNAP2*), previously implicated in multiple neurodevelopmental disorders^38^, only nine showed the same direction of effect, three of which had a *p* value < 0.05 in our meta-analysis. Similarly, of the 17 variants (16 in our dataset) mapping to the GC-Rich Sequence DNA-Binding Factor 2 gene (*GCFC2*), previously associated with dyslexia; and the Leucine-Rich Repeat Transmembrane Neuronal Protein 4 gene (*LRRTM4*), linked with schizophrenia and epilepsy, eight agreed in the direction of effect, but none had a *p* value < 0.05 in our meta-analysis. Our lead sequence variants were not among the top 50 signals in the study by Wootton et al.

### The biology of variants associated with social trust

Functional annotation of the lead sequence variants was performed to investigate their potential biological effects (Table 1). According to RegulomeDB^39^ ranks, both rs12776883 and rs71543507 are likely to affect transcription factor binding (1f and 2b, respectively), and score low in the combined annotation-dependent depletion (CADD) scale^40^ of deleteriousness (0.8 and 2.3, respectively). ChromHMM^41^ predicts rs12776883 to be part of an active enhancer in brain, eye, kidney and liver. Transposase-accessible chromatin (ATAC) and chromatin immunoprecipitation (ChIP) sequencing experiments summarized in the RegulomeDB resource indicate that this variant is most commonly found as open chromatin in motor neuron biosamples, and interacts with four transcription factors in neural cells (REST, POLR, TAF1, EP300), one in kidneys (ZSCAN4), and one in blood (RFX1). On the other hand , rs71543507 could be part of an active enhancer in brain, liver, mammary glands and prostate gland, displaying an open chromatin configuration most often in the adrenal glands, and interacting with six transcription factors in the brain (EP300, RCOR1, CHD2, TFAP2B, GATA2, TFC4), and one in the bone marrow (GATA2). eQTL data from the Genotype-Tissue Expression (GTEx) v8 resource^42,43^ reveal an association between rs12776883 and the expression of *PLPP4* and the long intergenic non-protein coding RNA genes 1561 (*LINC01561*) and 2930 (*LINC02930*), in cerebellum. rs71543507 had not been described as an eQTL at the time of writing.

According to GTEx, the average expression of *PLPP4* is highest in brain (especially in cerebellum, cerebellar hemisphere, hypothalamus, substantia nigra, and hippocampus), kidneys, and testes. *LINC01561* and *LINC02930* are also highly expressed in cerebellum. The expression pattern of *ENSG00000286565* is unknown, while *ACTR3B* is most highly expressed in brain, pituitary gland, and testes.

### Gene, gene set and tissue-level tests of association

Using the results of the meta-analysis, we aggregated all sequence variants by genes, gene-sets and GTEx tissue types and tested whether together they associated with social trust. No significant associations were found at the gene or tissue level, and only the gene set defined by the molecular function “AP-1 adaptor complex binding” (GO:0035650) showed association with trust with a Bonferroni-adjusted *p* value of 0.018 (Supplementary Tables 3, 4, 5). The AP-1 adaptor complex participates in clathrin-mediated endocytosis, which plays an important role in the recycling of synaptic vesicles and transmitter receptors in neurons^44^, among other pathways.

We then considered the subset of variants with association *p* values < 1e−05 in the meta-analysis and extracted the list of genes they mapped to using positional, eQTL and chromatin interaction mapping, as described in the Methods section. We considered this suggestive significance threshold because it is less stringent than its 5e−08 counterpart. We tested for tissue specificity by evaluating whether these genes were enriched in differentially expressed gene (DEG) sets, which did not reveal any significant results. When evaluating MsigDB^45^ gene-sets, we found several significant associations, many related to the nervous system. Some examples are the Gene Ontology sets “Olfactory Receptor Activity” (GO:0004984, adjusted *p* value 3.9e−19) and “Sensory Perception of Smell” (GO:0007608, adjusted *p* value 2.25e−18) and the Reactome sets “Signaling by GPCR” (R-HSA-372790, adjusted *p* value 3.14e−10) and “G Alpha (s) Signaling Events” (R-HSA-418555, adjusted *p* value 7.53e−15). The latter two include genes involved in vision, smell, behavioral regulation, functions of the autonomic nervous system, and regulation of the immune system. The full set of significantly enriched gene-sets can be found in the Supplementary Table 6.

### Genetic association with related traits

To further evaluate the credibility of our association signals, as well as to attempt to map social trust onto the existing genetic landscape, we examined the similarity of its genetic architecture to that of other psychological and psychiatric phenotypes previously investigated in large genetic cohorts.

**Figure 2:**
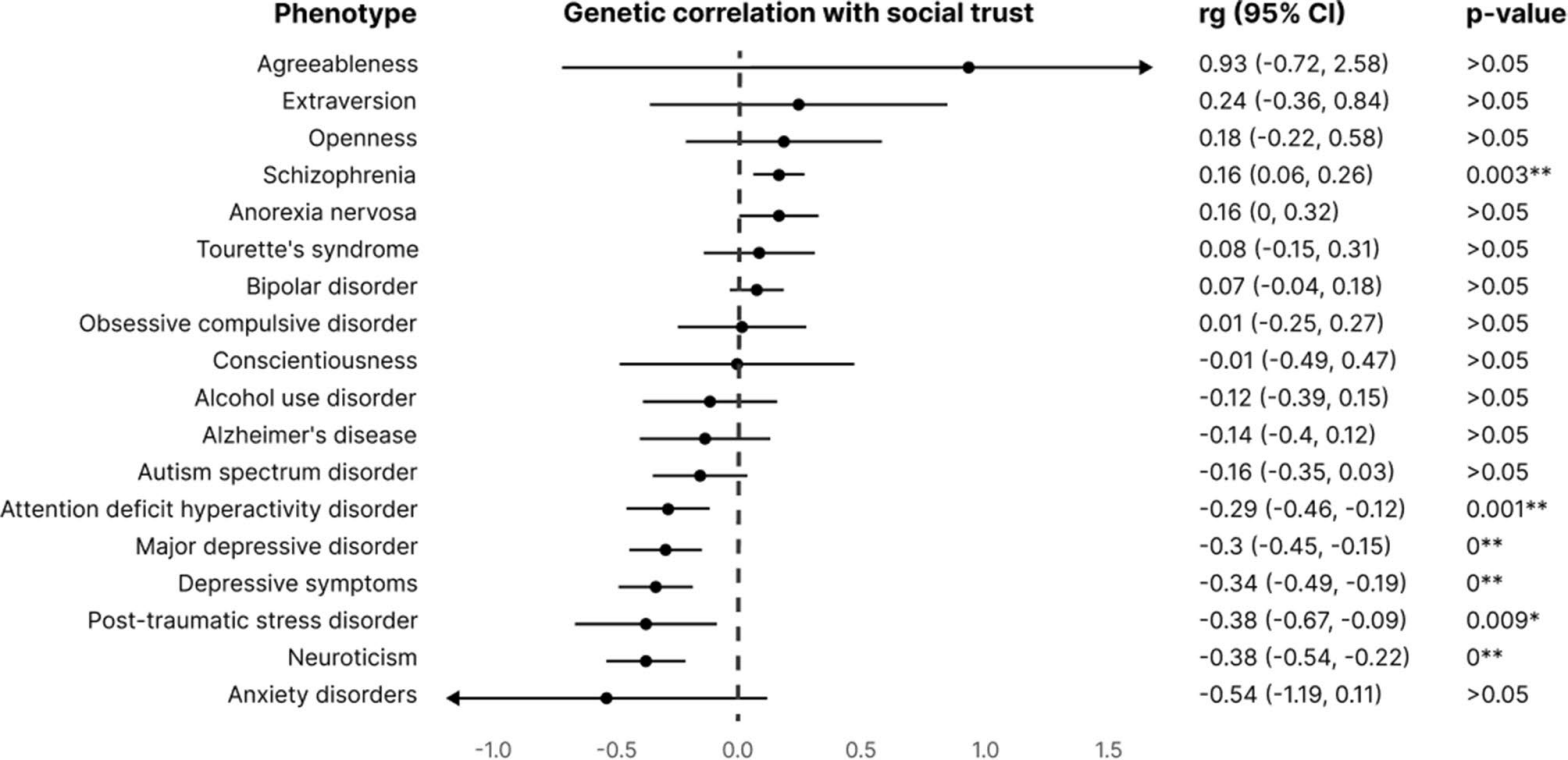
Genetic correlations between the social trust meta-analysis and 18 psychiatric and psychological traits. Correlation coefficients (rg, unbounded) and 95% confidence intervals (CI) are provided. *p* values shown are uncorrected. Genetic correlations that are significantly different from zero are marked with an asterisk (uncorrected) or two asterisks (Bonferroni-corrected for 18 tests).

At the genome-wide level, we computed the genetic correlations between the social trust meta-analysis and a non-exhaustive list of psychological and psychiatric traits (listed in the Methods section), as displayed in Fig. 2. Social trust revealed a significant negative genetic correlation with neuroticism (r_g_ = − 0.38; 95% CI = (− 0.54, − 0.22)), depressive symptoms (r_g_ = − 0.34; 95% CI = (− 0.49, − 0.19)), major depressive disorder (r_g_ = − 0.3; 95% CI = (− 0.45, − 0,15)) and attention deficit and hyperactivity disorder (ADHD; r_g_ = − 0.29; 95% CI = (− 0.46, − 0.12)), after Bonferroni-correcting for multiple testing. A nominally significant correlation was also found with post-traumatic stress disorder (PTSD; r_g_ = − 0.38; 95% CI = (− 0.67, − 0.09)). The genome-wide significant genetic correlation with schizophrenia was positive (r_g_ = 0.16, 95% CI = (0.06, 0.26), which is surprising because distrust is described as a common symptom in patients with the disorder^46,47^. Despite not being statistically significant, the genetic correlation with agreeableness was close to unity (r_g_ = 0.93, 95% CI = (− 0.72, 2.58)). References to the studies and sample sizes are provided in Supplementary Table 8. All pairwise correlations among the evaluated traits can be found in Supplementary Fig. 11.

**Figure 3:**
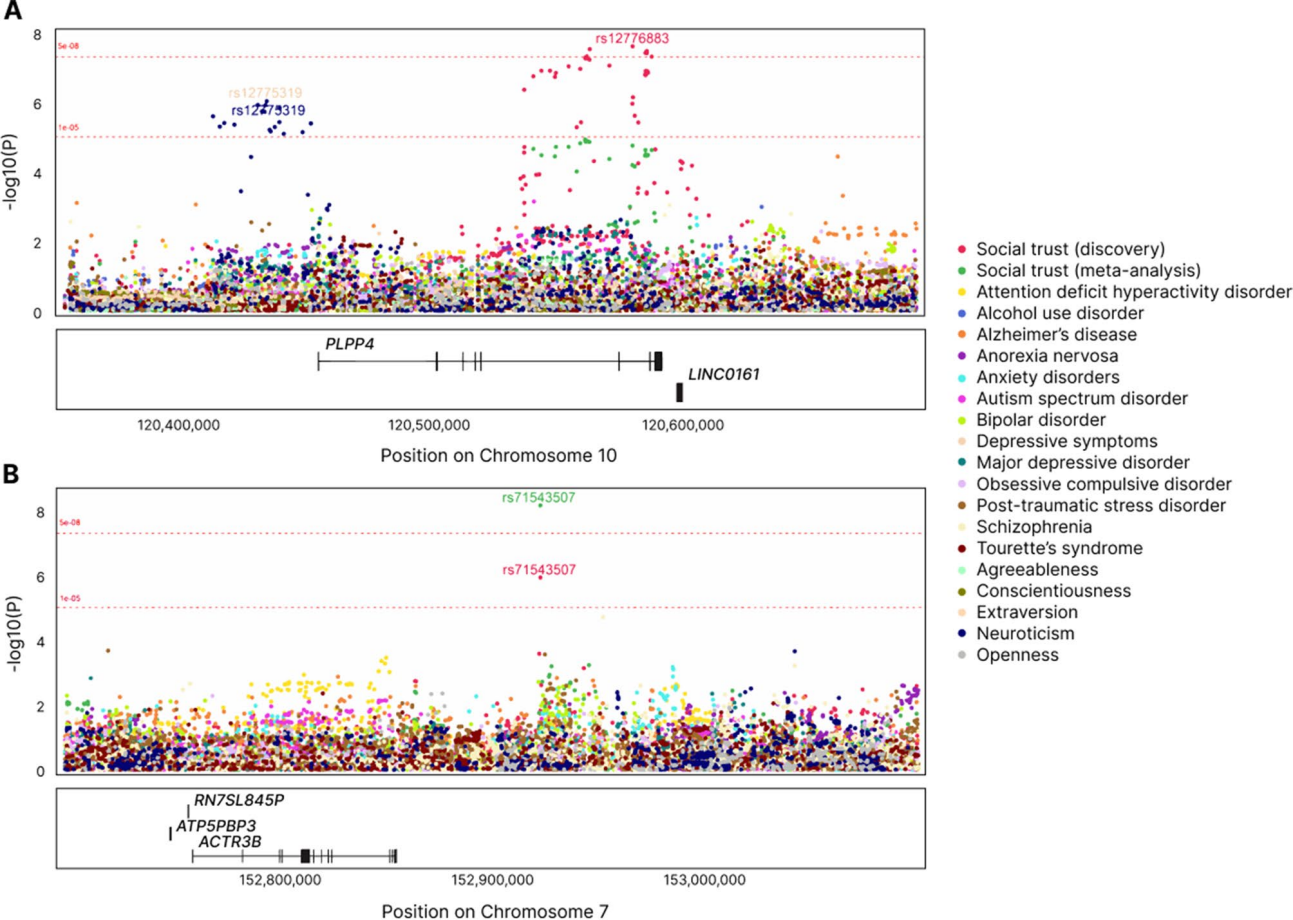
Regional patterns of association between genetic variants around the discovered loci, social trust, and a non-exhaustive list of psychological and psychiatric phenotypes. (**A**) Locus on chromosome 10 with lead variant rs12776883 as discovered in the discovery GWAS. (**B**) Locus on chromosome 7 with lead variant rs71543507 as discovered in the meta-analysis.

We also compared the pattern of association between sequence variants located around the two discovered loci, social trust and the same list of phenotypes used to compute the genetic correlations. We observed suggestive association signals (*p* values < 1e−05) between several variants directly upstream of *PLPP4* (LD r^2^ with lead signal < 0.1), neuroticism and depressive symptoms (Fig. 3A). No associations were found between variants around the meta-analysis discovered rs71543507 and any of the psychiatric or psychological phenotypes (Fig. 3B).

### Phenome-wide association study

We performed a Phenome-Wide Association Study (PheWAS) to investigate the association between our two discovered lead sequence variants and a broad set of phenotypes available in the Copenhagen Hospital Biobank Cardiovascular Disease Cohort^48^ (CHB-CVDC, n = 1241 International Classification of Diseases (ICD) -based Phecodes), as well as in studies including European populations stored in the GWAS Atlas resource (n = 3302 phenotypes in July 2023)^49^.

The discovery lead sequence variant, rs12776883, did not display association signals either in the GWAS Atlas or CHB-CVDC that were significant after correcting for multiple testing. In the GWAS Atlas, six out of the 16 associations with uncorrected *p* values < 0.01 were linked to neurological or psychiatric phenotypes, including measurements of different brain regions, sleep efficiency and major depressive disorder.

The meta-analysis lead sequence variant, rs71543507, did not display associations in the GWAS Atlas that were significant after correcting for multiple testing. Noticeably, eight out of the 13 associations with uncorrected *p* values < 0.01 were linked to neurological, psychiatric or cognitive phenotypes (e.g. feelings of distance or avoidance after a traumatic event; or the size of the orbitofrontal cortex, a region of the brain involved in reward, emotion and depression^50^); and two to cardiovascular phenotypes (atrial fibrillation in European and cross-ancestry studies). In CHB-CVDC, PheWAS associations were found with five musculoskeletal, infectious, digestive and pregnancy-related phenotypes, after correcting for multiple testing.

All PheWAS associations, significant or not, can be found in Supplementary Tables 9, 10, 11 and 12.

### Replication of previously associated genes

The genes *SLC6A4*^26,27^, *OXTR*^24,28–30^, *CD38*^29,31^ and *AVPR1A*^32^ have previously been associated with social trust or related social behavior phenotypes through genetic studies of different kinds. To test whether these observations replicated in our data, we generated regional association plots centered around these genes. We did not observe significant associations with the social trust meta-analysis, neither at the genome-wide Bonferroni corrected level nor at the more permissive 1e−05 *p* value threshold. Plots can be found in Supplementary Fig. 12.

## Discussion

In this study, we investigate the genetic underpinnings of social trust in n = 33,882 participants of the Danish Blood Donor Study (DBDS) using a validated questionnaire. We uncover insights both on the overall genetic architecture of the trait and on specific sequence variants that significantly associate with the phenotype in our cohort.

Through the largest GWAS of social trust to date, we provide an estimation of its SNP heritability, which amounts to 6% and is within the observed range among related phenotypes^51^. We discover a genome-wide pattern of genetic association with trust that correlates with that of other psychological and psychiatric phenotypes, i.e. negatively with neuroticism, depressive symptoms, major depressive disorder, and ADHD, and positively with schizophrenia. Albeit not statistically significant, the genetic correlation between social trust and agreeableness is estimated to be close to unity, which is consistent with the Big Five model of personality, where trust is a subdomain of agreeableness. We also find that several gene sets involved in the nervous system are overrepresented in this signal.

We find evidence of two genome-wide associated loci, but acknowledge that their credibility is limited by the lack of resources available for robust replication.

The first locus was observed in our discovery GWAS (n = 25,819) and harbors six sequence variants (lead variant: rs12776883) in an intronic enhancer region of *PLPP4*. This protein-coding gene is highly expressed in brain, kidneys, and testes^43^, and has previously been associated with cognitive decline in Alzheimer's and Parkinson's diseases^52,53^, among other conditions. In our work, we find that several variants in this locus and others mapping to the same gene are also suggestively associated with neuroticism, depressive symptoms and other cognitive, psychiatric and neurological phenotypes . When social trust was evaluated a year later in an independent subset of the DBDS (n = 8063), this locus showed no signal of association. Given the considerably different social situation in the country due to the progression of the COVID-19 pandemic, which was characterized by minimal interpersonal contact and increased loneliness^54,55^, the question remains whether this lack of replication reveals a false positive or a gene-environment interaction. Larger cohorts and better mapping of putative confounders may help untangle this.

The second signal of association was observed in the subsequent meta-analysis and consisted of a single sequence variant (rs71543507) in an intergenic enhancer region between *ENSG00000286565,* a novel non-characterized long intergenic non-protein coding RNA gene, and *ACTR3B*, a protein-coding gene highly expressed in brain, pituitary, and testes^43^. We find that the variant also displays suggestive associations with other neurological, psychiatric and cognitive phenotypes related to reward, emotion and depression in independent cohorts . The expression patterns of both *ACTR3B* and *PLPP4* are consistent with their suspected implication in behavior and possibly with their connection with physical health through the postulated responsible biological pathway, the HPA axis of stress regulation^6,56,57^.

In the only other GWAS of social trust found^24^, none of our discovered sequence variants are among the top 50 associations reported . We are also unable to find any signals of association with sequence variants in or around the genes previously highlighted as potentially involved in social behavior and trust . More studies are needed to elucidate whether this is due to the diverse conceptualizations and measurement methodologies that have been applied to this multidimensional phenotype. Alternatively, population differences like the slightly higher median level of social trust in the blood donors, more altruistic compared to the general Danish population^58^, or the overall higher level of trust in Denmark with respect to other countries^35^, could be causing an overestimation of the genetic influence on social trust or resulting in a poor replicability of results.

The main limitation of this study is inherent to the complexity^59^ and often high genetic heterogeneity^60^ of psychological and psychiatric traits, as decades of genetic research have shown. Many of these traits, including social trust, may constitute the visible manifestations of other upstream, more neurologically inherent phenotypes, such as the tendency towards negative cognitive biases, a lower stress activation threshold or risk aversion^61^. Together with the non-additive and gene-environment effects that are unaccounted for in GWAS, this results in low SNP-based heritability estimates and represents a currently unresolved challenge in studying the phenotypes’ genetic architectures. Considerably larger sample sizes are typically required to detect associations with variants of modest effect in complex phenotypes like ours – a good example is the GWAS of educational attainment, which found only four associated loci when the sample size was 126,559, but 3952 loci when it reached three million samples^62^. In addition, the DBDS is a highly selected population, mostly composed of individuals of Northern European ancestry in good health at the time of inclusion, which may create a selection bias and limit the generalizability of our results. Finally, our work focuses on common variation, and the role of rare variants is yet to be investigated.

In conclusion, this study adds to the evidence that social trust is influenced by complex genetic factors and demonstrates the potential of our approach to start uncovering the underlying neurobiology of human social behavior. We are now one step closer to a genetic instrument of social trust that can be used to investigate genetic correlations to other traits, including physical health. We encourage large genomic cohorts to perform equivalent analyses to improve our understanding of the genetic basis of social trust. Further research could have significant implications across fields such as social and political sciences, public health, and medicine.

## Methods

### Study population

This study utilizes data from the Danish Blood Donor Study (DBDS)^33,34^, a large prospective cohort of blood donors. Initiated in 2010, the study aims to identify predictors of health and disease among blood donors in Denmark. Numerous data types have been collected, both self-reported and registry based. Study participants’ sex (female/male) and birth year were retrieved from the Danish Civil Registration System^63^. In addition, whole-genome genotype data are available for around 114,000 participants of the DBDS Genomic Cohort. A detailed description of this cohort is provided elsewhere^33^.

Our discovery sample is a subset of the DBDS Genomic Cohort, which includes n = 25,819 individuals for which self-reported levels of social trust were collected in May 2021. Participation was voluntary and not rewarded. For internal replication, we used a smaller, independent sample of n = 8063 DBDS participants that were phenotyped around one year later (July 2022). A flowchart of sample inclusion can be found in Supplementary Fig. 1.

We used data from the Copenhagen Hospital Biobank Cardiovascular Disease Cohort (CHB-CVDC)^48^ to conduct the PheWAS. The CHB-CVDC is a genomic cohort based on EDTA blood samples collected from patients for blood typing and red cell antibody screening at hospitals in the Greater Copenhagen Area^64^. Participants in CHB-CVDC are above 18 years of age and have been assigned at least one cardiovascular diagnosis according to the Danish National Patient Registry. The cohort includes older participants with a long follow-up time and phenotypes that are systematically recorded in national health records, making it a great resource for identifying associations with diseases at any age of onset.

### Ethical approval

The study was approved by the National Committee on Health Research Ethics (DBDS: 1700407; CHB-CVDC: 1708829, ‘Genetics of CVD’—a genome-wide association study on repository samples from CHB). All DBDS participants provided informed consent to participate. We confirm that all methods were carried out in accordance with relevant guidelines and regulations.

### Phenotype

Data on self-reported levels of social trust (or generalized trust), were collected through two rounds of questionnaires sent through digital mail (e-Boks) to participants of the DBDS, in May of 2021 and July of 2022. The response rates were 26% in the first round and 13.5% in the second (Supplementary Fig. 1). Three items taken from the European Social Survey were used to capture the trust phenotype (https://ess-search.nsd.no/variable/query/trust/). They were originally designed to measure trust as an individual-level attitude^35^, and are constructed on a scale from one to ten, with higher scores reflecting higher levels of trust. The three questions were translated into Danish from English and have previously been validated in both languages^65,66^. Figure 1B holds the list of questions with the original English wording. The average of the responses to the three items constituted the social trust phenotype used throughout this study. In order to test the dimensionality of the construct captured by the three items, we performed principal component analysis (PCA) using the *stats* package^67^ in R^68^. RStudio^69^ and the *tidyverse* package^70^ were used extensively for data handling throughout this work.

### Genetic analyses

Our GWAS were based on imputed whole genome data, genotyped by deCODE genetics, using the Infinium Global Screening Array by Illumina. These data underwent standard quality control and imputation using an in-house reference backbone of North-Western European, whole genome sequences, including approximately 8000 Danish samples, as described elsewhere^33^. For the GWAS we used REGENIE v3.1^71^.

We fitted an ordinal model to the trust phenotype and used the normally distributed surrogate residuals for the analysis^72^. Year of birth, sex, geographic region within Denmark, questionnaire submission time (delta to first submission), and an indicator of the presence of COVID-19 symptoms at the time of submission were included as covariates when calculating the residuals. The last three were added in an effort to remove COVID-19 related effects, as the first round of questionnaires were circulated in May of 2021, parallel to the lifting of restrictions after the second national lockdown in Denmark. Year of birth and questionnaire submission delta were modeled as restricted cubic splines to allow for non-linearity. The effects of these covariates on social trust according to the model can be found in Supplementary Figs. 3 and 4.

Population structure was accounted for through principal components (PC) using PCA performed on 30,730 independent sequence variants with MAF larger than 5% in our study population (*flashpca* R package^73^). We observed that the proportion of variance explained plateaued at the sixth PC (Supplementary Fig. 7); therefore, we included the first six PCs to capture the structure of the population, while limiting the complexity of the model.

Genomic inflation was evaluated with λ_GC_, LD Score (LDSC)^74^ regression intercepts and quantile–quantile (QQ) plots of the association *p* values. The genomic inflation factor λ_GC_ is defined as the median of the observed chi-squared test statistics divided by the expected median of the chi-squared distribution with one degree of freedom. The LD Score regression intercept further takes into consideration the LD structure among sequence variants to produce a more accurate estimation of the genomic inflation. We used the LD Scores from the 1000 Genomes Project^75^ EUR panel as reference, which has been deemed appropriate for populations of predominantly northern European ancestry^74^. The three methods detected minimal inflation in the discovery (λ_GC_ = 1.023; LDSC intercept = 1.009 (95% CI = (0.997, 1.021))), replication (λ_GC_ = 1.01; LDSC intercept = 0.999 (95% CI = (0.985, 1.013))), and meta-analysis (λ_GC_ = 1.02; LDSC intercept = 0.984 (95% CI = (1.004, 1.036))) GWAS. QQ plots can be found in the Supplementary Figs. 6, 8 and 9.

The genome-wide significance threshold was set at the Bonferroni corrected 5e−08. For selected functional analyses we used the less stringent 1e−05 threshold, like others have done before. Lead variants within a genomic locus were chosen as those with the lowest *p* value. All variants within a genomic locus had an LD r^2^ > 0.6 with the lead. Genome-wide significant variants were filtered again for imputation quality > 0.9, genotype missingness < 0.1, and Hardy–Weinberg Equilibrium (HWE, no significant heterozygote excess at the 5% significance level). Observed MAFs were compared with those of other European populations as provided by gnomAD^36^ and with a Danish reference genome panel (DanMAC5)^37^ built from 8671 whole genome sequences (Supplementary Table 2). Data filtering, MAF calculations and tests of HWE were performed using PLINK v2.0^76^.

### Internal replication

We performed a GWAS on the replication cohort following the same steps as with the discovery cohort. Using the methodology described further down, we computed the genome-wide genetic correlation between the results of the discovery and the replication GWAS.

### Meta-analysis

Using METAL^77^, we meta-analyzed the results of the discovery and internal replication GWAS with the aim of increasing statistical power. The analysis was performed on the set of high imputation quality (score > 0.9), bi-allelic sequence variants available in both the discovery and replication datasets (n ~ 7.9 M) using the inverse variance weighted approach. We also applied METAL’s inbuilt genomic control to correct for any unaccounted population stratification or relatedness.

### External replication

We evaluated the concordance between our meta-analyzed GWAS results and those of the only other GWAS on social trust by Wooton et al.^24^, comparing *p* values and effect sizes for the top 50 variants reported in their study. A total of 44 were available in our data.

### Functional annotation and gene mapping

We functionally annotated our lead sequence variants' predicted regulatory activity with RegulomeDB^39^, deleteriousness with scaled CADD scores^40^, and chromatin state with ChromHMM^41^. We used the GTEx v8^42,43^ resource to find whether these variants had been recorded as eQTLs.

In order to map each variant to candidate genes, we selected those closest to them in the genome sequence and those linked through eQTL activity. In the case of rs71543507, we report the two closest genes, as the first is a very poorly characterized long intergenic non-protein coding RNA gene not available in the NCBI RefSeq^38^ dataset. We checked the tissue-specific expression pattern of these genes in GTEx.

### Gene, gene set and tissue-level tests of association

Using the implementation of MAGMA^78^ in the FUMA GWAS platform^49^, all sequence variants were aggregated into genes, gene sets and GTEx tissues, and their association with social trust in the meta-analysis was further evaluated. The list of candidate genes which sequence variants with *p* values < 1e−05 in the meta-analysis mapped to was also tested for enrichment in differentially expressed gene (DEG) sets and MsigDB and C1-8 gene sets. In all MAGMA analyses, Bonferroni correction of *p* values was applied according to the number of tests performed. Variant-to-gene mapping was performed by FUMA based on positional, eQTL and chromatin interaction data (datasets used are specified in Supplementary Tables 4, 5, 6). Please refer to FUMA's documentation online for more details (https://fuma.ctglab.nl/tutorial).

### SNP heritability

LD Score regression (LDSC)^74,79^ was used to estimate the SNP heritability (h^2^_SNP_) of social trust in the discovery GWAS and the meta-analysis. As recommended in the LDSC documentation, we filtered our sequence variants to match the HapMap3 set, removed ambiguous alleles and used the pre-computed LD Scores from the 1000 Genomes Project^75^ EUR panel as reference. This reference has previously been deemed appropriate for populations of predominantly Northern European ancestry^74^.

### Genetic association with related traits

We also used LDSC to compute genome-wide genetic correlations between our social trust meta-analysis and a non-exhaustive list of psychiatric phenotypes with summary statistics publicly available on the Psychiatric Genomics Consortium portal (https://www.med.unc.edu/pgc/). We performed the same filtering steps as are described above. LDSC’s correlation coefficient estimator is unbounded, and can lie outside of the [− 1,1] range when the heritability estimate of one or both of the phenotypes is low. We included the phenotypes of alcohol use disorder (AUD), attention deficit and hyperactivity disorder (ADHD), Alzheimer’s disease (AD), anorexia nervosa (AN), anxiety disorders (ANX), autism spectrum disorder (ASD), bipolar disorder (BIP), depressive symptoms (DEP), major depressive disorder (MDD), neuroticism (NEU), obsessive–compulsive disorder (OCD), post-traumatic stress disorder (PTSD), schizophrenia (SCZ) and Tourette’s syndrome (TS). In addition, we included the most recent and publicly available GWAS results of the five main domains of personality as defined by the Revised NEO Personality Inventory^80^: openness (OPN), conscientiousness (CONS), extraversion (EXTR), agreeableness (AGR) and neuroticism (NEU). References and sample sizes can be found in Supplementary Table 8. *p* values were Bonferroni-corrected to account for the 18 statistical tests. In the Supplementary Fig. 11, we provide all pairwise genetic correlations among the 19 phenotypes, which can be used to check for consistency.

GWAS signals around the discovered risk loci were also explored through regional plots of association covering *PLPP4* ± 100 KB and rs71543507 ± 200 KB. Overlapping signals for social trust with the aforementioned list of psychiatric and psychological phenotypes allowed detection of similarities in the local pattern of genetic association. Regional plots were generated with the *topr* R package^81^.

### Phenome-wide association study

We performed a Phenome-Wide Association Study (PheWAS) to test the association of our candidate sequence variants with a broader set of phenotypes available in the Copenhagen Hospital Biobank Cardiovascular Disease Cohort^48^ (CHB-CVDC, n = 1241 International Classification of Diseases (ICD)-based Phecodes), as well as in studies including European populations stored in the GWAS Atlas resource (n = 3302 phenotypes)^49^. Statistical significance in the CHB CVDC PheWAS was evaluated after Bonferroni multiple testing correction, where the number of independent tests was taken as the product of the number of principal components that captured 99.5% of the phenotypic variance and the number of tested variants after pruning for LD. The GWAS Atlas PheWAS was done directly on the platform and the number of independent tests was equal to the total number of studies included.

### Replication of previously associated genes

We graphically evaluated the link between the genes previously highlighted in the context of social trust and our measure of this phenotype in the meta-analysis by generating regional plots of association. The genes in question were taken from the literature and were selected based on the criteria that they had been associated with social trust or related social behavior phenotypes through genetic studies of any kind, with candidate gene studies being the most frequent. The genes included were the serotonin transporter gene (*SLC6A4*), the oxytocin receptor gene (*OXTR*), *CD38* and the arginine vasopressin receptor 1A gene (*AVPR1A*).

### Protein interactions with PLPP4 and ACTR3B

We used the STRING database^82^ to evaluate the network of proteins *PLPP4* and *ACTR3B* interact with in the human proteome. The other mapped genes were not assessed as they do not code for proteins. STRING provides both physical and functional interactions extracted and/or inferred from a variety of sources like annotated databases, laboratory experiments, co-expression, phylogenetic co-occurrence, automated text-mining and more. Interactions and annotations can be found in Supplementary Figs. 13, 14 and Supplementary Tables 13, 14, 15 and 16.

### Supplementary Information

Supplementary Figures.

Supplementary Tables.

## Data Availability

All summary statistics will be made available via the GWAS Catalog. The data that support the findings of this study are available from the DBDS, but restrictions apply to the availability of these data, which were used under license for the current study, and so are not publicly available. Data are however available with permission of the DBDS steering committee and the national scientific ethical committee.
