# Supplementary material for: A genome-wide association study of social trust in 33,882 Danish blood donors
Source: Sci Rep. 2024 Jan 16;14:1402. doi: 10.1038/s41598-024-51636-0 (PMC10792163; doi:10.1038/s41598-024-51636-0)

A genome-wide association study of social trust in 33,882 Danish Blood Donors

Celia Burgos Sequeros[1], Thomas Folkmann Hansen[1,2], David Westergaard[1,3,4], Ioannis Louloudis[1], Sebastian Kalamajski[5], Timo Röder[1], Palle Duun Rohde[6], Michael Schwinn[7], Line Harder Clemmensen[8], Maria Didriksen[7], Mette Nyegaard[6], Henrik Hjalgrim[9,10], Kaspar René Nielsen[11], Mie Topholm Bruun[12], Sisse Rye Ostrowski[7,10], Christian Erikstrup[13,14], Susan Mikkelsen[13], Erik Sørensen[7], DBDS genomic consortium, Ole Birger Vestager Pedersen[10,11], Søren Brunak[1], Karina Banasik[*,§,1,4], Giuseppe Nick Giordano[§,5]

1. Translational Disease Systems Biology, Novo Nordisk Foundation Center for Protein Research, Faculty of Health and Medical Sciences, University of Copenhagen, Copenhagen, Denmark
2. Department of Neurology, Danish Headache Center Copenhagen University Hospital - Rigshospitalet, Glostrup, Denmark
3. Methods and Analysis, Statistics Denmark, Copenhagen, Denmark
4. Department of Gynecology and Obstetrics, Copenhagen University Hospital Hvidovre, Copenhagen, Denmark
5. Genetic and Molecular Epidemiology Unit, Lund University Diabetes Centre, Department of Clinical Sciences, CRC, Malmö, Sweden
6. Genomic Medicine, Department of Health Science and Technology, Aalborg University, Gistrup, Denmark
7. Department of Clinical Immunology, Copenhagen University Hospital - Rigshospitalet, Copenhagen, Denmark
8. Department of Applied Mathematics and Computer Science, Technical University of Denmark, Kongens Lyngby, Denmark
9. The Danish Cancer Institute; Department of Epidemiology Research, Statens Serum Institut; Department of Haematology, Rigshospitalet, Denmark
10. Department of Clinical Medicine, Faculty of Health and Medical Sciences, University of Copenhagen, Copenhagen, Denmark
11. Department of Clinical Immunology, Zealand University Hospital, Køge, Denmark
12. Clinical Immunology Research Unit, Department of Clinical Immunology, Odense University Hospital, Odense, Denmark
13. Department of Clinical Immunology, Aarhus University Hospital, Aarhus, Denmark
14. Department of Clinical Medicine, Aarhus University, Aarhus, Denmark

*Corresponding author: Karina Banasik, karina.banasik@cpr.ku.dk

§Authors contributed equally

Supplementary Figures

Supplementary Figure 1. Flowchart of sample inclusion..3
Supplementary Figure 2. Social trust in the DBDS 2022 replication cohort. 4
Supplementary Figure 3. Effect of sex, year of birth and other covariates on social trust, when measured in May 2021. 5
Supplementary Figure 4. Effect of sex, year of birth and other covariates on social trust, when measured in July 2022. 5
Supplementary Figure 5. Principal component analysis of social trust questionnaire responses. 6
Supplementary Figure 6. Genetic associations with social trust, as found through the discovery GWAS..7
Supplementary Figure 7. Assessment of genetic population substructure. 8
Supplementary Figure 8. Genetic associations with social trust, as found through the replication GWAS..9
Supplementary Figure 9. Genetic associations with social trust, as found through the meta-analysis. 10
Supplementary Figure 10. Associations between sequence variants around *PLPP4* and social trust in 2021, 2022 and meta-analysis. 11
Supplementary Figure 11. Genetic correlations among social trust and 18 psychiatric and psychological traits. 12
Supplementary Figure 12. Regional plots of association between social trust meta-analysis and sequence variants around the main genes previously linked to social trust. 13
Supplementary Figure 13. Network of protein interactions including *PLPP4* as obtained from the STRING database. 14
Supplementary Figure 14. Network of protein interactions including *ACTR3B* as obtained from the STRING database. 15

Supplementary Figure 1. Flowchart of sample inclusion. On the left is the sample inclusion for the discovery genome-wide association study (GWAS) of social trust (Round one) measured in 2021. On the right, that for the replication GWAS of social trust (Round two) measured in 2022.

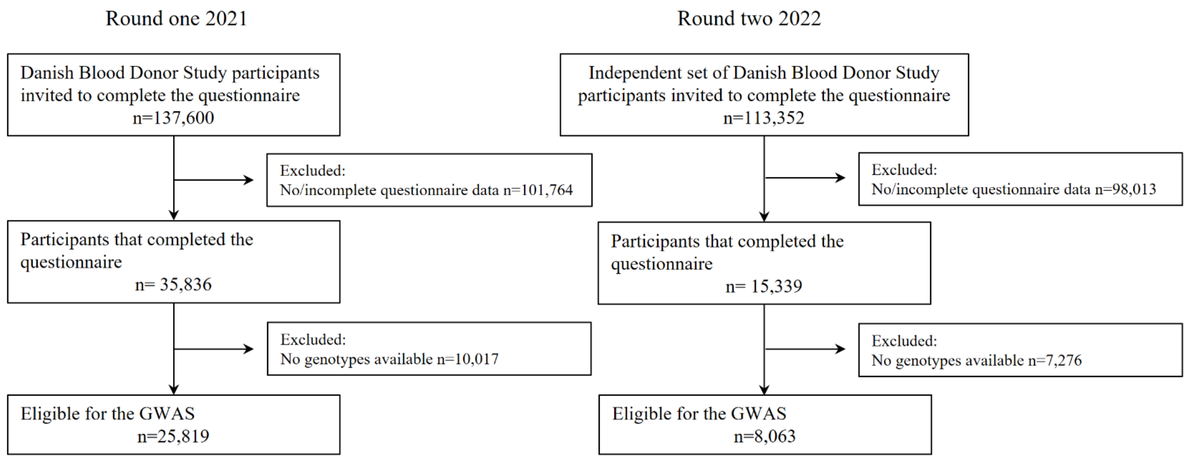

Supplementary Figure 2. Social trust in the DBDS 2022 replication cohort. (A) Sex and year of birth distributions. Solid lines represent the median year of birth (1968 and 1965 for male and female participants, respectively); dashed lines represent the 25th and 75th quartiles. (B) The three social trust questionnaire items and their answers in the DBDS (grey bars; n = 25,819; median in red) and a random sample of the Danish population (black line; n = 10,369; source: European Social Survey; median in black).

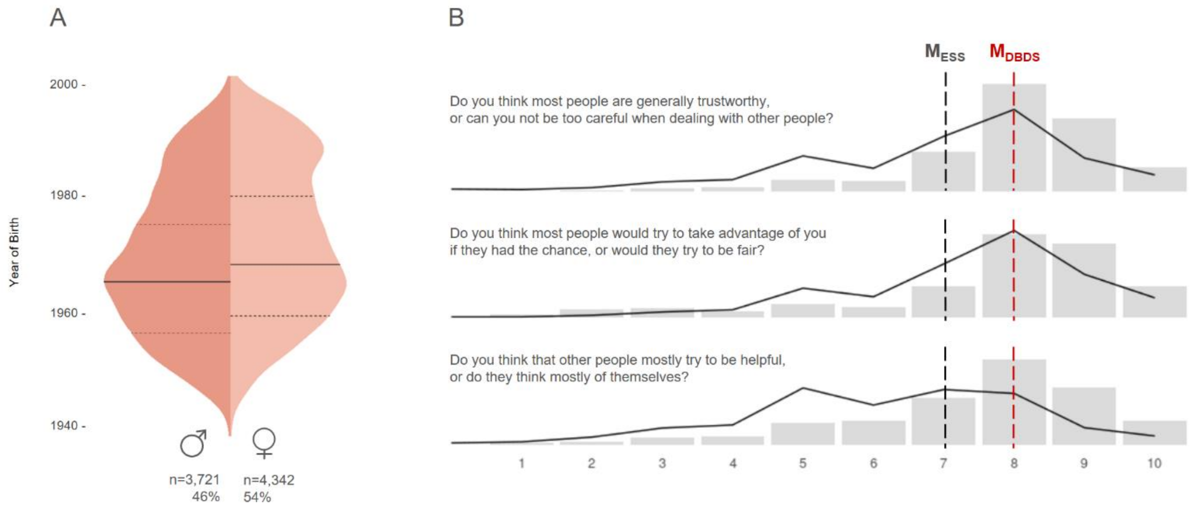

Supplementary Figure 4. Effect of sex, year of birth and other covariates on social trust, when measured in May 2021. The plots shows the log odds ratios of sex, year of birth (yob), relative questionnaire submission time (delta to first submission), an indicator of the presence of COVID-19 symptoms at the time of submission (persist) and the geographic region within Denmark (Region M, Region H, Region N, Region S, Region SJ) on social trust. Both year of birth and submission time delta were modeled as restricted cubic splines to allow for non-linearity.

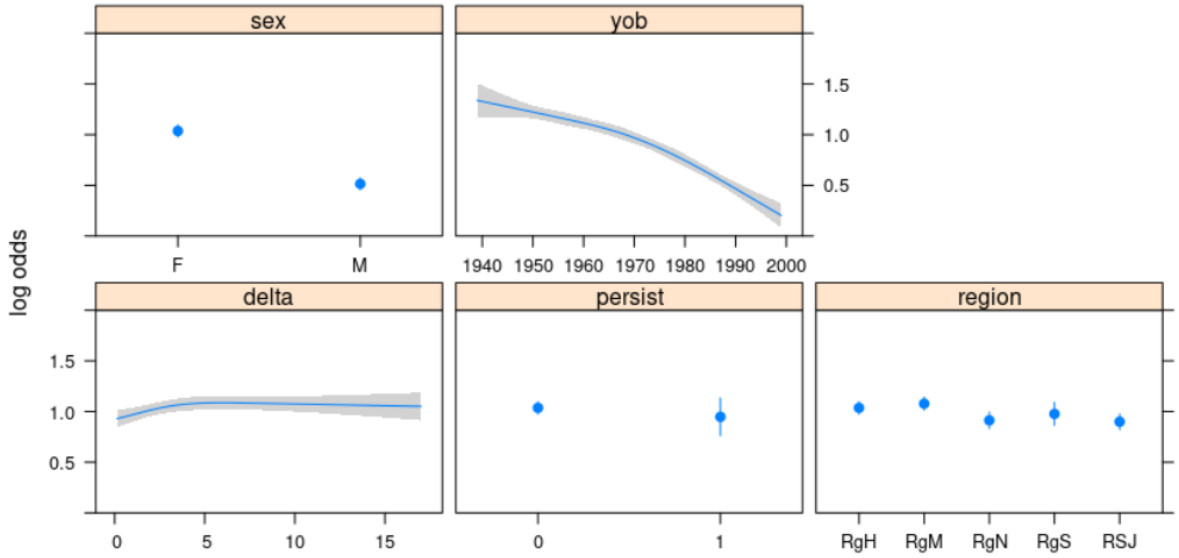

Supplementary Figure 3. Effect of sex, year of birth and other covariates on social trust, when measured in July 2022. The plots shows the log odds ratios of sex, year of birth (yob), relative questionnaire submission time (delta), an indicator of the presence of COVID-19 symptoms at the time of submission (persist) and the geographic region within Denmark (Region M, Region H, Region N, Region S, Region SJ) on social trust. Both year of birth and submission time delta were modelled as restricted cubic splines to allow for non-linearity.

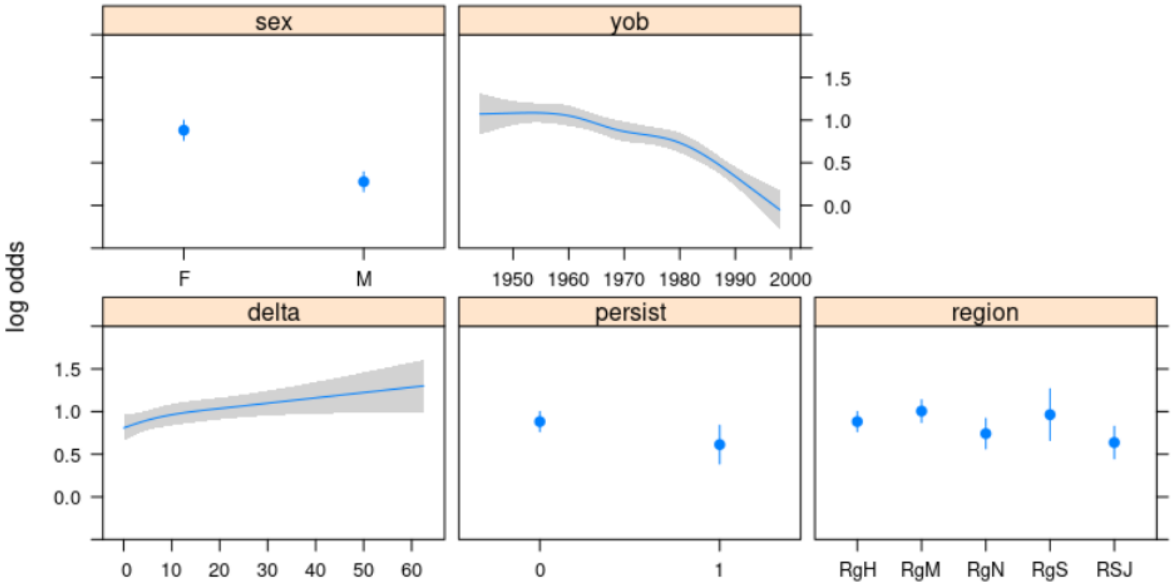

Supplementary Figure 5. Principal component analysis of social trust questionnaire responses. (A) Proportion of variance explained by PC1-3. (B) PC loadings of each of the three questionnaire items, equivalent to the correlation among each item-PC pair.

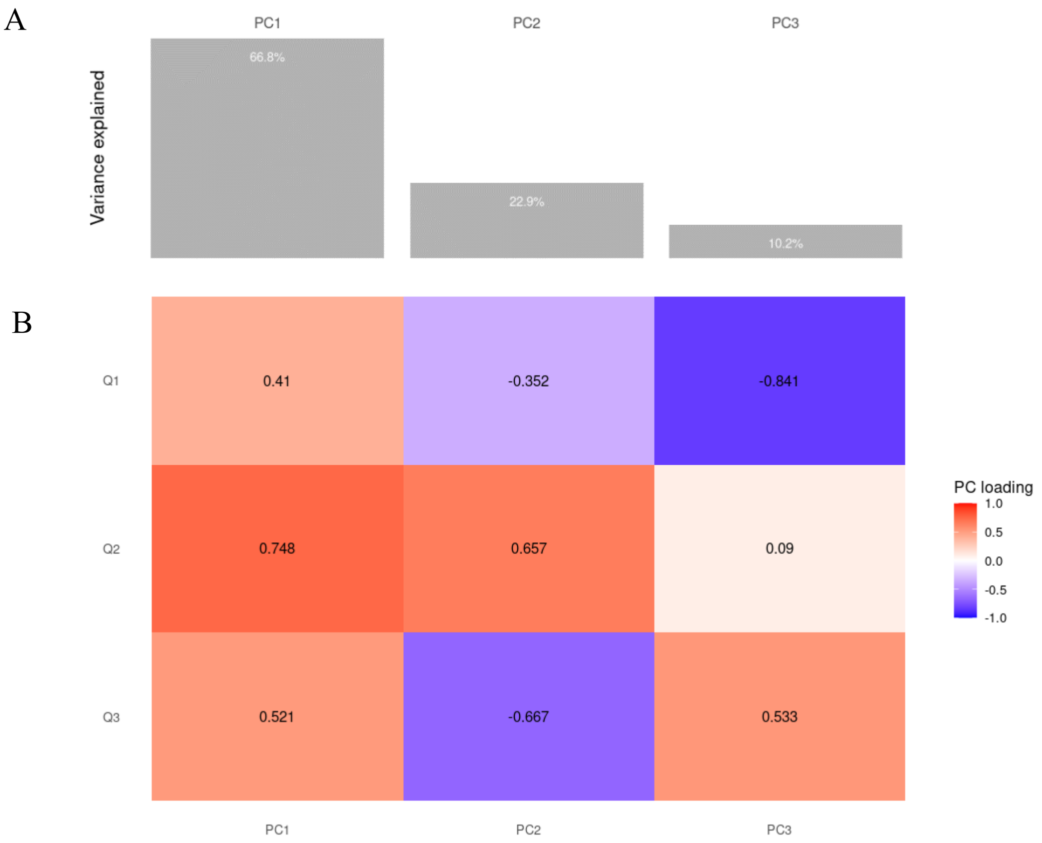

Supplementary Figure 6. Genetic associations with social trust, as found through the discovery GWAS. (A) Manhattan plot showing -log10 p-values for variant associations. The horizontal red and blue lines represent the Bonferroni-corrected genome-wide significance threshold of 5e-08 and the suggestive significance threshold 1e-05. (B) Quantile-quantile plot of the distribution of p-values, colored by minor allele frequency range. Lambda GC and LD score regression intercept are shown. (C) Regional plot of association between sequence variants around PLPP4 and social trust. Above, -log10(p-values); in the middle, effect sizes and their 95% confidence intervals, filtered by p-value ≤ 1e-05 and colored by -log10(p-value); below, LD (r^2) of variants in the region with the lead, rs12776883. Only NCBI RefSeq genes are shown.

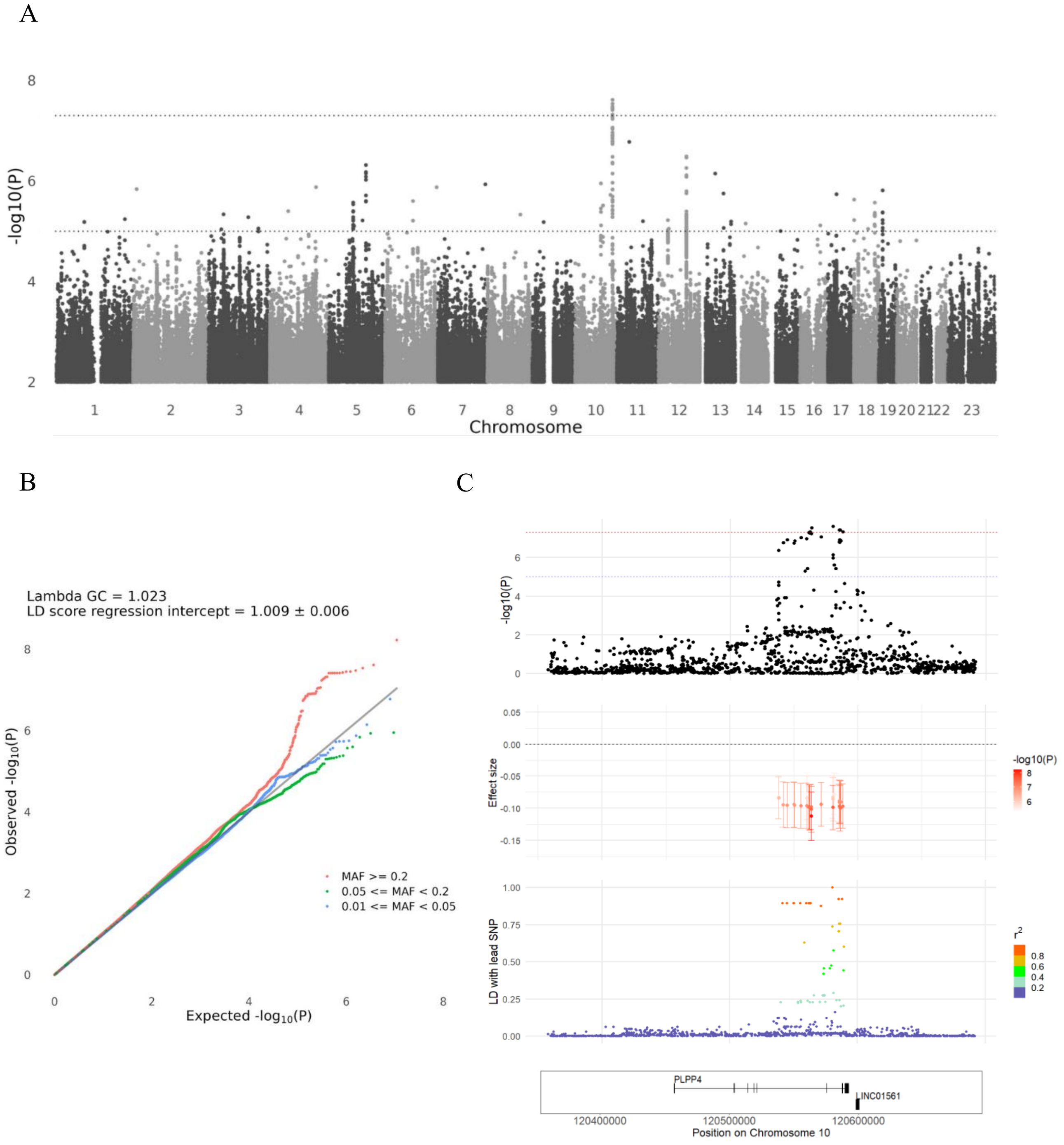

Supplementary Figure 7. Assessment of genetic population substructure. Proportion of variance explained by the principal components (PCs) computed on 30,730 independent genetic variants with minor allele frequencies (MAF) larger than 5% in our population, as described in the Methods. Based on this graph and the elbow of the curve, we argue that the first six PCs are enough for capturing most of the genetic population substructure and including it as covariate in the GWAS.

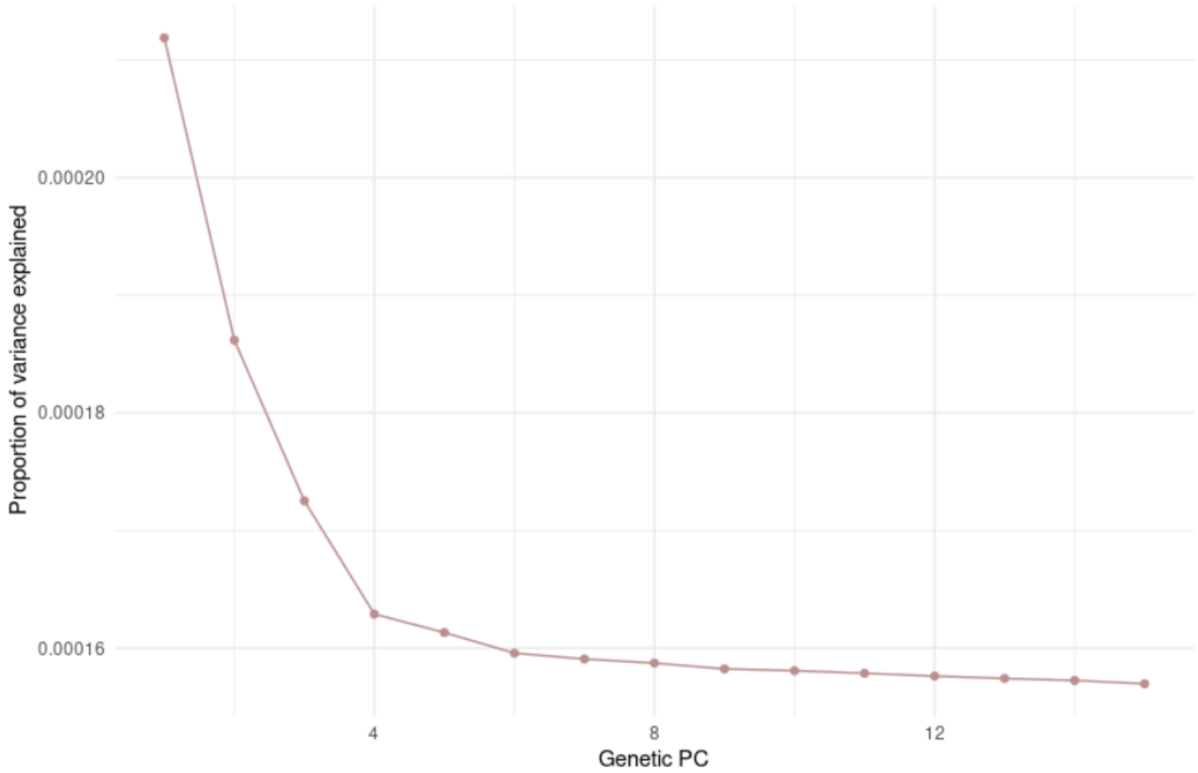

Supplementary Figure 8. Genetic associations with social trust, as found through the replication GWAS. (A) Manhattan plot showing -log10 p-values for variant associations. The horizontal lines represent the Bonferroni-corrected genome-wide significance threshold of 5e-08 and the suggestive significance threshold 1e-05. (B) Quantile-quantile plot of the distribution of p-values, colored by minor allele frequency range. Lambda GC and LD score regression intercept are shown.

A

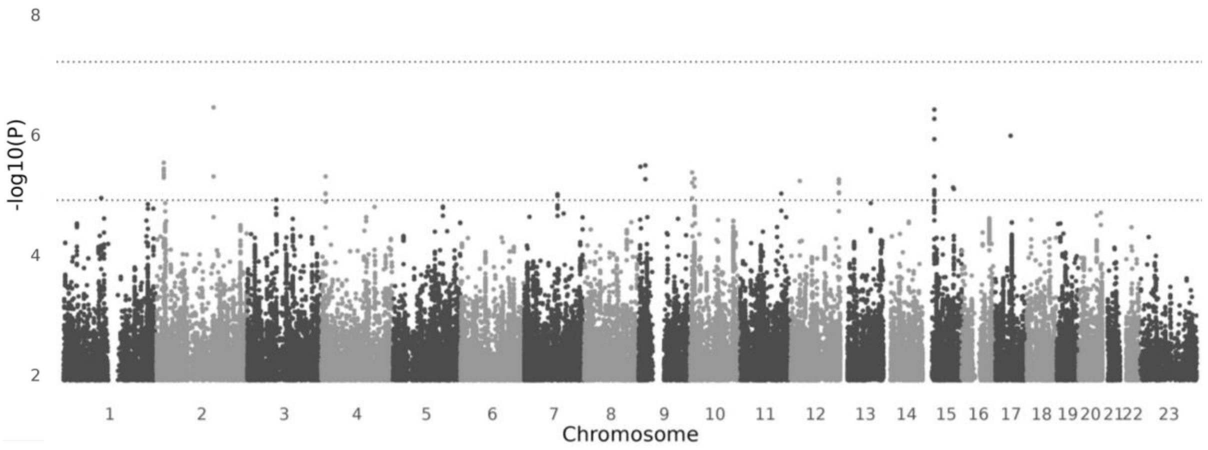

B

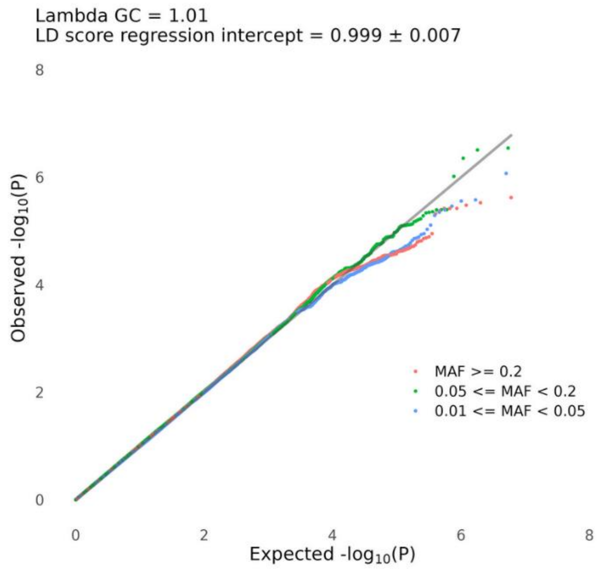

Supplementary Figure 9. Genetic associations with social trust, as found through the meta-analysis. (A) Manhattan plot showing -log10 p-values for variant associations. The horizontal lines represent the Bonferroni-corrected genome-wide significance threshold of 5e-08 and the suggestive significance threshold 1e-05. (B) Quantile-quantile plot of the distribution of p-values, colored by minor allele frequency range. Lambda GC and LD score regression intercept are shown. (C) Regional plot of association between sequence variants around the only genome-wide significant signal and social trust. Above, -log10(p-values); in the middle, effect sizes and their 95% confidence intervals, filtered by p-value <= 1e-e05; below, LD (r^2) of variants in the region with the lead SNP, rs71543507. Only NCBI RefSeq genes are shown.

A

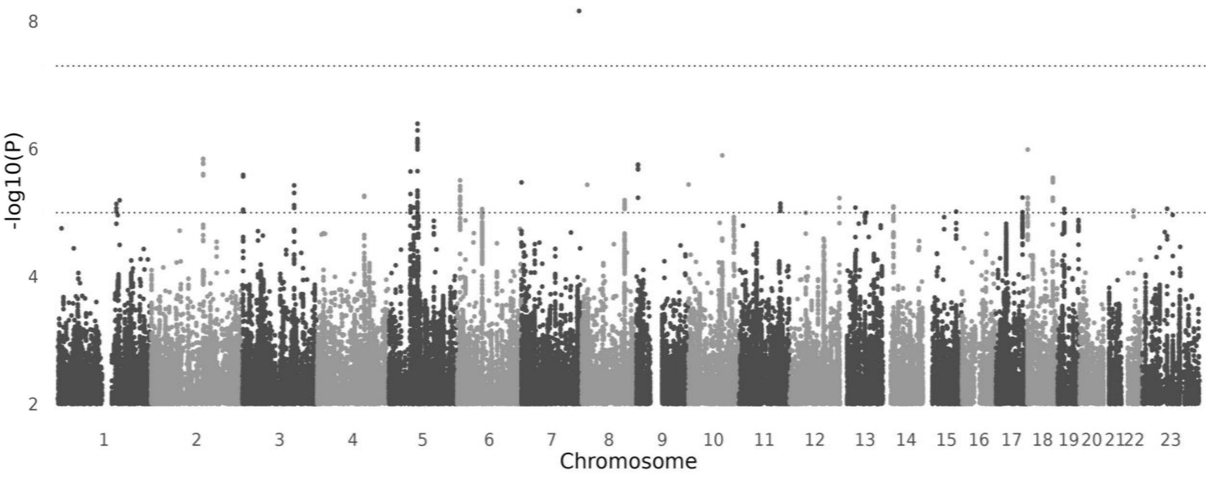

B

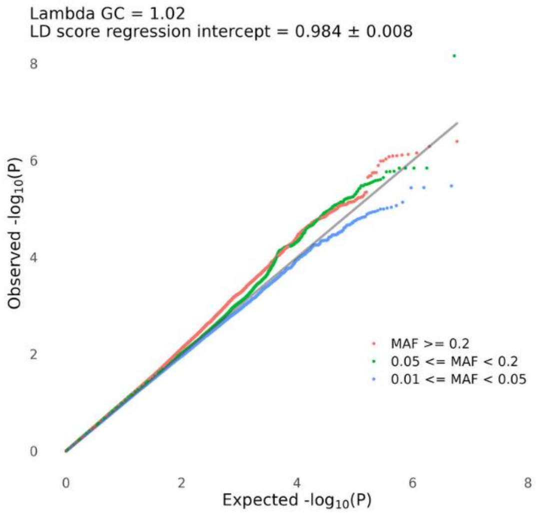

C

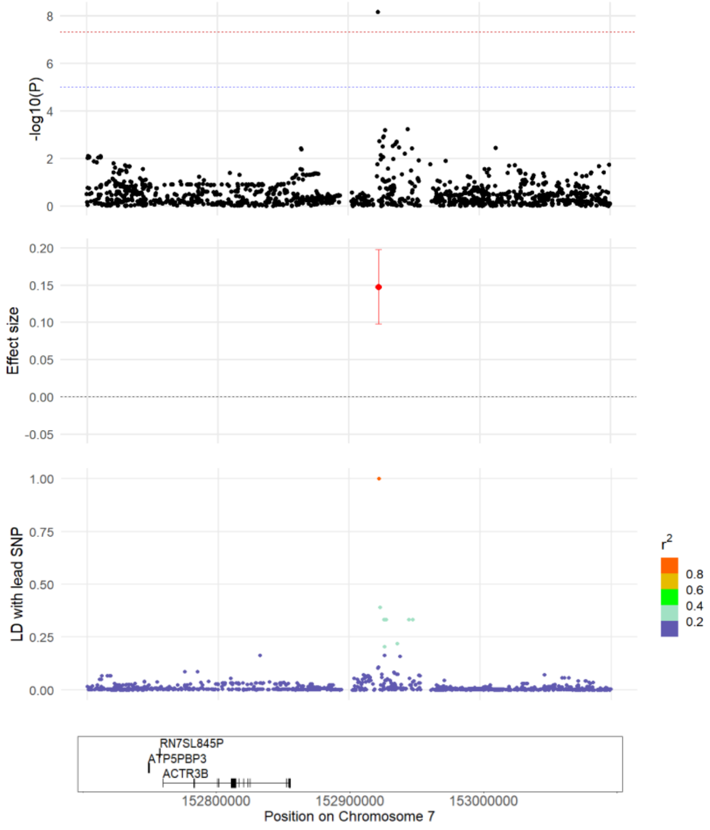

Supplementary Figure 10. Associations between sequence variants around PLPP4 and social trust in 2021, 2022 and meta-analysis. Below, the Y-axis displays the significance of the association with social trust in the form of the negative logarithm of the p-value. Above, it shows the effect size (95% confidence intervals as vertical bars) of the variants (filtered for discovery p-value <= 1e-08) on social trust. In light grey are the trust 2021 results; in dark grey, the 2022 results; and in red, the meta-analysis.

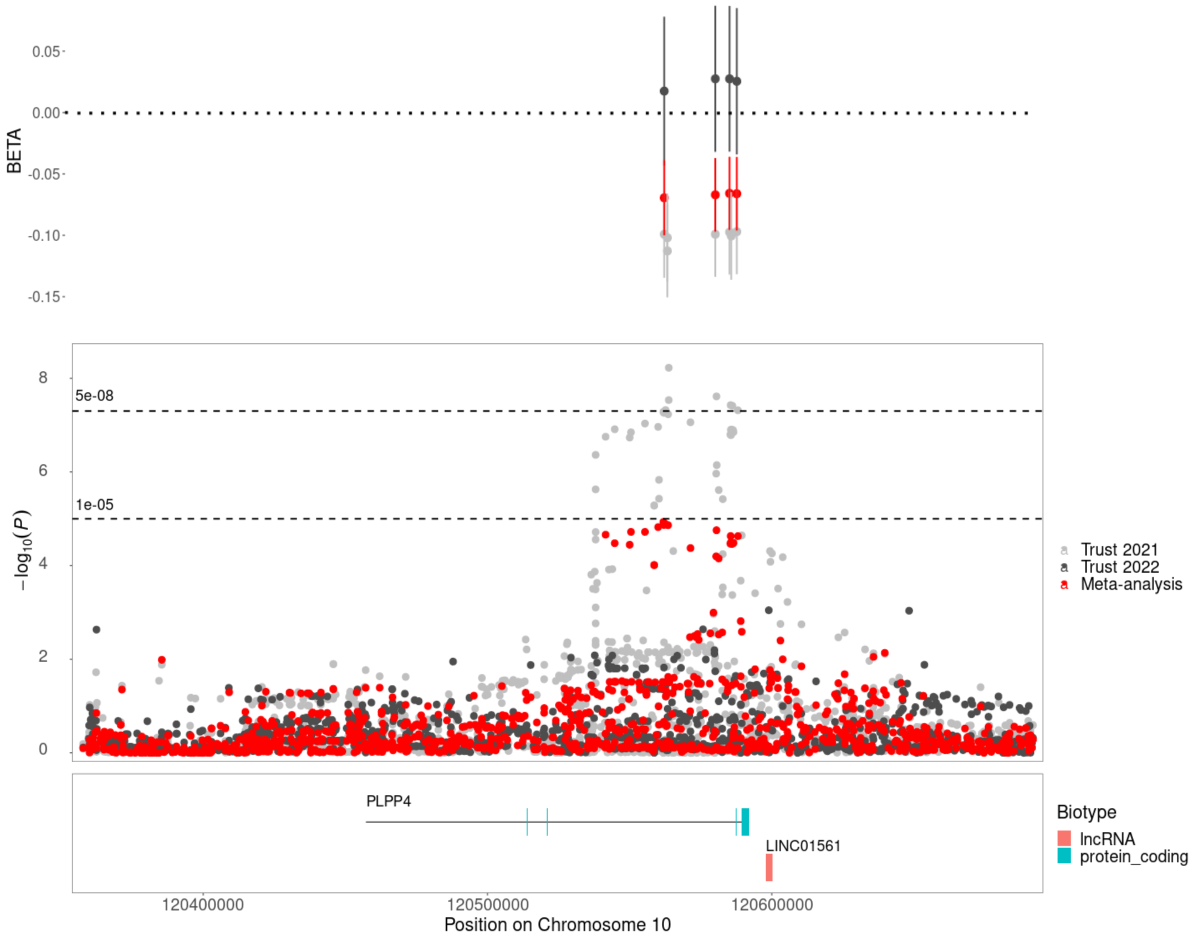

Supplementary Figure 11. Genetic correlations among social trust and 18 psychiatric and psychological traits. Blue represents positive correlations and red represents negative correlations. Correlation coefficients are unbounded. Genetic correlations that are significantly different from zero are marked with an asterisk (uncorrected) or two asterisks (Bonferroni-corrected for 190 tests). See acronyms in the Methods, TRU: social trust meta-analysis. References to the studies and sample sizes are provided in Supplementary Table 9.

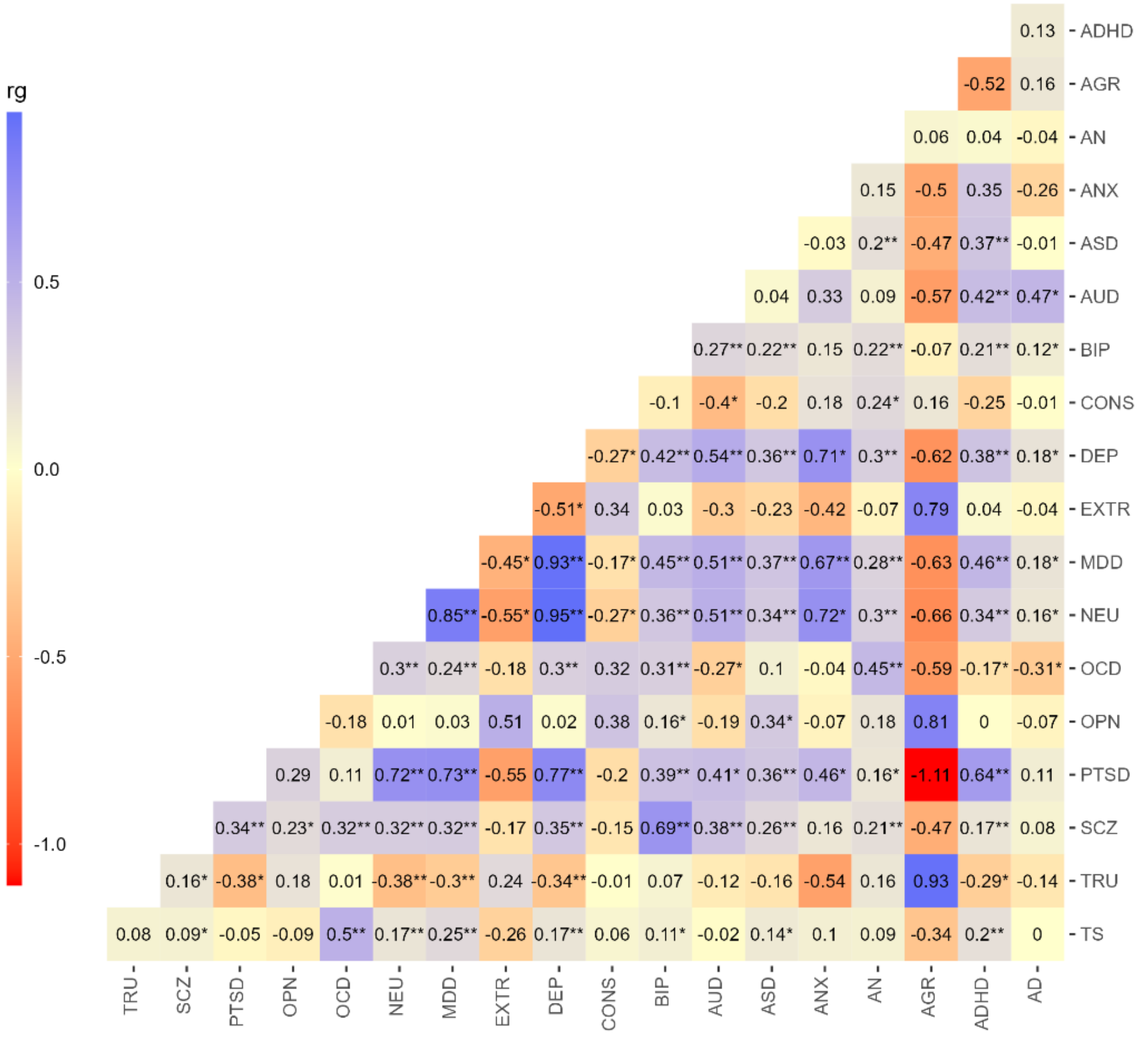

Supplementary Figure 12. Regional plots of association between social trust meta-analysis and sequence variants around the main genes previously linked to social trust. (A) Serotonin receptor gene, SLC6A4; (B) oxytocin receptor gene, OXTR; (C) CD38; (D) arginine vasopressin receptor gene, AVPR1A.

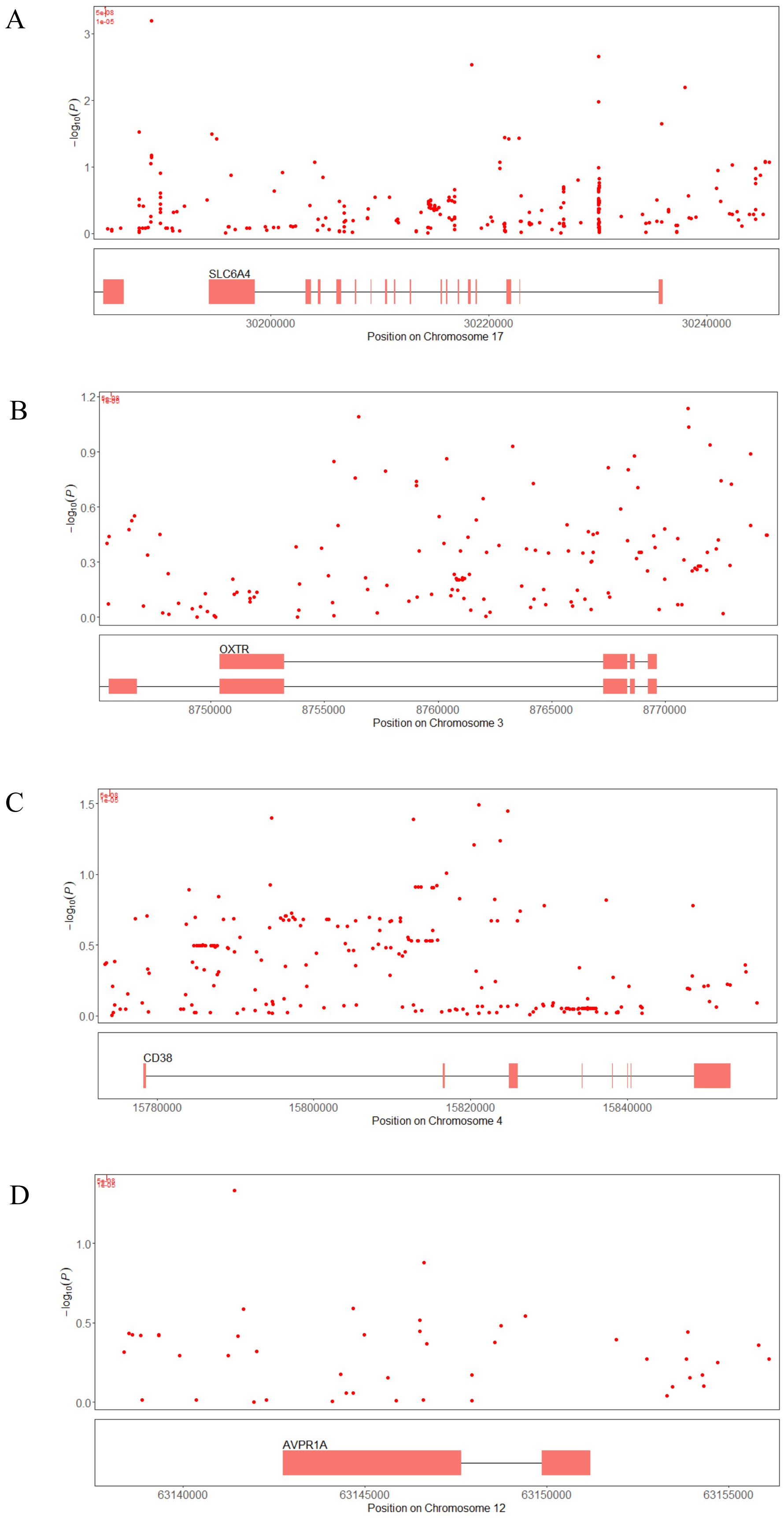

Supplementary Figure 13. Network of protein interactions including PLPP4 as obtained from the STRING database. (A) Functional and physical interactions. (B) Only physical interactions. The color of the network edges indicates the type of interaction evidence, as can be read in the legend. Annotations of these proteins, along with a tabular version of the network, can be found in the Supplementary Tables 13-14. PPAPDC1A is another name for PLPP4.

A

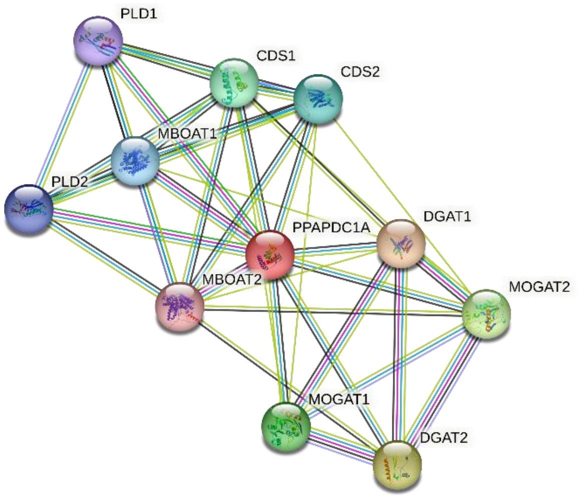

B

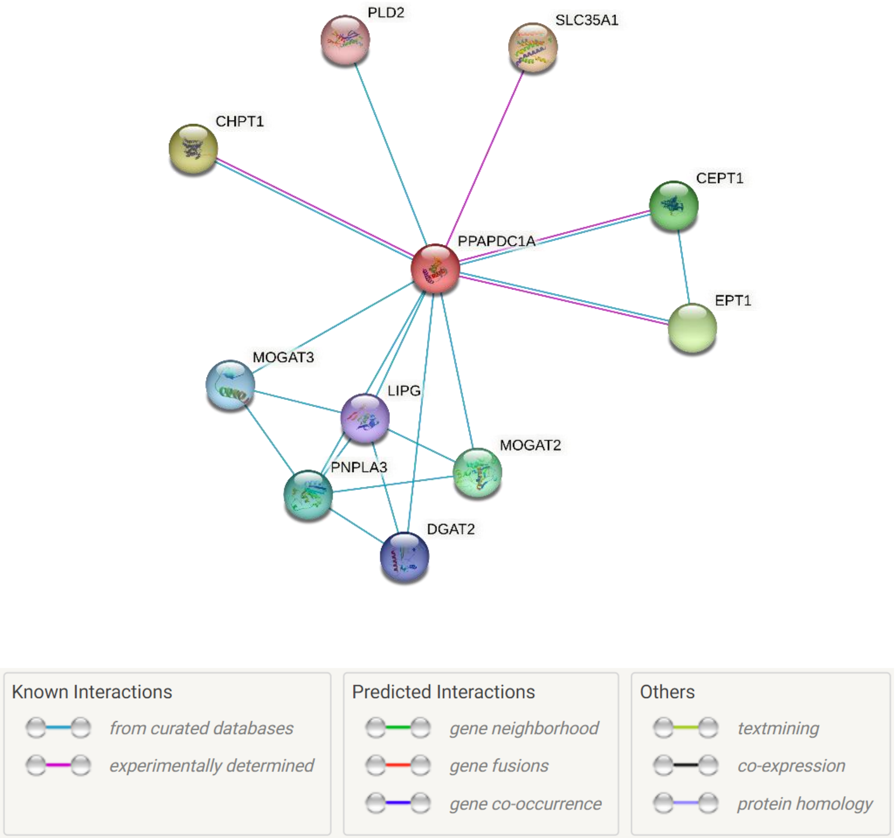

Supplementary Figure 14. Network of protein interactions including ACTR3B as obtained from the STRING database. (A) Functional and physical interactions. (B) Only physical interactions. The color of the network edges indicates the type of interaction evidence, as can be read in the legend. Annotations of these proteins, along with a tabular version of the network, can be found in the Supplementary Tables 15-16.

A

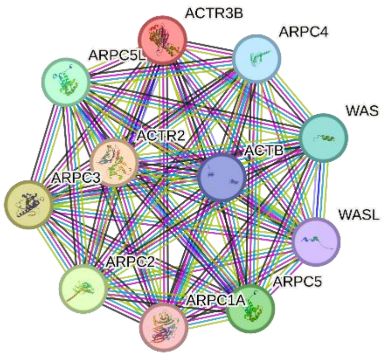

B

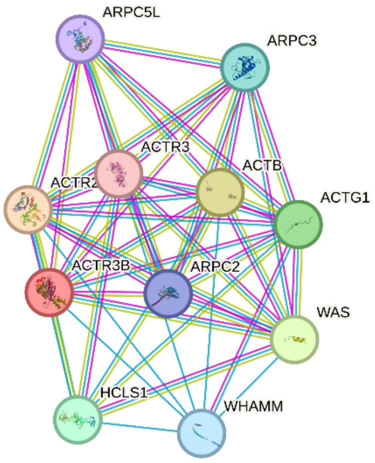

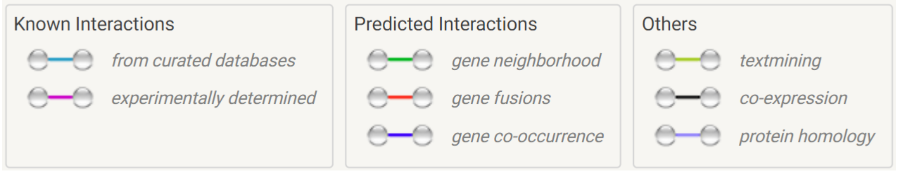

Supplement: Supplementary file 1 — Supplementary Figures. [file 41598_2024_51636_MOESM1_ESM.pdf]
