# Supplementary material for: A genome-wide association study of social trust in 33,882 Danish blood donors
Source: Sci Rep. 2024 Jan 16;14:1402. doi: 10.1038/s41598-024-51636-0 (PMC10792163; doi:10.1038/s41598-024-51636-0)
Supplement: Supplementary file 2 — Supplementary Tables. [file 41598_2024_51636_MOESM2_ESM.pdf]

A genome-wide association study of social trust in 33,882 Danish Blood Donors

Celia Burgos Sequeros[1], Thomas Folkmann Hansen[1,2], David Westergaard[1,3,4], Ioannis Louloudis[1], Sebastian Kalamajski[5], Timo Röder[1], Palle Duun Rohde[6], Michael Schwinn[7], Line Harder Clemmensen[8], Maria Didriksen[7], Mette Nyegaard[6], Henrik Hjalgrim[9,10], Kaspar René Nielsen[11], Mie Topholm Bruun[12], Sisse Rye Ostrowski[7,10], Christian Erikstrup[13,14], Susan Mikkelsen[13], Erik Sørensen[7], DBDS genomic consortium, Ole Birger Vestager Pedersen[10,11], Søren Brunak[1], Karina Banasik[*,§,1,4], Giuseppe Nick Giordano[§,5]

1. Translational Disease Systems Biology, Novo Nordisk Foundation Center for Protein Research, Faculty of Health and Medical Sciences, University of Copenhagen, Copenhagen, Denmark
2. Department of Neurology, Danish Headache Center Copenhagen University Hospital - Rigshospitalet, Glostrup, Denmark
3. Methods and Analysis, Statistics Denmark, Copenhagen, Denmark
4. Department of Gynecology and Obstetrics, Copenhagen University Hospital Hvidovre, Copenhagen, Denmark
5. Genetic and Molecular Epidemiology Unit, Lund University Diabetes Centre, Department of Clinical Sciences, CRC, Malmö, Sweden
6. Genomic Medicine, Department of Health Science and Technology, Aalborg University, Gistrup, Denmark
7. Department of Clinical Immunology, Copenhagen University Hospital - Rigshospitalet, Copenhagen, Denmark
8. Department of Applied Mathematics and Computer Science, Technical University of Denmark, Kongens Lyngby, Denmark
9. The Danish Cancer Institute; Department of Epidemiology Research, Statens Serum Institut; Department of Haematology, Rigshospitalet, Denmark
10. Department of Clinical Medicine, Faculty of Health and Medical Sciences, University of Copenhagen, Copenhagen, Denmark
11. Department of Clinical Immunology, Zealand University Hospital, Køge, Denmark
12. Clinical Immunology Research Unit, Department of Clinical Immunology, Odense University Hospital, Odense, Denmark
13. Department of Clinical Immunology, Aarhus University Hospital, Aarhus, Denmark
14. Department of Clinical Medicine, Aarhus University, Aarhus, Denmark

*Corresponding author: Karina Banasik, karina.banasik@cpr.ku.dk

§Authors contributed equally

Supplementary Tables

Supplementary Table 1. Levels of social trust measured in a random Danish sample through the European Social Survey (ESS).

Supplementary Table 2. Variant quality check of top associations with social trust.

Supplementary Table 3. Summary of MAGMA gene-level association analysis as computed by FUMA.

Supplementary Table 4. Summary of MAGMA gene set-level association analysis as computed by FUMA.

Supplementary Table 5. Summary of MAGMA tissue expression analysis as computed by FUMA.

Supplementary Table 6. Summary of the enrichment of mapped genes in gene sets as computed by FUMA.

Supplementary Table 7. External replication with Wooton at al's top sequence variants associated with social trust.

Supplementary Table 8. Studies used for computing genetic correlation with social trust.

Supplementary Table 9. Full table of PheWAS associations between discovery sequence variant, rs12776883, and phenotypes found in the GWAS ATLAS resource.

Supplementary Table 10. Full table of PheWAS associations between discovery sequence variant, rs12776883, and phenotypes in the Copenhagen Hospital Biobank Cardiovascular Disease Cohort.

Supplementary Table 11. Full table of PheWAS associations between meta-analysis sequence variant, rs71543507, and phenotypes found in the GWAS ATLAS resource.

Supplementary Table 12. Full table of PheWAS associations between meta-analysis sequence variant, rs71543507, and phenotypes in the Copenhagen Hospital Biobank Cardiovascular Disease Cohort.

Supplementary Table 13. Protein interaction network including *PLPP4* as obtained from the STRING database.

Supplementary Table 14. Protein annotations of STRING network nodes in Supplementary Table 14.

Supplementary Table 15. Protein interaction network including *ACTR3B* as obtained from the STRING database.

Supplementary Table 16. Protein annotations of STRING network nodes in Supplementary Table 17.

Supplementary Table 17. Member list of the DBDS Genomic Consortium

Supplementary Table 1. Levels of trust measured in a random Danish sample through the European Social Survey (ESS). Data was obtained from the ESS Data portal https://ess-search.nsd.no/. Questions used to measure trust were the same as those used in our study. The average of the three questions was computed for each ESS study participant and median and quartiles were calculated on those values. Note that, contrary to Figure 1 of the main text, each study participant can have responses in more than one ESS round.

year	n	median	quartiles
2002	1506	7	6, 8
2004	1487	7	5.67, 8
2006	1505	7	6, 8
2008	1610	7	6, 8
2010	1576	7	6, 7.67
2012	1650	7	6, 8
2014	1502	7	6, 7.67
2018	1572	7	6, 8

Supplementary Table 2. Variant quality check of top associations with social trust.
Parameters were calculated based on the discovery cohort.

	General				Minor allele frequencies			Genotype missingness	Hardy-Weinberg equilibrium
rsID	A1 (ref)	A2 (effect)	CHR	POS (hg19)	MAF observed	MAF gnomAD (North-Western European, non-Finnish)	MAF DanMAC5 (Danish reference)	Frequency	P-value
rs17633662	C	T	10	122322160	0.257	0.278	0.261	0.02	0.205
rs76443833	G	A	10	122323269	0.249	0.175	NA	0.031	0.352
rs12776883	C	T	10	122340062	0.271	0.298	0.256	0.006	0.094
rs17634034	T	G	10	122345048	0.275	0.301	0.275	0.004	0.094
rs11597267	C	T	10	122345647	0.249	NA	NA	0.009	0.088
rs34954484	G	C	10	122347597	0.273	0.303	0.276	0.017	0.151
rs71543507	A	G	7	152619890	0.079	0.094	0.093	0.045	0.405

Supplementary Table 3. *Summary of MAGMA gene-level association analysis as computed by FUMA. Top 200 associations are shown, sorted by ascending p-value. A total of 19067 protein coding genes were included in the analysis. Genome wide significance was defined at P = 0.05/19067 = 2.622e-6. This analysis was performed on all sequence variants and their associations with social trust in the meta-analysis.*

GENE	CHR	START	STOP	NSNPS	NPARAM	N	ZSTAT	P	SYMBOL	P Bonferroni
ENSG00000164220	5	75911307	75919259	28	9	39841	4.0724	2.33E-05	*F2RL2*	4.55E-01
ENSG00000248485	1	161228517	161255240	96	14	39841	3.9776	3.48E-05	*PCP4L1*	6.80E-01
ENSG00000145703	5	75699074	76003957	954	75	39841	3.7251	9.76E-05	*IQGAP2*	1.00E+00
ENSG00000130479	19	17830051	17845325	56	8	39841	3.6227	0.000146	*MAP1S*	1.00E+00
ENSG00000145022	3	49449639	49453908	7	3	39841	3.5033	0.00023	*TCTA*	1.00E+00
ENSG00000164062	3	49711435	49721396	6	2	39841	3.462	0.000268	*APEH*	1.00E+00
ENSG00000145029	3	49460379	49466759	7	4	39841	3.4383	0.000293	*NICN1*	1.00E+00
ENSG00000233276	3	49394609	49396033	2	1	39841	3.4343	0.000297	*GPX1*	1.00E+00
ENSG00000251655	12	11504757	11548500	135	17	39841	3.4011	0.000336	*PRB1*	1.00E+00
ENSG00000138472	3	108626639	108672742	158	21	39841	3.321	0.000449	*GUCA1C*	1.00E+00
ENSG00000069020	5	65892176	66465423	1281	77	39841	3.2996	0.000484	*MAST4*	1.00E+00
ENSG00000213995	13	111267881	111292340	124	11	39841	3.2841	0.000512	*CARKD*	1.00E+00
ENSG00000164061	3	49591922	49708978	152	15	39841	3.2375	0.000603	*BSN*	1.00E+00
ENSG00000070190	4	100737990	100791311	179	24	39841	3.2296	0.00062	*DAPP1*	1.00E+00
ENSG00000164076	3	49895421	49907655	16	4	39841	3.1885	0.000715	*CAMKV*	1.00E+00
ENSG00000152404	11	107197071	107328572	450	27	39841	3.1794	0.000738	*CWF19L2*	1.00E+00
ENSG00000025434	11	47269851	47290396	40	6	39841	3.1702	0.000762	*NR1H3*	1.00E+00
ENSG00000149534	11	59855734	59863444	20	3	39841	3.1576	0.000795	*MS4A2*	1.00E+00
ENSG00000105607	19	13001840	13025021	39	9	39841	3.1527	0.000809	*GCDH*	1.00E+00
ENSG00000177706	7	192969	300711	318	38	39841	3.1389	0.000848	*FAM20C*	1.00E+00
ENSG00000232434	9	139738867	139741797	1	1	39841	3.1376	0.000852	*C9orf172*	1.00E+00
ENSG00000187268	X	13053737	13062801	7	2	39841	3.1263	0.000885	*FAM9C*	1.00E+00
ENSG00000067560	3	49396578	49450431	93	10	39841	3.0969	0.000978	*RHOA*	1.00E+00
ENSG00000080910	1	196788898	196928356	377	19	39841	3.0955	0.000982	*CFHR2*	1.00E+00
ENSG00000120306	5	139554227	139661637	204	12	39841	3.0848	0.001019	*CYSTM1*	1.00E+00
ENSG00000173402	3	49506146	49573048	102	12	39841	3.0701	0.00107	*DAG1*	1.00E+00
ENSG00000143248	1	163080911	163291577	773	46	39841	3.0535	0.001131	*RGS5*	1.00E+00

ENSG00000173852	7	34968488	35077883	364	18	39841	3.0506	0.001142	*DPY19L1*	1.00E+00
ENSG00000076513	12	110436991	110477568	80	16	39841	3.0382	0.00119	*ANKRD13A*	1.00E+00
ENSG00000114349	3	50229045	50233949	3	1	39841	3.0265	0.001237	*GNAT1*	1.00E+00
ENSG00000126012	X	53220503	53254604	22	1	39841	3.0241	0.001247	*KDM5C*	1.00E+00
ENSG00000167822	11	55904247	55905194	5	2	39841	3.0183	0.001271	*OR8J3*	1.00E+00
ENSG00000101282	20	939095	982907	163	23	39841	3.0111	0.001302	*RSPO4*	1.00E+00
ENSG00000258572	14	95982473	95984248	10	3	39841	3.0093	0.001309	*RP11-1070N10.3*	1.00E+00
ENSG00000110514	11	47290712	47351582	133	14	39841	2.9998	0.001351	*MADD*	1.00E+00
ENSG00000139437	12	110338069	110421646	174	20	39841	2.99	0.001395	*TCHP*	1.00E+00
ENSG00000139436	12	110367607	110434194	131	17	39841	2.989	0.0014	*GIT2*	1.00E+00
ENSG00000259224	17	7384721	7386383	5	1	39841	2.9691	0.001493	*SLC35G6*	1.00E+00
ENSG00000134575	11	47260853	47270457	24	5	39841	2.9656	0.00151	*ACP2*	1.00E+00
ENSG00000183763	3	49866034	49894007	36	5	39841	2.9577	0.00155	*TRAIP*	1.00E+00
ENSG00000139329	12	91496406	91505608	38	6	39841	2.9557	0.00156	*LUM*	1.00E+00
ENSG00000131375	3	15247659	15294425	36	12	39841	2.9405	0.001638	*CAPN7*	1.00E+00
ENSG00000107518	10	116853124	117708503	1818	36	39841	2.9103	0.001806	*ATRNL1*	1.00E+00
ENSG00000134571	11	47352957	47374253	38	10	39841	2.8972	0.001882	*MYBPC3*	1.00E+00
ENSG00000113068	5	139624624	139682706	94	9	39841	2.8891	0.001932	*PFDN1*	1.00E+00
ENSG00000196440	X	100673275	100788446	200	16	39841	2.8873	0.001943	*ARMCX4*	1.00E+00
ENSG00000086548	19	42254885	42276113	41	8	39841	2.8851	0.001957	*CEACAM6*	1.00E+00
ENSG00000155970	8	16884747	16980153	280	24	39841	2.8824	0.001973	*MICU3*	1.00E+00
ENSG00000173531	3	49721380	49726934	8	3	39841	2.8807	0.001984	*MST1*	1.00E+00
ENSG00000171714	11	22214722	22304903	151	12	39841	2.8807	0.001984	*ANO5*	1.00E+00
ENSG00000170270	14	93669239	93673439	2	1	39841	2.8624	0.002102	*C14orf142*	1.00E+00
ENSG00000164077	3	49946302	49967606	22	8	39841	2.836	0.002284	*MON1A*	1.00E+00
ENSG00000179954	19	55999771	56030465	128	23	39841	2.833	0.002306	*SSC5D*	1.00E+00
ENSG00000235608	4	1396720	1400119	4	2	39841	2.8297	0.00233	*NKX1-1*	1.00E+00
ENSG00000101558	18	9913999	9960018	128	21	39841	2.8296	0.00233	*VAPA*	1.00E+00
ENSG00000164032	4	100869243	100871545	6	2	39841	2.8261	0.002356	*H2AFZ*	1.00E+00
ENSG00000007001	2	158733214	158992666	760	58	39841	2.8245	0.002368	*UPP2*	1.00E+00
ENSG00000116761	1	70876901	70905534	68	16	39841	2.8193	0.002406	*CTH*	1.00E+00
ENSG00000163704	3	9987226	9994078	11	6	39841	2.8182	0.002415	*PRRT3*	1.00E+00
ENSG00000204946	7	148959262	148994393	111	17	39841	2.8096	0.00248	*ZNF783*	1.00E+00

ENSG00000149182	11	47185848	47198676	22	7	39841	2.799	0.002563	*ARFGAP2*	1.00E+00
ENSG00000177119	12	45609770	45834187	536	21	39841	2.7987	0.002565	*ANO6*	1.00E+00
ENSG00000169241	1	155107820	155111329	3	2	39841	2.7942	0.002601	*SLC50A1*	1.00E+00
ENSG00000155158	9	15163620	15307358	470	52	39841	2.787	0.00266	*TTC39B*	1.00E+00
ENSG00000196159	4	126237554	126414087	460	37	39841	2.786	0.002668	*FAT4*	1.00E+00
ENSG00000166800	11	18477371	18501147	67	16	39841	2.7769	0.002744	*LDHAL6A*	1.00E+00
ENSG00000126822	14	65170820	65213610	89	12	39841	2.7645	0.00285	*PLEKHG3*	1.00E+00
ENSG00000181222	17	7387685	7417933	109	8	39841	2.7593	0.002897	*POLR2A*	1.00E+00
ENSG00000175906	17	41476327	41478505	4	2	39841	2.7538	0.002945	*ARL4D*	1.00E+00
ENSG00000165912	11	47199076	47207994	19	5	39841	2.7533	0.00295	*PACSIN3*	1.00E+00
ENSG00000203805	10	122216466	122349367	457	40	39841	2.743	0.003044	*PPAPDC1A*	1.00E+00
ENSG00000149573	11	118124118	118135251	30	2	39841	2.7277	0.003189	*MPZL2*	1.00E+00
ENSG00000129167	11	18039111	18063973	39	5	39841	2.7233	0.003232	*TPH1*	1.00E+00
ENSG00000214787	11	59968726	60010561	122	12	39841	2.7179	0.003285	*MS4A4E*	1.00E+00
ENSG00000165757	10	30301729	30404423	204	32	39841	2.7142	0.003322	*KIAA1462*	1.00E+00
ENSG00000242419	5	140864741	140892546	58	13	39841	2.706	0.003405	*PCDHGC4*	1.00E+00
ENSG00000170373	20	23728190	23731905	27	6	39841	2.7014	0.003452	*CST1*	1.00E+00
ENSG00000206053	16	1728257	1752281	30	5	39841	2.6965	0.003504	*HN1L*	1.00E+00
ENSG00000185271	14	20896970	20903801	25	6	39841	2.6913	0.003559	*KLHL33*	1.00E+00
ENSG00000176095	3	49761727	49823975	94	10	39841	2.6906	0.003566	*IP6K1*	1.00E+00
ENSG00000116212	1	54411750	54433841	30	8	39841	2.6891	0.003583	*LRRC42*	1.00E+00
ENSG00000149133	11	55761157	55762101	7	3	39841	2.689	0.003584	*OR5F1*	1.00E+00
ENSG00000138622	15	73612200	73661605	122	23	39841	2.686	0.003616	*HCN4*	1.00E+00
ENSG00000164532	7	35242042	35293758	129	8	39841	2.6803	0.003678	*TBX20*	1.00E+00
ENSG00000242114	22	30821518	30825045	8	4	39841	2.6779	0.003704	*MTFP1*	1.00E+00
ENSG00000182916	X	102585124	102587254	2	1	39841	2.6737	0.003751	*TCEAL7*	1.00E+00
ENSG00000181718	11	55999490	56000737	11	4	39841	2.6677	0.003818	*OR5T2*	1.00E+00
ENSG00000161860	19	13009600	13030090	35	9	39841	2.6675	0.003821	*SYCE2*	1.00E+00
ENSG00000179284	19	13075973	13085567	16	8	39841	2.6652	0.003847	*DAND5*	1.00E+00
ENSG00000049768	X	49106897	49121288	8	4	39841	2.664	0.003861	*FOXP3*	1.00E+00
ENSG00000174282	17	7362685	7387582	58	9	39841	2.6623	0.003881	*ZBTB4*	1.00E+00
ENSG00000164500	7	50135632	50199426	211	14	39841	2.6607	0.003899	*C7orf72*	1.00E+00
ENSG00000176540	11	48387037	48388017	5	2	39841	2.6534	0.003984	*OR4C5*	1.00E+00

ENSG00000129194	17	7491496	7493488	4	2	39841	2.6532	0.003987	*SOX15*	1.00E+00
ENSG00000053747	18	21269407	21535030	415	19	39841	2.6437	0.0041	*LAMA3*	1.00E+00
ENSG00000073861	17	45810610	45823485	41	10	39841	2.6417	0.004124	*TBX21*	1.00E+00
ENSG00000109270	4	100799493	100815647	25	7	39841	2.638	0.00417	*LAMTOR3*	1.00E+00
ENSG00000185684	12	132568828	132613029	96	9	39841	2.6332	0.004229	*EP400NL*	1.00E+00
ENSG00000164031	4	100817405	100867883	87	15	39841	2.6327	0.004235	*DNAJB14*	1.00E+00
ENSG00000181761	11	55889849	55890787	13	3	39841	2.6315	0.00425	*OR8H3*	1.00E+00
ENSG00000240764	5	140868808	140892546	54	12	39841	2.6293	0.004278	*PCDHGC5*	1.00E+00
ENSG00000248099	19	17927321	17932383	7	2	39841	2.6291	0.00428	*INSL3*	1.00E+00
ENSG00000174456	12	110465872	110511491	110	19	39841	2.6273	0.004304	*C12orf76*	1.00E+00
ENSG00000267901	14	71683031	71683462	1	1	39841	2.6213	0.00438	*AC004817.1*	1.00E+00
ENSG00000134389	1	196946667	196978804	56	10	39841	2.6201	0.004395	*CFHR5*	1.00E+00
ENSG00000090530	3	189674517	189840226	583	29	39841	2.6156	0.004454	*LEPREL1*	1.00E+00
ENSG00000131981	14	55590828	55612126	47	6	39841	2.6062	0.004577	*LGALS3*	1.00E+00
ENSG00000214215	12	93096619	93102325	9	4	39841	2.6042	0.004605	*C12orf74*	1.00E+00
ENSG00000130475	19	17858527	17899377	144	21	39841	2.6041	0.004606	*FCHO1*	1.00E+00
ENSG00000114850	3	156257929	156272973	52	7	39841	2.5934	0.004752	*SSR3*	1.00E+00
ENSG00000115590	2	102608306	102645006	122	9	39841	2.5926	0.004762	*IL1R2*	1.00E+00
ENSG00000177888	1	197122810	197169672	77	11	39841	2.5905	0.004792	*ZBTB41*	1.00E+00
ENSG00000213214	7	143883176	143991230	3	2	39841	2.5786	0.00496	*ARHGEF35*	1.00E+00
ENSG00000172476	X	102754678	102774417	11	5	39841	2.5723	0.005052	*RAB40A*	1.00E+00
ENSG00000102076	X	153409698	153424507	3	1	39841	2.5705	0.005078	*OPN1LW*	1.00E+00
ENSG00000149179	11	46958240	47185936	308	20	39841	2.567	0.005129	*C11orf49*	1.00E+00
ENSG00000104219	8	17013538	17082308	197	33	39841	2.5616	0.00521	*ZDHHC2*	1.00E+00
ENSG00000176198	14	20710898	20711979	3	2	39841	2.5607	0.005223	*OR11H4*	1.00E+00
ENSG00000163001	2	55746740	55773015	84	19	39841	2.5542	0.005321	*CCDC104*	1.00E+00
ENSG00000153179	12	65004293	65091347	241	23	39841	2.5492	0.005399	*RASSF3*	1.00E+00
ENSG00000139433	12	110288748	110318293	76	12	39841	2.5459	0.005449	*GLTP*	1.00E+00
ENSG00000213921	19	40267235	40276775	17	6	39841	2.5434	0.005489	*LEUTX*	1.00E+00
ENSG00000149679	20	60963688	60982341	81	12	39841	2.5426	0.005501	*CABLES2*	1.00E+00
ENSG00000181767	11	55872519	55873457	8	2	39841	2.5392	0.005556	*OR8H2*	1.00E+00
ENSG00000106665	7	73703805	73820273	270	22	39841	2.5385	0.005566	*CLIP2*	1.00E+00
ENSG00000185758	4	184242917	184243579	2	1	39841	2.5384	0.005568	*CLDN24*	1.00E+00

ENSG00000147206	X	102330738	102348157	16	7	39841	2.534	0.005639	*NXF3*	1.00E+00
ENSG00000151834	4	46250444	46477247	483	30	39841	2.5339	0.00564	*GABRA2*	1.00E+00
ENSG00000186487	2	1792885	2335032	1514	87	39841	2.5338	0.005642	*MYT1L*	1.00E+00
ENSG00000116898	1	36921319	36930038	33	5	39841	2.5318	0.005674	*MRPS15*	1.00E+00
ENSG00000261732	16	1682260	1736716	96	9	39841	2.5301	0.005701	*LA16c-431H6.6*	1.00E+00
ENSG00000177234	10	122357721	122359629	8	3	39841	2.5257	0.005773	*C10orf85*	1.00E+00
ENSG00000119457	9	115641200	115653193	32	10	39841	2.5147	0.005957	*SLC46A2*	1.00E+00
ENSG00000240184	5	140855580	140892542	80	16	39841	2.5111	0.006018	*PCDHGC3*	1.00E+00
ENSG00000188542	2	241499471	241503431	11	5	39841	2.5001	0.006208	*DUSP28*	1.00E+00
ENSG00000116885	1	36881428	36916086	143	7	39841	2.4954	0.006291	*OSCP1*	1.00E+00
ENSG00000174957	11	55944094	55945032	1	1	39841	2.4944	0.006308	*OR5J2*	1.00E+00
ENSG00000134905	13	111293759	111365950	285	18	39841	2.4879	0.006425	*CARS2*	1.00E+00
ENSG00000163945	4	1341054	1381837	142	17	39841	2.4839	0.006498	*UVSSA*	1.00E+00
ENSG00000087258	16	56225302	56391356	418	47	39841	2.4833	0.006508	*GNAO1*	1.00E+00
ENSG00000066279	1	197053258	197115824	90	8	39841	2.4814	0.006544	*ASPM*	1.00E+00
ENSG00000129255	17	7486847	7496107	12	6	39841	2.4807	0.006556	*MPDU1*	1.00E+00
ENSG00000134365	1	196819371	196888102	180	17	39841	2.4767	0.00663	*CFHR4*	1.00E+00
ENSG00000124657	6	27925019	27925960	3	1	39841	2.4766	0.006632	*OR2B6*	1.00E+00
ENSG00000168394	6	32812986	32821755	82	9	39841	2.4749	0.006663	*TAP1*	1.00E+00
ENSG00000198791	8	17086737	17104387	44	9	39841	2.4694	0.006767	*CNOT7*	1.00E+00
ENSG00000183960	3	19189946	19577138	516	50	39841	2.4689	0.006776	*KCNH8*	1.00E+00
ENSG00000100055	22	37678068	37711382	86	13	39841	2.4639	0.006871	*CYTH4*	1.00E+00
ENSG00000106603	7	43648055	43769316	360	15	39841	2.4592	0.006962	*COA1*	1.00E+00
ENSG00000172487	11	56127691	56128764	8	4	39841	2.4581	0.006984	*OR8J1*	1.00E+00
ENSG00000172037	3	49158547	49170551	10	6	39841	2.4579	0.006987	*LAMB2*	1.00E+00
ENSG00000183908	11	56949221	56959191	27	7	39841	2.453	0.007083	*LRRC55*	1.00E+00
ENSG00000182179	3	49842640	49851379	7	2	39841	2.4508	0.007128	*UBA7*	1.00E+00
ENSG00000003756	3	50126341	50156454	24	6	39841	2.4502	0.007139	*RBM5*	1.00E+00
ENSG00000174007	3	196433148	196439164	29	5	39841	2.4496	0.00715	*CEP19*	1.00E+00
ENSG00000170175	17	7348380	7361026	32	6	39841	2.4469	0.007205	*CHRNB1*	1.00E+00
ENSG00000254585	15	23888691	23891175	2	1	39841	2.4464	0.007215	*MAGEL2*	1.00E+00
ENSG00000171408	6	136172834	136516712	587	56	39841	2.4404	0.007335	*PDE7B*	1.00E+00
ENSG00000188051	19	17546318	17559376	34	8	39841	2.4357	0.007433	*TMEM221*	1.00E+00

ENSG00000010319	3	52467069	52479101	11	6	39841	2.4344	0.007458	*SEMA3G*	1.00E+00
ENSG00000104064	15	50569389	50647605	180	11	39841	2.4305	0.007539	*GABPB1*	1.00E+00
ENSG00000105875	7	134868590	134896316	81	5	39841	2.4298	0.007554	*WDR91*	1.00E+00
ENSG00000039987	19	12862516	12869272	6	4	39841	2.4293	0.007564	*BEST2*	1.00E+00
ENSG00000182187	2	209007297	209010892	15	3	39841	2.4275	0.007602	*CRYGB*	1.00E+00
ENSG00000108771	17	40253422	40264751	31	5	39841	2.4264	0.007626	*DHX58*	1.00E+00
ENSG00000144227	2	139428342	139537918	316	39	39841	2.4232	0.007693	*NXPH2*	1.00E+00
ENSG00000206561	3	15491640	15563258	219	24	39841	2.4227	0.007703	*COLQ*	1.00E+00
ENSG00000180998	14	53019866	53104431	189	23	39841	2.4223	0.007712	*GPR137C*	1.00E+00
ENSG00000142623	1	17531621	17572500	129	35	39841	2.4178	0.007808	*PADI1*	1.00E+00
ENSG00000171858	20	60962172	60963576	4	2	39841	2.4172	0.007821	*RPS21*	1.00E+00
ENSG00000162746	1	161691353	161697933	32	9	39841	2.414	0.007889	*FCRLB*	1.00E+00
ENSG00000186088	7	76940068	77045717	319	24	39841	2.414	0.00789	*GSAP*	1.00E+00
ENSG00000205821	17	2310279	2318731	32	6	39841	2.4132	0.007906	*AC006435.1*	1.00E+00
ENSG00000154065	18	21178890	21242849	92	22	39841	2.4092	0.007994	*ANKRD29*	1.00E+00
ENSG00000204388	6	31795512	31798031	3	2	39841	2.4088	0.008002	*HSPA1B*	1.00E+00
ENSG00000164068	3	49726932	49758962	49	8	39841	2.4071	0.00804	*RNF123*	1.00E+00
ENSG00000143819	1	225997794	226033260	117	31	39841	2.4071	0.00804	*EPHX1*	1.00E+00
ENSG00000164543	7	43622357	43666385	134	11	39841	2.4032	0.008127	*STK17A*	1.00E+00
ENSG00000137880	15	41056218	41059906	2	1	39841	2.3986	0.00823	*GCHFR*	1.00E+00
ENSG00000259207	17	45331212	45421658	280	25	39841	2.3961	0.008286	*ITGB3*	1.00E+00
ENSG00000007545	16	1662326	1727909	117	11	39841	2.3908	0.008405	*CRAMP1L*	1.00E+00
ENSG00000147576	8	67342420	67383836	126	17	39841	2.388	0.008471	*ADHFE1*	1.00E+00
ENSG00000143398	1	151170425	151222012	81	11	39841	2.384	0.008563	*PIP5K1A*	1.00E+00
ENSG00000135213	7	75046066	75115548	156	22	39841	2.3838	0.008568	*POM121C*	1.00E+00
ENSG00000250799	19	36290890	36304201	18	8	39841	2.3827	0.008594	*PRODH2*	1.00E+00
ENSG00000196408	16	2028918	2034193	30	6	39841	2.3824	0.0086	*NOXO1*	1.00E+00
ENSG00000056291	4	72897521	73013784	304	13	39841	2.3806	0.008642	*NPFFR2*	1.00E+00
ENSG00000268674	1	16865561	16866070	1	1	39841	2.3802	0.008652	*FAM231B*	1.00E+00
ENSG00000180425	11	114262165	114271139	20	5	39841	2.3801	0.008654	*C11orf71*	1.00E+00
ENSG00000174970	11	55734975	55735990	12	1	39841	2.379	0.008681	*OR10AG1*	1.00E+00
ENSG00000109118	17	27232268	27278789	47	11	39841	2.3763	0.008745	*PHF12*	1.00E+00
ENSG00000130363	6	159393903	159421219	167	7	39841	2.376	0.00875	*RSPH3*	1.00E+00

ENSG00000134574	11	47236493	47260767	52	8	39841	2.3757	0.008757	*DDB2*	1.00E+00
ENSG00000259753	17	45331263	45421658	279	24	39841	2.3755	0.008763	*ITGB3*	1.00E+00
ENSG00000101199	20	61904137	61921142	38	4	39841	2.3705	0.008883	*ARFGAP1*	1.00E+00
ENSG00000198877	11	55540914	55541858	2	1	39841	2.3665	0.008979	*OR5D13*	1.00E+00
ENSG00000139508	13	29274201	29293107	28	5	39841	2.3662	0.008985	*SLC46A3*	1.00E+00
ENSG00000164078	3	49924435	49941299	14	4	39841	2.365	0.009015	*MST1R*	1.00E+00
ENSG00000223510	17	14138990	14140179	1	1	39841	2.3603	0.00913	*CDRT15*	1.00E+00
ENSG00000113108	5	139937853	139973337	37	12	39841	2.3585	0.009175	*APBB3*	1.00E+00

Supplementary Table 4 *. Summary of MAGMA gene set-level association analysis as computed by FUMA. In this analysis, all sequence variants were considered. The top 200 associations are shown, sorted by ascending p-value. Variant-to-gene mapping was performed based on all the available eQTL maps (EyeGEx, eQTL catalogue, PsychENCODE, van der Wijst et al. scRNA eQTLs, DICE, eQTLGen, Blood eQTLs, MuTHER, xQTLServer, CommonMind Consortium, BRAINEAC and GTEx v8), Hi-C chromatin interaction data of 23 tissue and cell types from GEO:GSE87112, along with PsychENCODE and FANTOM datasets. A total of 10678 gene sets (curated gene sets: 4761, GO terms: 5917) from MsigDB v6.2 were included in the analysis. Genome wide significance was defined at P = 0.05/10678 = 4.68e-6.*

FULL_NAME	NGENES	BETA	BETA_STD	SE	P	P Bonferroni
GO_mf:go_ap_1_adaptor_complex_binding	5	1.2614	0.020174	0.2669	1.15E-06	0.017871998
GO_bp:go_positive_regulation_of_protein_kinase_b_signaling	165	0.26723	0.024452	0.063153	1.17E-05	0.180675162
GO_bp:go_golgi_to_endosome_transport	17	0.76003	0.022407	0.19223	3.86E-05	0.59796876
GO_mf:go_g_protein_coupled_receptor_activity	754	0.13742	0.026467	0.03625	7.53E-05	1
GO_bp:go_response_to_platelet_derived_growth_factor	21	0.65556	0.021479	0.17431	8.49E-05	1
GO_bp:go_arp2_3_complex_mediated_actin_nucleation	32	0.43752	0.01769	0.12156	0.00016	1
GO_mf:go_udp_xylosyltransferase_activity	11	0.82687	0.019613	0.23748	0.00025	1
GO_bp:go_purine_nucleoside_diphosphate_biosynthetic_process	6	1.0896	0.01909	0.31583	0.000281	1
GO_bp:go_positive_regulation_of_ossification	78	0.31687	0.019979	0.096345	0.000504	1
GO_mf:go_transmembrane_signaling_receptor_activity	1156	0.092619	0.021851	0.028254	0.000524	1
GO_mf:go_calcium_dependent_cysteine_type_endopeptidase_activity	17	0.67579	0.019924	0.20886	0.000608	1
GO_bp:go_g_protein_coupled_receptor_signaling_pathway	1236	0.087033	0.021185	0.026913	0.000612	1
Curated_gene_sets:monnier_postradiation_tumor_escape_up	391	0.12604	0.017649	0.039008	0.000618	1
GO_mf:go_ige_binding	5	1.0216	0.01634	0.31836	0.000667	1
GO_mf:go_bmp_receptor_activity	7	1.2119	0.022934	0.37977	0.00071	1
Curated_gene_sets:reactome_constitutive_signaling_by_aberrant_pi3k_in_cancer	76	0.31842	0.019819	0.1001	0.000735	1
GO_bp:go_mammary_gland_development	138	0.22544	0.018878	0.07119	0.000772	1
Curated_gene_sets:camps_colon_cancer_copy_number_dn	56	0.37265	0.01992	0.11784	0.000784	1
Curated_gene_sets:changolkar_h2afy_targets_dn	41	0.39441	0.018047	0.12488	0.000795	1
GO_mf:go_protein_xylosyltransferase_activity	5	1.0625	0.016994	0.33655	0.000798	1
GO_bp:go_regulation_of_neuromuscular_junction_development	11	0.74579	0.01769	0.23773	0.000854	1
GO_bp:go_iron_coordination_entity_transport	13	0.64049	0.016515	0.20533	0.000908	1
GO_bp:go_gland_development	432	0.12576	0.018491	0.040391	0.000926	1

Curated_gene_sets:chow_rassf1_targets_up	26	0.45582	0.016616	0.14691	0.00096	1
Curated_gene_sets:shiraishi_plzf_targets_dn	9	0.76805	0.016479	0.24782	0.000972	1
GO_bp:go_positive_regulation_of_bone_development	9	0.76521	0.016418	0.24975	0.001094	1
GO_bp:go_regulation_of_arp2_3_complex_mediated_actin_nucleation	16	0.47653	0.01363	0.15553	0.001094	1
GO_bp:go_heme_transport	9	0.73489	0.015768	0.23987	0.001095	1
GO_bp:go_venous_blood_vessel_morphogenesis	9	0.93615	0.020086	0.30574	0.001101	1
Curated_gene_sets:mcbryan_pubertal_breast_3_4wk_dn	35	0.42907	0.018143	0.1402	0.001107	1
GO_bp:go_oxalate_transport	11	0.60376	0.014321	0.19752	0.00112	1
GO_mf:go_photoreceptor_activity	14	0.66157	0.017702	0.21694	0.001148	1
GO_bp:go_actin_nucleation	48	0.3204	0.01586	0.10517	0.001159	1
Curated_gene_sets:tuomisto_tumor_suppression_by_col13a1_up	18	0.67401	0.020447	0.2225	0.001228	1
GO_bp:go_venous_blood_vessel_development	15	0.74044	0.020507	0.24489	0.001251	1
GO_bp:go_adp_biosynthetic_process	5	1.0817	0.017301	0.35821	0.001266	1
Curated_gene_sets:reactome_orexin_and_neuropeptides_ff_and_qrfp_bind_to_their_respective_receptors	7	1.1297	0.021378	0.37432	0.001274	1
GO_mf:go_heme_transporter_activity	7	0.79307	0.015007	0.26382	0.001325	1
GO_bp:go_nucleoside_diphosphate_biosynthetic_process	9	0.80538	0.01728	0.27014	0.001437	1
Curated_gene_sets:kegg_abc_transporters	45	0.3684	0.017659	0.12361	0.001442	1
GO_mf:go_molecular_transducer_activity	1420	0.074397	0.019313	0.025064	0.001499	1
GO_bp:go_organism_emergence_from_protective_structure	27	0.44312	0.01646	0.1506	0.001631	1
Curated_gene_sets:eppert_lsc_r	39	0.38518	0.017191	0.13181	0.001739	1
GO_bp:go_positive_regulation_of_osteoclast_development	5	0.91488	0.014633	0.31541	0.001865	1
GO_mf:go_vitamin_binding	134	0.20046	0.016543	0.069275	0.001906	1
GO_bp:go_negative_regulation_of_vascular_permeability	11	0.66748	0.015832	0.2314	0.001962	1
GO_bp:go_negative_regulation_of_epinephrine_secretion	5	1.0722	0.017148	0.37188	0.001972	1
GO_bp:go_actin_polymerization_dependent_cell_motility	6	1.016	0.0178	0.35375	0.002042	1
Curated_gene_sets:villanueva_liver_cancer_krt19_dn	67	0.2668	0.015595	0.09292	0.002047	1
Curated_gene_sets:reactome_rna_polymerase_iii_transcription_initiation_from_type_3_promoter	27	0.41591	0.015449	0.14486	0.002048	1
GO_bp:go_regulation_of_gonadotropin_secretion	11	0.7021	0.016653	0.24517	0.002096	1
GO_bp:go_regulation_of_luteinizing_hormone_secretion	7	0.83592	0.015818	0.29204	0.002105	1
GO_mf:go_protein_heterodimerization_activity	467	0.10909	0.016661	0.038156	0.002128	1
GO_bp:go_chaperone_mediated_autophagy	16	0.55092	0.015758	0.19387	0.002246	1

GO_bp:go_defense_response_to_gram_positive_bacterium	79	0.24765	0.015715	0.087285	0.002278	1
Curated_gene_sets:reactome_pi3k_events_in_erbb2_signaling	16	0.59257	0.016949	0.20977	0.002367	1
GO_mf:go_oxidoreductase_activity_acting_on_paired_donors_with_incorporation_or_reduction_of_molecular_oxygen	154	0.19532	0.017271	0.06918	0.002379	1
Curated_gene_sets:shin_b_cell_lymphoma_cluster_3	27	0.4433	0.016467	0.15752	0.002447	1
GO_cc:go_apical_plasma_membrane	314	0.13035	0.01639	0.046573	0.002568	1
GO_bp:go_amp_metabolic_process	8	0.75108	0.015194	0.26895	0.002616	1
GO_mf:go_arachidonic_acid_monooxygenase_activity	15	0.61341	0.016989	0.22004	0.002657	1
Curated_gene_sets:berenjeno_transformed_by_rhoa_reversibly_up	7	0.77987	0.014758	0.27979	0.00266	1
GO_bp:go_detection_of_external_biotic_stimulus	18	0.56067	0.017009	0.20127	0.002674	1
GO_bp:go_outflow_tract_septum_morphogenesis	27	0.51059	0.018966	0.18335	0.002681	1
GO_mf:go_phosphatase_inhibitor_activity	41	0.36782	0.016831	0.13215	0.002693	1
GO_bp:go_platelet_activation	153	0.18342	0.016167	0.065906	0.002695	1
GO_bp:go_regulation_of_cytolysis	17	0.61537	0.018143	0.22127	0.002712	1
Curated_gene_sets:baker_hematopoesis_stat5_targets	6	0.99316	0.0174	0.35882	0.002824	1
GO_mf:go_secondary_active_sulfate_transmembrane_transporter_activity	13	0.53288	0.01374	0.19266	0.002841	1
GO_bp:go_epoxygenase_p450_pathway	19	0.53745	0.016751	0.19433	0.002844	1
Curated_gene_sets:hu_angiogenesis_up	21	0.47432	0.015541	0.17176	0.002879	1
GO_mf:go_ferrous_iron_binding	23	0.44596	0.015291	0.16159	0.002894	1
GO_mf:go_iron_ion_binding	144	0.19379	0.016574	0.070266	0.002911	1
Curated_gene_sets:reactome_rna_polymerase_iii_transcription	40	0.32211	0.014558	0.1168	0.002913	1
Curated_gene_sets:reactome_grb2_events_in_erbb2_signaling	16	0.55467	0.015865	0.202	0.003021	1
GO_mf:go_activin_binding	14	0.70968	0.018989	0.25911	0.003085	1
GO_bp:go_positive_regulation_of_osteoblast_differentiation	55	0.30907	0.016374	0.11286	0.003088	1
GO_bp:go_lamellipodium_morphogenesis	17	0.50996	0.015035	0.1863	0.0031	1
GO_bp:go_positive_regulation_of_neutrophil_migration	26	0.44527	0.016231	0.1629	0.003137	1
GO_bp:go_gastrulation_with_mouth_forming_second	27	0.4129	0.015337	0.15117	0.003156	1
Curated_gene_sets:aung_gastric_cancer	53	0.29724	0.015459	0.10905	0.00321	1
Curated_gene_sets:schaeffer_prostate_development_and_cancer_box4_up	11	0.69675	0.016526	0.25634	0.003286	1
GO_cc:go_brush_border_membrane	51	0.30256	0.015437	0.11133	0.00329	1
GO_bp:go_interferon_gamma_production	108	0.22388	0.016598	0.082477	0.003323	1
GO_bp:go_gonadotropin_secretion	15	0.55166	0.015278	0.20354	0.003364	1
GO_mf:go_ubiquitin_binding	77	0.24462	0.015325	0.090536	0.00345	1

Curated_gene_sets:reactome_shc1_events_in_erbb2_signaling	22	0.46027	0.015435	0.1708	0.003525	1
GO_mf:go_oxalate_transmembrane_transporter_activity	10	0.56801	0.012846	0.21119	0.003581	1
GO_mf:go_manganese_ion_binding	61	0.27492	0.015336	0.10226	0.003592	1
Curated_gene_sets:reactome_the_retinoid_cycle_in_cones_daylight_vision	6	1.0565	0.01851	0.3934	0.003624	1
GO_bp:go_negative_regulation_of_hydrogen_peroxide_mediated_programmed_cell_death	5	0.97655	0.015619	0.36579	0.0038	1
GO_bp:go_positive_regulation_of_interferon_gamma_production	64	0.28332	0.016187	0.10635	0.003866	1
GO_bp:go_morphogenesis_of_an_epithelial_sheet	53	0.30374	0.015797	0.11403	0.003866	1
GO_cc:go_intrinsic_component_of_mitochondrial_outer_membrane	21	0.40962	0.013421	0.15401	0.003913	1
GO_bp:go_ectodermal_placode_development	13	0.58849	0.015174	0.22144	0.00394	1
GO_cc:go_host_intracellular_organelle	5	1.1099	0.017752	0.41781	0.003951	1
GO_bp:go_smooth_muscle_cell_chemotaxis	8	0.69057	0.01397	0.25998	0.003954	1
Curated_gene_sets:reactome_pi3k_akt_signaling_in_cancer	103	0.22088	0.015994	0.083161	0.003956	1
GO_cc:go_rna_polymerase_complex	103	0.19409	0.014054	0.073292	0.00405	1
GO_bp:go_immature_t_cell_proliferation_in_thymus	7	0.70255	0.013295	0.26555	0.00408	1
GO_mf:go_neuropeptide_receptor_activity	47	0.34868	0.01708	0.13254	0.004263	1
GO_bp:go_detection_of_biotic_stimulus	22	0.48101	0.01613	0.18292	0.004278	1
GO_bp:go_positive_regulation_of_gonadotropin_secretion	7	0.76623	0.0145	0.29167	0.004311	1
Curated_gene_sets:wong_endmetrium_cancer_dn	75	0.2629	0.016256	0.10037	0.004409	1
GO_bp:go_pigmentation	96	0.2195	0.015347	0.083936	0.004464	1
Curated_gene_sets:shen_smarca2_targets_dn	289	0.12173	0.014694	0.046578	0.004485	1
Curated_gene_sets:turjanski_mapk11_targets	5	0.8519	0.013625	0.32607	0.004496	1
GO_bp:go_positive_regulation_of_digestive_system_process	10	0.71518	0.016175	0.27469	0.004617	1
GO_bp:go_cd4_positive_alpha_beta_t_cell_lineage_commitment	17	0.56588	0.016683	0.21736	0.004619	1
Curated_gene_sets:pid_alk1_pathway	25	0.44108	0.015767	0.16946	0.004627	1
GO_cc:go_centriole	133	0.1784	0.014668	0.068561	0.004637	1
GO_bp:go_follicle_stimulating_hormone_secretion	7	0.76596	0.014494	0.29473	0.00468	1
GO_mf:go_oxo_acid_lyase_activity	6	0.71697	0.012561	0.27591	0.004685	1
GO_bp:go_regulation_of_protein_complex_stability	10	0.69635	0.015749	0.26808	0.004699	1
GO_bp:go_aromatic_amino_acid_family_metabolic_process	12	0.65137	0.016137	0.25096	0.004726	1
GO_bp:go_thrombin_activated_receptor_signaling_pathway	12	0.59435	0.014724	0.22936	0.004784	1
GO_bp:go_amino_sugar_catabolic_process	8	0.84456	0.017085	0.32602	0.004796	1

GO_bp:go_positive_regulation_of_membrane_protein_ectodomain_proteolysis	15	0.50573	0.014006	0.19561	0.004867	1
GO_bp:go_negative_regulation_of_interleukin_2_biosynthetic_process	5	1.0296	0.016468	0.39908	0.004945	1
GO_mf:go_n6_methyladenosine_containing_rna_binding	7	0.68489	0.01296	0.26723	0.005194	1
GO_bp:go_epinephrine_transport	8	0.79213	0.016024	0.31043	0.005364	1
GO_bp:go_inflammatory_response	681	0.084512	0.015499	0.033133	0.00538	1
GO_bp:go_endoplasmic_reticulum_tubular_network_organization	16	0.44536	0.012739	0.17467	0.005394	1
GO_bp:go_negative_regulation_of_t_cell_cytokine_production	9	0.65768	0.014111	0.25826	0.005443	1
Curated_gene_sets:iwanaga_e2f1_targets_induced_by_serum	24	0.48391	0.016949	0.19027	0.005494	1
GO_bp:go_negative_regulation_of_mesenchymal_cell_proliferation	8	0.86963	0.017592	0.34251	0.005563	1
GO_cc:go_apical_part_of_cell	378	0.10778	0.014844	0.042469	0.005583	1
GO_bp:go_wound_healing	527	0.091869	0.014882	0.036209	0.005591	1
GO_bp:go_midgut_development	13	0.6641	0.017123	0.26202	0.005634	1
Curated_gene_sets:jazag_tgfb1_signaling_dn	33	0.32768	0.013454	0.12941	0.005674	1
GO_mf:go_odorant_binding	81	0.27898	0.017924	0.11024	0.005698	1
GO_bp:go_rho_protein_signal_transduction	201	0.14756	0.014888	0.058395	0.005757	1
GO_bp:go_phototransduction	57	0.27294	0.01472	0.10818	0.005821	1
GO_bp:go_branching_involved_in_blood_vessel_morphogenesis	29	0.39395	0.015165	0.15632	0.00587	1
Curated_gene_sets:reactome_negative_regulation_of_the_pi3k_akt_network	111	0.20221	0.015197	0.080338	0.005923	1
Curated_gene_sets:biocarta_alk_pathway	35	0.35423	0.014978	0.14086	0.005961	1
Curated_gene_sets:reactome_met_activates_pi3k_akt_signaling	6	0.88176	0.015448	0.35072	0.005971	1
GO_bp:go_cellular_response_to_lead_ion	5	0.97208	0.015547	0.38677	0.005984	1
GO_bp:go_detection_of_other_organism	13	0.61147	0.015766	0.24396	0.006103	1
Curated_gene_sets:wallace_prostate_cancer_race_dn	82	0.21645	0.013992	0.086484	0.006165	1
Curated_gene_sets:reactome_opsins	8	0.68283	0.013813	0.27441	0.006421	1
GO_bp:go_positive_regulation_of_myoblast_proliferation	8	0.72022	0.01457	0.28952	0.006435	1
Curated_gene_sets:reactome_rna_polymerase_iii_transcription_termination	22	0.42068	0.014107	0.16936	0.006501	1
GO_bp:go_regulation_of_histone_deacetylation	24	0.39127	0.013704	0.15781	0.006586	1
GO_bp:go_exocrine_system_development	48	0.3178	0.015732	0.12818	0.006587	1
GO_mf:go_mannosyl_oligosaccharide_mannosidase_activity	9	0.59928	0.012858	0.24213	0.006666	1
GO_bp:go_gland_morphogenesis	118	0.19883	0.015404	0.080455	0.006736	1
GO_bp:go_icosanoid_metabolic_process	78	0.22886	0.01443	0.092852	0.00686	1
GO_bp:go_alpha_beta_t_cell_lineage_commitment	19	0.50734	0.015812	0.20605	0.006909	1

Curated_gene_sets:tomlins_metastasis_dn	20	0.4062	0.012989	0.16501	0.006919	1
GO_bp:go_positive_regulation_of_respiratory_burst	6	0.81848	0.01434	0.33253	0.006924	1
GO_bp:go_regulation_of_receptor_recycling	23	0.37343	0.012804	0.15204	0.007027	1
GO_bp:go_response_to_bacterial_lipoprotein	7	0.86256	0.016322	0.35123	0.007033	1
Curated_gene_sets:ruan_response_to_tnf_up	12	0.602	0.014914	0.24654	0.007312	1
Curated_gene_sets:lu_tumor_vasculature_dn	13	0.66871	0.017242	0.27391	0.007321	1
GO_mf:go_rna_directed_dna_polymerase_activity	6	0.72913	0.012774	0.29876	0.007338	1
GO_mf:go_telomerase_activity	6	0.72913	0.012774	0.29876	0.007338	1
GO_bp:go_outflow_tract_morphogenesis	75	0.25232	0.015602	0.10353	0.007405	1
Curated_gene_sets:reactome_erbb2_activates_ptk6_signaling	13	0.52438	0.013521	0.21547	0.007478	1
GO_bp:go_nls_bearing_protein_import_into_nucleus	21	0.40965	0.013422	0.16848	0.007524	1
GO_bp:go_negative_regulation_of_creb_transcription_factor_activity	5	0.94613	0.015132	0.39008	0.007649	1
GO_bp:go_positive_regulation_of_cytolysis	11	0.6894	0.016352	0.28476	0.007744	1
Curated_gene_sets:lee_bmp2_targets_up	745	0.073644	0.014103	0.030431	0.007764	1
GO_cc:go_intrinsic_component_of_mitochondrial_membrane	71	0.22064	0.013275	0.091339	0.00786	1
Curated_gene_sets:finetti_breast_cancers_kinome_blue	21	0.42087	0.01379	0.17423	0.007861	1
GO_bp:go_lymphangiogenesis	15	0.5588	0.015476	0.23173	0.00795	1
GO_bp:go_regulation_of_epithelial_to_mesenchymal_transition_involved_in_endocardial_cushion_formation	5	1.0069	0.016104	0.41767	0.007967	1
GO_bp:go_positive_regulation_of_epithelial_to_mesenchymal_transition_involved_in_endocardial_cushion_formation	5	1.0069	0.016104	0.41767	0.007967	1
Curated_gene_sets:reactome_fceri_mediated_caplus2_mobilization	31	0.35743	0.014225	0.14828	0.007971	1
GO_bp:go_modulation_by_symbiont_of_host_cellular_process	27	0.36729	0.013643	0.1524	0.007982	1
GO_bp:go_mammary_gland_formation	7	0.82855	0.015679	0.34532	0.008217	1
Curated_gene_sets:stark_prefrontal_cortex_22q11_deletion_dn	502	0.082635	0.013074	0.034458	0.008244	1
GO_bp:go_myelin_assembly	19	0.43077	0.013426	0.17969	0.008262	1
GO_bp:go_regulation_of_cellular_response_to_hypoxia	13	0.45665	0.011774	0.19049	0.008264	1
Curated_gene_sets:landis_erbb2_breast_tumors_324_up	145	0.16499	0.014159	0.068831	0.008271	1
GO_bp:go_regulation_of_actin_nucleation	28	0.30694	0.01161	0.12807	0.00828	1
Curated_gene_sets:reactome_activation_of_the_phototransduction_cascade	11	0.53249	0.01263	0.22238	0.008327	1
GO_bp:go_detection_of_stimulus	598	0.097903	0.016863	0.040906	0.008352	1
GO_bp:go_cellular_transition_metal_ion_homeostasis	107	0.18236	0.013457	0.076262	0.008402	1
GO_mf:go_c3hc4_type_ring_finger_domain_binding	6	0.7836	0.013729	0.32779	0.008416	1

GO_bp:go_sensory_perception_of_smell	371	0.14342	0.019573	0.059998	0.00842	1
Curated_gene_sets:reactome_signaling_by_fgfr3_fusions_in_cancer	10	0.55407	0.012531	0.23196	0.008461	1
Curated_gene_sets:bandres_response_to_carmustin_mgmt_48hr_dn	155	0.15138	0.013429	0.063464	0.008537	1
GO_bp:go_protein_chromophore_linkage	11	0.56235	0.013339	0.23678	0.00878	1
Curated_gene_sets:reactome_pi3k_events_in_erbb4_signaling	10	0.69214	0.015653	0.29149	0.008792	1
Curated_gene_sets:gentles_leukemic_stem_cell_dn	19	0.42084	0.013116	0.17769	0.008937	1
Curated_gene_sets:sesto_response_to_uv_c3	21	0.41609	0.013633	0.17572	0.00895	1
GO_bp:go_activin_receptor_signaling_pathway	39	0.33582	0.014987	0.1419	0.008983	1
GO_bp:go_receptor_transactivation	5	0.71173	0.011383	0.301	0.009033	1
Curated_gene_sets:pid_erbb_network_pathway	15	0.53384	0.014785	0.22598	0.009085	1
GO_bp:go_regulation_of_chaperone_mediated_autophagy	8	0.54608	0.011047	0.23134	0.009129	1
Curated_gene_sets:reactome_olfactory_signaling_pathway	355	0.14347	0.019161	0.060833	0.009182	1
GO_mf:go_mediator_complex_binding	5	0.77573	0.012407	0.32899	0.009195	1
GO_bp:go_oxidative_dna_demethylation	5	0.75048	0.012003	0.31833	0.009203	1
GO_bp:go_positive_regulation_of_cytokine_production_involved_in_inflammatory_response	15	0.4958	0.013731	0.21051	0.00926	1
GO_bp:go_norepinephrine_metabolic_process	6	0.85084	0.014907	0.36136	0.009277	1
GO_bp:go_associative_learning	77	0.2219	0.013902	0.09437	0.009357	1
GO_mf:go_histone_methyltransferase_activity_h3_k27_specific_	5	0.80121	0.012814	0.34129	0.009453	1
GO_mf:go_delayed_rectifier_potassium_channel_activity	32	0.37989	0.015361	0.16189	0.009478	1
GO_bp:go_antibiotic_metabolic_process	88	0.21191	0.014188	0.090313	0.009484	1

Supplementary Table 5 *. Results of MAGMA tissue expression analysis as computed by FUMA. Tissues are sorted by ascending p-value of association. This analysis was performed on all sequence variants and their associations with social trust in the meta-analysis.*

GTEx v8 53 tissue types

FULL_NAME	TYPE	NGENES	BETA	BETA_STD	SE	P
Colon_Sigmoid	COVAR	17864	0.016814	0.032901	0.011795	0.077007
Liver	COVAR	17864	0.00831	0.014948	0.005988	0.082605
Bladder	COVAR	17864	0.015347	0.029911	0.012323	0.1065
Nerve_Tibial	COVAR	17864	0.01077	0.021674	0.009456	0.12737
Esophagus_Mucosa	COVAR	17864	0.007921	0.015315	0.007168	0.13459
Skin_Sun_Exposed_Lower_leg	COVAR	17864	0.006475	0.012566	0.0073	0.18752
Colon_Transverse	COVAR	17864	0.00924	0.016992	0.010951	0.1994
Testis	COVAR	17864	0.004273	0.0073667	0.00521	0.20606
Skin_Not_Sun_Exposed_Suprapubic	COVAR	17864	0.005858	0.0113	0.007272	0.21025
Esophagus_Muscularis	COVAR	17864	0.006968	0.01374	0.011704	0.27581
Vagina	COVAR	17864	0.005931	0.011343	0.010392	0.28409
Artery_Tibial	COVAR	17864	0.005012	0.010311	0.009424	0.29743
Stomach	COVAR	17864	0.005702	0.010357	0.011248	0.30609
Brain_Cortex	COVAR	17864	0.00314	0.0057279	0.006684	0.31928
Cervix_Ectocervix	COVAR	17864	0.005118	0.010029	0.011404	0.32681
Brain_Putamen_basal_ganglia	COVAR	17864	0.003287	0.0055931	0.007463	0.32983
Brain_Anterior_cingulate_cortex_BA24	COVAR	17864	0.002292	0.0040499	0.006775	0.36758
Brain_Nucleus_accumbens_basal_ganglia	COVAR	17864	0.002264	0.00393	0.007128	0.37537
Brain_Substantia_nigra	COVAR	17864	0.002529	0.0043552	0.008091	0.37731
Brain_Frontal_Cortex_BA9	COVAR	17864	0.001961	0.0036156	0.006453	0.38059
Adipose_Subcutaneous	COVAR	17864	0.002874	0.0057346	0.010029	0.38723
Brain_Amygdala	COVAR	17864	0.001518	0.0025862	0.00736	0.41832
Uterus	COVAR	17864	0.001846	0.0037577	0.01026	0.42862
Brain_Cerebellum	COVAR	17864	0.000772	0.0015341	0.005885	0.4478
Brain_Hippocampus	COVAR	17864	0.000677	0.0011465	0.007484	0.46397
Brain_Spinal_cord_cervical_c-1	COVAR	17864	0.000296	0.0005323	0.008126	0.48547

Brain_Cerebellar_Hemisphere	COVAR	17864	2.18E-05	4.38E-05	0.005713	0.49848
Brain_Caudate_basal_ganglia	COVAR	17864	-0.00013	-0.00022	0.0074	0.50685
Adrenal_Gland	COVAR	17864	-0.00043	-0.000828	0.009369	0.51825

GTEx v8 30 general tissue types

VARIABLE	TYPE	NGENES	BETA	BETA_STD	SE	P
Bladder	COVAR	17864	0.026504	0.051657	0.015227	0.040892
Colon	COVAR	17864	0.023999	0.044933	0.014959	0.054338
Liver	COVAR	17864	0.00935	0.01682	0.006191	0.065505
Esophagus	COVAR	17864	0.022444	0.04228	0.016235	0.083429
Nerve	COVAR	17864	0.012269	0.024691	0.009776	0.10476
Testis	COVAR	17864	0.004395	0.0075766	0.005219	0.19983
Vagina	COVAR	17864	0.009831	0.018803	0.011992	0.20617
Skin	COVAR	17864	0.007263	0.013859	0.00949	0.22204
Stomach	COVAR	17864	0.009284	0.016861	0.01279	0.23398
Uterus	COVAR	17864	0.003668	0.0074683	0.011304	0.37279
Cervix_Uteri	COVAR	17864	0.004099	0.0080512	0.013413	0.37996
Blood_Vessel	COVAR	17864	0.001801	0.0036521	0.011096	0.43552
Adrenal_Gland	COVAR	17864	0.000265	0.0005118	0.009669	0.48907
Prostate	COVAR	17864	9.65E-05	0.000183	0.013154	0.49707
Brain	COVAR	17864	-0.00019	-0.000337	0.006453	0.51198
Breast	COVAR	17864	-0.00056	-0.001075	0.014966	0.5149
Small_Intestine	COVAR	17864	-0.00066	-0.001215	0.010251	0.52569
Adipose_Tissue	COVAR	17864	-0.0009	-0.001772	0.012569	0.52862
Blood	COVAR	17864	-0.00068	-0.001227	0.00582	0.54669
Fallopian_Tube	COVAR	17864	-0.00161	-0.003113	0.013122	0.54868
Ovary	COVAR	17864	-0.00245	-0.004969	0.009731	0.5995
Spleen	COVAR	17864	-0.00203	-0.004033	0.007405	0.60822
Muscle	COVAR	17864	-0.00257	-0.004861	0.006453	0.65488
Kidney	COVAR	17864	-0.00601	-0.010579	0.008967	0.74864
Salivary_Gland	COVAR	17864	-0.00854	-0.015897	0.010268	0.79726
Lung	COVAR	17864	-0.01	-0.019359	0.00995	0.84263

Pancreas	COVAR	17864	-0.00998	-0.016695	0.00827	0.88634
Pituitary	COVAR	17864	-0.01139	-0.02126	0.007779	0.92841
Heart	COVAR	17864	-0.01404	-0.024143	0.008933	0.94198

Brainspan 11 general developmental stages of brain samples

VARIABLE	TYPE	NGENES	BETA	BETA_STD	SE	P
Middle_adulthood	COVAR	14071	0.010785	0.016056	0.014798	0.23306
Late_mid-prenatal	COVAR	14071	0.007336	0.010892	0.01321	0.28935
Late_childhood	COVAR	14071	0.006594	0.0097282	0.017836	0.3558
Early_prenatal	COVAR	14071	0.002659	0.0040113	0.009253	0.38693
Late_infancy	COVAR	14071	0.002776	0.0040556	0.019685	0.44393
Early_mid-prenatal	COVAR	14071	0.001475	0.0022195	0.010624	0.4448
Early_infancy	COVAR	14071	-0.00135	-0.00191	0.027377	0.5196
Adolescence	COVAR	14071	-0.00484	-0.006843	0.019116	0.6
Early_childhood	COVAR	14071	-0.00407	-0.005695	0.013642	0.61731
Young_adulthood	COVAR	14071	-0.01267	-0.018085	0.017446	0.76616
Late_prenatal	COVAR	14071	-0.01972	-0.027141	0.015252	0.90193

Brainspan 29 different ages of brain samples

VARIABLE	TYPE	NGENES	BETA	BETA_STD	SE	P
10_mos	COVAR	14535	0.000915	0.027962	0.000627	0.072384
19_pcw	COVAR	14535	0.000448	0.014343	0.000371	0.11359
8_yrs	COVAR	14535	0.000689	0.020605	0.000674	0.15328
4_mos	COVAR	14535	0.001015	0.027474	0.001012	0.15809
17_pcw	COVAR	1.45E+04	0.000268	0.0068709	0.000533	0.3075
4_yrs	COVAR	14535	0.000168	0.00585	0.00043	0.34782
21_pcw	COVAR	14535	0.000103	0.0026461	0.000562	0.42729
24_pcw	COVAR	14535	4.63E-05	0.0013754	0.000487	0.46216
16_pcw	COVAR	14535	-3.13E-05	-0.000823	0.000483	0.52581
18_yrs	COVAR	14535	-0.00019	-0.004104	0.00071	0.60504
12_pcw	COVAR	14535	-0.00017	-0.004703	0.000407	0.65965

37_pcw	COVAR	14535	-0.00022	-0.006852	0.000506	0.67018
40_yrs	COVAR	14535	-0.0003	-0.008763	0.000604	0.69121
13_pcw	COVAR	14535	-0.0002	-0.005713	0.000387	0.69291
26_pcw	COVAR	1.45E+04	-0.00045	-0.009449	0.000834	0.70331
8_pcw	COVAR	14535	-0.00019	-0.00553	0.000343	0.70755
13_yrs	COVAR	14535	-0.00056	-0.012834	0.000803	0.7553
11_yrs	COVAR	14535	-0.0005	-0.0135	0.000681	0.77081
2_yrs	COVAR	14535	-0.00039	-0.008247	0.000517	0.77243
1_yrs	COVAR	14535	-0.00066	-0.015439	0.000738	0.81279
9_pcw	COVAR	14535	-0.0004	-0.009687	0.000425	0.82588
30_yrs	COVAR	14535	-0.00052	-0.015361	0.000527	0.83872
19_yrs	COVAR	14535	-0.00073	-0.0191	0.000633	0.87591
36_yrs	COVAR	14535	-0.00105	-0.022512	0.000807	0.90239
37_yrs	COVAR	14535	-0.00101	-0.022282	0.000775	0.90443
3_yrs	COVAR	14535	-0.00082	-0.020719	0.000598	0.9137
21_yrs	COVAR	14535	-0.00102	-0.024448	0.000708	0.92592
15_yrs	COVAR	14535	-0.00111	-0.02641	0.000725	0.93759
23_yrs	COVAR	14535	-0.00139	-0.031366	0.000838	0.9513

Supplementary Table 6. Summary of the enrichment of mapped genes in gene sets as computed by FUMA. In this analysis, only sequence variants with association p-value < 1e-05 in the meta-analysis were considered. Variant-to-gene mapping was performed based on all the available eQTL maps (EyeGEx, eQTL catalogue, PsychENCODE, van der Wijst et al. scRNA eQTLs, DICE, eQTLGen, Blood eQTLs, MuTHER, xQTLServer, CommonMind Consortium, BRAINEAC and GTEx v8), Hi-C chromatin interaction data of 23 tissue and cell types from GEO:GSE87112, along with PsychENCODE and FANTOM datasets. Results are sorted by ascending p-value. Gene sets from MSigDB, WikiPathways and reported genes from the GWAS catalog were included in the analysis. Multiple test correction was performed per category.

**Category**	**GeneSet**	**N_genes**	**N_overlap**	**p-value**	**adjP**	**genes**	**link**
Positional_gene_sets	chr1q23	193	65	5.79E-66	1.73E-63	*HDGF:FCRL5:FCRL4:FCRL3:VDAC1P9:FCRL2:FCRL1:MRPS21P2:RP11-404O13.1:ELL2P1:CD1D:OR10T2:OR10K2:OR10R2:OR10R3P:OR10R1P:OR6Y1:OR6P1:OR10X1:OR10Z1:SPTA1:OR6K1P:OR6K2:OR6K3:OR6K4P:OR6K5P:OR6K6:OR6N1:OR6N2:OR2AQ1P:OR10AA1P:MNDA:PYHIN1:IFI16:AIM2:RAD1P2:CADM3:RNA5SP60:DARC:MPTX1:OR10J2P:FCER1A:OR10J3:OR10J7P:OR10J8P:OR10J4:OR10J1:OR10J5:OR10J6P:CRPP1:CRP:DUSP23:C1orf204:RP11-190A12.7:CCDC19:hsa-mir-4259:SLAMF9:LINC01133:SLAMF6:CD84:RNA5SP62:RNA5SP63:NMNAT1P2:PBX1:LMX1A:RXRG*	http://www.gsea-msigdb.org/gsea/msigdb/cards/chr1q23

Positional_gene_sets	chr11q12	309	67	8.99E-54	1.34E-51	*OR5W2:OR5I1:OR10AF1P:OR5F1:OR5F2P:OR5AS1:OR5AQ1P:OR5J1P:OR5BE1P:OR8I2:OR8I4P:OR8H2:OR8K5:OR5J7P:OR5J2:OR8V1P:OR8J2:OR8K3:FAM8A2P:OR8K2P:OR8K1:OR5M6P:OR5M5P:OR5M11:OR5M10:OR5M13P:OR5M1:OR5AM1P:OR5M12P:OR5G5P:OR5G3:AP000479.1:OR5AK3P:OR5AK2:OR5AK1P:OR5BQ1P:OR5AK4P:OR5AO1P:OR5BP1P:LRRC55:APLNR:TNKS1BP1:PRG2:SLC43A3:TIMM10:SERPING1:OR5BC1P:OR5B19P:OR5B10P:CNTF:GLYAT:GLYATL2:GLYATL1P2:GLYATL1:GLYATL1P3:FAM111B:MPEG1:RN7SL42P:SLC25A47P1:OR5A1:OR4D6:OR4D10:OR4D8P:OR4D11:GIF:TCN1:SRD5A3P1*	http://www.gsea-msigdb.org/gsea/msigdb/cards/chr11q12
Positional_gene_sets	chr9p24	71	37	1.09E-46	1.09E-44	*RNU2-25P:RN7SL592P:RP11-125B21.2:KCNV2:KIAA0020:CARM1P1:RFX3:RP11-509J21.1:GLIS3:GLIS3-AS1:RNU6-694P:SLC1A1:SPATA6L:PPAPDC2:RP11-6J24.6:CDC37L1:AK3:MIR101-2:HNRNPA1P41:JAK2:MTND6P5:MTND1P11:MTND4P14:MTND5P14:IGHEP2:INSL6:INSL4:RLN2:HMGN2P31:RLN1:PLGRKT:CD274:PDCD1LG2:KIAA1432:ERMP1:MLANA:IL33*	http://www.gsea-msigdb.org/gsea/msigdb/cards/chr9p24

Positional_gene_sets	chr6p24	52	33	7.79E-46	5.83E-44	*CAGE1:RIOK1:SNRNP48:BMP6:BLOC1S5-TXNDC5:PIP5K1P1:BLOC1S5:EEF1E1-BLOC1S5:EEF1E1:SLC35B3:HULC:OFCC1:RNU6ATAC21P:TFAP2A:TFAP2A-AS1:LINC00518:RP1-290I10.7:MIR5689:MRPL48P1:GCNT2:GCNT6:C6orf52:PAK1IP1:TMEM14C:TMEM14B:SYCP2L:RNA5SP203:MAK:GCM2:ELOVL2:ELOVL2-AS1:SMIM13:ERVFRD-1*	http://www.gsea-msigdb.org/gsea/msigdb/cards/chr6p24
Positional_gene_sets	chr17q24	79	28	1.09E-29	6.53E-28	*FAM20A:RP11-118B18.1:AC011591.1:ABCA8:ABCA9:ABCA9-AS1:ABCA6:MIR4524B:ABCA10:ABCA5:SNRPGP4:MAP2K6:AC003051.1:AC004562.1:LINC01028:KCNJ16:KCNJ2-AS1:KCNJ2:CALM2P1:CASC17:RNU7-155P:AC005152.3:LINC01152:SOX9-AS1:AC007461.2:SOX9:LINC00511:RPL32P33*	http://www.gsea-msigdb.org/gsea/msigdb/cards/chr17q24
Positional_gene_sets	chr1q24	106	22	5.81E-18	2.90E-16	*RP11-280O1.2:LRRC52:MGST3:ALDH9A1:TMCO1:UCK2:FAM78B:MIR921:FMO7P:RP11-276E17.2:FMO8P:FMO9P:FMO10P:RPL4P2:CNN2P10:POGK:DUTP6:GPA33:DUSP27:CD247:AKR1D1P1:CREG1*	http://www.gsea-msigdb.org/gsea/msigdb/cards/chr1q24

GO_mf	GO_OLFACTORY_RECEPTOR_ACTIVITY	421	41	2.37E-19	3.90E-16	*OR10T2:OR10K2:OR10R2:OR6Y1:OR6P1:OR10X1:OR10Z1:OR6K2:OR6K3:OR6K6:OR6N1:OR6N2:OR10J3:OR10J4:OR10J1:OR10J5:OR10J6P:OR4A16:OR5W2:OR5I1:OR5F1:OR5AS1:OR8I2:OR8H2:OR8K5:OR5J2:OR8J2:OR8K3:OR8K1:OR5M11:OR5M10:OR5M1:OR5G3:OR5AK3P:OR5AK2:OR5A1:OR4D6:OR4D10:OR4D11:OR7A5:OR7A10*	http://www.gsea-msigdb.org/gsea/msigdb/cards/GO_OLFACTORY_RECEPTOR_ACTIVITY
GO_bp	GO_SENSORY_PERCEPTION_OF_SMELL	448	41	2.25E-18	1.66E-14	*OR10T2:OR10K2:OR10R2:OR6Y1:OR6P1:OR10X1:OR10Z1:OR6K2:OR6K3:OR6K6:OR6N1:OR6N2:OR10J3:OR10J4:OR10J1:OR10J5:OR10J6P:OR4A16:OR5W2:OR5I1:OR5F1:OR5AS1:OR8I2:OR8H2:OR8K5:OR5J2:OR8J2:OR8K3:OR8K1:OR5M11:OR5M10:OR5M1:OR5G3:OR5AK3P:OR5AK2:OR5A1:OR4D6:OR4D10:OR4D11:OR7A5:OR7A10*	http://www.gsea-msigdb.org/gsea/msigdb/cards/GO_SENSORY_PERCEPTION_OF_SMELL
Positional_gene_sets	chr5q13	106	20	1.34E-15	5.71E-14	*RNU6-1232P:EEF1B2P2:VWA8P1:RPL27AP5:GCNT4:POC5:RNU6-680P:SV2C:IQGAP2:F2RL2:F2R:F2RL1:RN7SL208P:S100Z:RNU6ATAC36P:CRHBP:AGGF1:ZBED3:SNORA47:ZBED3-AS1*	http://www.gsea-msigdb.org/gsea/msigdb/cards/chr5q13

Positional_gene_sets	chr12q23	125	21	3.03E-15	1.13E-13	*ARL1:RNU6-1068P:RPS27P23:RNU5E-5P:RNA5SP367:SPIC:MYBPC1:SYCP3:RNU6-101P:RNA5SP368:RNU6-172P:RNU6-1183P:HSPE1P4:CCDC53:NUP37:PARPBP:PMCH:RN7SL793P:IGF1:RP11-626I20.3:STAB2*	http://www.gsea-msigdb.org/gsea/msigdb/cards/chr12q23
Positional_gene_sets	chr22q12	180	24	6.99E-15	2.32E-13	*SEZ6L:RNA5SP495:ASPHD2:HPS4:SRRD:TPST2:CRYBB1:CRYBA4:ISCA2P1:MIAT:RP1-90L6.3:RNU6-1066P:CTA-929C8.6:MN1:PITPNB:TTC28-AS1:MIR3199-2:RN7SL757P:RN7SL162P:ZNRF3:ZNRF3-IT1:ZNRF3-AS1:RNU6-810P:THOC5*	http://www.gsea-msigdb.org/gsea/msigdb/cards/chr22q12
GO_bp	GO_DETECTION_OF_STIMULUS_INVOLVED_IN_SENSORY_PERCEPTION	523	42	9.75E-17	3.58E-13	*OR10T2:OR10K2:OR10R2:OR6Y1:OR6P1:OR10X1:OR10Z1:OR6K2:OR6K3:OR6K6:OR6N1:OR6N2:OR10J3:OR10J4:OR10J1:OR10J5:OR10J6P:OR4A16:OR5W2:OR5I1:OR5F1:OR5AS1:OR8I2:OR8H2:OR8K5:OR5J2:OR8J2:OR8K3:OR8K1:OR5M11:OR5M10:OR5M1:OR5G3:OR5AK3P:OR5AK2:OR5A1:OR4D6:OR4D10:OR4D11:OR7A5:OR7A10:EYS*	http://www.gsea-msigdb.org/gsea/msigdb/cards/GO_DETECTION_OF_STIMULUS_INVOLVED_IN_SENSORY_PERCEPTION

Reactome	REACTOME_OLFACTORY_SIGNALING_PATHWAY	397	36	3.73E-16	5.59E-13	*OR10T2:OR10K2:OR10R2:OR6Y1:OR6P1:OR10X1:OR10Z1:OR6K2:OR6K3:OR6K6:OR6N1:OR6N2:OR10J3:OR10J1:OR10J5:OR4A16:OR5W2:OR5I1:OR5F1:OR5AS1:OR8I2:OR8H2:OR8K5:OR5J2:OR8K3:OR8K1:OR5M11:OR5M10:OR5M1:OR5AK2:OR5A1:OR4D6:OR4D10:OR4D11:OR7A5:OR7A10*	http://www.gsea-msigdb.org/gsea/msigdb/cards/REACTOME_OLFACTORY_SIGNALING_PATHWAY
Canonical_Pathways	REACTOME_OLFACTORY_SIGNALING_PATHWAY	397	36	3.73E-16	8.20E-13	*OR10T2:OR10K2:OR10R2:OR6Y1:OR6P1:OR10X1:OR10Z1:OR6K2:OR6K3:OR6K6:OR6N1:OR6N2:OR10J3:OR10J1:OR10J5:OR4A16:OR5W2:OR5I1:OR5F1:OR5AS1:OR8I2:OR8H2:OR8K5:OR5J2:OR8K3:OR8K1:OR5M11:OR5M10:OR5M1:OR5AK2:OR5A1:OR4D6:OR4D10:OR4D11:OR7A5:OR7A10*	http://www.gsea-msigdb.org/gsea/msigdb/cards/REACTOME_OLFACTORY_SIGNALING_PATHWAY
GO_bp	GO_SENSORY_PERCEPTION_OF_CHEMICAL_STIMULUS	519	41	4.04E-16	9.90E-13	*OR10T2:OR10K2:OR10R2:OR6Y1:OR6P1:OR10X1:OR10Z1:OR6K2:OR6K3:OR6K6:OR6N1:OR6N2:OR10J3:OR10J4:OR10J1:OR10J5:OR10J6P:OR4A16:OR5W2:OR5I1:OR5F1:OR5AS1:OR8I2:OR8H2:OR8K5:OR5J2:OR8J2:OR8K3:OR8K1:OR5M11:OR5M10:OR5M1:OR5G3:OR5AK3P:OR5AK2:OR5A1:OR4D6:OR4D10:OR4D11:OR7A5:OR7A10*	http://www.gsea-msigdb.org/gsea/msigdb/cards/GO_SENSORY_PERCEPTION_OF_CHEMICAL_STIMULUS

KEGG	KEGG_OLFACTORY_TRANSDUCTION	387	34	6.39E-15	1.19E-12	*OR10T2:OR10K2:OR10R2:OR6Y1:OR10X1:OR10Z1:OR6K2:OR6K3:OR6K6:OR6N1:OR6N2:OR10J3:OR10J1:OR10J5:OR4A16:OR5I1:OR5F1:OR5AS1:OR8I2:OR8H2:OR8K5:OR5J2:OR8K3:OR8K1:OR5M11:OR5M10:OR5M1:OR5AK2:OR5A1:OR4D6:OR4D10:OR4D11:OR7A5:OR7A10*	http://www.gsea-msigdb.org/gsea/msigdb/cards/KEGG_OLFACTORY_TRANSDUCTION
Curated_gene_sets	REACTOME_OLFACTORY_SIGNALING_PATHWAY	397	36	3.73E-16	2.05E-12	*OR10T2:OR10K2:OR10R2:OR6Y1:OR6P1:OR10X1:OR10Z1:OR6K2:OR6K3:OR6K6:OR6N1:OR6N2:OR10J3:OR10J1:OR10J5:OR4A16:OR5W2:OR5I1:OR5F1:OR5AS1:OR8I2:OR8H2:OR8K5:OR5J2:OR8K3:OR8K1:OR5M11:OR5M10:OR5M1:OR5AK2:OR5A1:OR4D6:OR4D10:OR4D11:OR7A5:OR7A10*	http://www.gsea-msigdb.org/gsea/msigdb/cards/REACTOME_OLFACTORY_SIGNALING_PATHWAY
Positional_gene_sets	chr11q14	116	19	1.03E-13	3.07E-12	*RP11-179A16.1:ANKRD42:DLG2:LDHAL6DP:HNRNPA1P72:HNRNPCP6:RNU6-1292P:TMEM126B:TMEM126A:CREBZF:CCDC89:SYTL2:EED:SETP17:C11orf73:RN7SL225P:PTP4A1P6:PRSS23:RNU6-1135P*	http://www.gsea-msigdb.org/gsea/msigdb/cards/chr11q14
Canonical_Pathways	KEGG_OLFACTORY_TRANSDUCTION	387	34	6.39E-15	5.52E-12	*OR10T2:OR10K2:OR10R2:OR6Y1:OR10X1:OR10Z1:OR6K2:OR6K3:OR6K6:OR6N1:OR6N2:OR10J3:OR10J1:OR10J5:OR4A16:OR5I1:OR5F1:OR5AS1:OR8I2:OR8H2:OR8K5:OR5J2:OR8K3:OR8K1:OR5M11:OR5M10:OR5M1:OR5AK2:OR5A1:OR4D6:OR4D10:OR4D11:OR7A5:OR7A10*	http://www.gsea-msigdb.org/gsea/msigdb/cards/KEGG_OLFACTORY_TRANSDUCTION

Canonical_Pathways	REACTOME_G_ALPHA_S_SIGNALLING_EVENTS	539	40	7.53E-15	5.52E-12	*OR10T2:OR10K2:OR10R2:OR6Y1:OR6P1:OR10X1:OR10Z1:OR6K2:OR6K3:OR6K6:OR6N1:OR6N2:OR10J3:OR10J1:OR10J5:HTR7:OR4A16:OR5W2:OR5I1:OR5F1:OR5AS1:OR8I2:OR8H2:OR8K5:OR5J2:OR8K3:OR8K1:OR5M11:OR5M10:OR5M1:OR5AK2:OR5A1:OR4D6:OR4D10:OR4D11:ADCYAP1:OR7A5:OR7A10:INSL3:RLN2*	http://www.gsea-msigdb.org/gsea/msigdb/cards/REACTOME_G_ALPHA_S_SIGNALLING_EVENTS
Reactome	REACTOME_G_ALPHA_S_SIGNALLING_EVENTS	539	40	7.53E-15	5.64E-12	*OR10T2:OR10K2:OR10R2:OR6Y1:OR6P1:OR10X1:OR10Z1:OR6K2:OR6K3:OR6K6:OR6N1:OR6N2:OR10J3:OR10J1:OR10J5:HTR7:OR4A16:OR5W2:OR5I1:OR5F1:OR5AS1:OR8I2:OR8H2:OR8K5:OR5J2:OR8K3:OR8K1:OR5M11:OR5M10:OR5M1:OR5AK2:OR5A1:OR4D6:OR4D10:OR4D11:ADCYAP1:OR7A5:OR7A10:INSL3:RLN2*	http://www.gsea-msigdb.org/gsea/msigdb/cards/REACTOME_G_ALPHA_S_SIGNALLING_EVENTS
GO_mf	GO_G_PROTEIN_COUPLED_RECEPTOR_ACTIVITY	857	51	1.04E-14	8.54E-12	*OR10T2:OR10K2:OR10R2:OR6Y1:OR6P1:OR10X1:OR10Z1:OR6K2:OR6K3:OR6K6:OR6N1:OR6N2:DARC:OR10J3:OR10J4:OR10J1:OR10J5:OR10J6P:HTR7:PRLHR:OR4A16:OR5W2:OR5I1:OR5F1:OR5AS1:OR8I2:OR8H2:OR8K5:OR5J2:OR8J2:OR8K3:OR8K1:OR5M11:OR5M10:OR5M1:OR5G3:OR5AK3P:OR5AK2:APLNR:OR5A1:OR4D6:OR4D10:OR4D11:GPR133:OR7A5:OR7A10:F2RL3:CXCR4:F2RL2:F2R:F2RL1*	http://www.gsea-msigdb.org/gsea/msigdb/cards/GO_G_PROTEIN_COUPLED_RECEPTOR_ACTIVITY

Curated_gene_sets	KEGG_OLFACTORY_TRANSDUCTION	387	34	6.39E-15	1.38E-11	*OR10T2:OR10K2:OR10R2:OR6Y1:OR10X1:OR10Z1:OR6K2:OR6K3:OR6K6:OR6N1:OR6N2:OR10J3:OR10J1:OR10J5:OR4A16:OR5I1:OR5F1:OR5AS1:OR8I2:OR8H2:OR8K5:OR5J2:OR8K3:OR8K1:OR5M11:OR5M10:OR5M1:OR5AK2:OR5A1:OR4D6:OR4D10:OR4D11:OR7A5:OR7A10*	http://www.gsea-msigdb.org/gsea/msigdb/cards/KEGG_OLFACTORY_TRANSDUCTION
Curated_gene_sets	REACTOME_G_ALPHA_S_SIGNALLING_EVENTS	539	40	7.53E-15	1.38E-11	*OR10T2:OR10K2:OR10R2:OR6Y1:OR6P1:OR10X1:OR10Z1:OR6K2:OR6K3:OR6K6:OR6N1:OR6N2:OR10J3:OR10J1:OR10J5:HTR7:OR4A16:OR5W2:OR5I1:OR5F1:OR5AS1:OR8I2:OR8H2:OR8K5:OR5J2:OR8K3:OR8K1:OR5M11:OR5M10:OR5M1:OR5AK2:OR5A1:OR4D6:OR4D10:OR4D11:ADCYAP1:OR7A5:OR7A10:INSL3:RLN2*	http://www.gsea-msigdb.org/gsea/msigdb/cards/REACTOME_G_ALPHA_S_SIGNALLING_EVENTS
GO_mf	GO_ODORANT_BINDING	94	18	2.70E-14	1.48E-11	*OR5W2:OR5I1:OR5F1:OR5AS1:OR8I2:OR8H2:OR8K5:OR5J2:OR8J2:OR8K3:OR8K1:OR5M11:OR5M10:OR5M1:OR5G3:OR5AK3P:OR5AK2:OR5A1*	http://www.gsea-msigdb.org/gsea/msigdb/cards/GO_ODORANT_BINDING
Positional_gene_sets	chr10q26	173	21	2.20E-12	5.97E-11	*EMX2OS:EMX2:SLC25A18P1:PRLHR:RN7SL846P:PHACTR2P1:MIR4682:NACAP2:PPAPDC1A:C10orf85:WDR11-AS1:WDR11:FGFR2:RPS15AP5:RNU6-728P:PLEKHA1:MIR3941:CUZD1:FAM24B:C10orf88:PSTK*	http://www.gsea-msigdb.org/gsea/msigdb/cards/chr10q26

GO_bp	GO_DETECTION_OF_STIMULUS	682	43	2.03E-13	3.73E-10	*CD1D:OR10T2:OR10K2:OR10R2:OR6Y1:OR6P1:OR10X1:OR10Z1:OR6K2:OR6K3:OR6K6:OR6N1:OR6N2:OR10J3:OR10J4:OR10J1:OR10J5:OR10J6P:OR4A16:OR5W2:OR5I1:OR5F1:OR5AS1:OR8I2:OR8H2:OR8K5:OR5J2:OR8J2:OR8K3:OR8K1:OR5M11:OR5M10:OR5M1:OR5G3:OR5AK3P:OR5AK2:OR5A1:OR4D6:OR4D10:OR4D11:OR7A5:OR7A10:EYS*	http://www.gsea-msigdb.org/gsea/msigdb/cards/GO_DETECTION_OF_STIMULUS
GO_bp	GO_SENSORY_PERCEPTION	960	51	8.06E-13	1.18E-09	*OR10T2:OR10K2:OR10R2:OR6Y1:OR6P1:OR10X1:OR10Z1:OR6K2:OR6K3:OR6K6:OR6N1:OR6N2:OR10J3:OR10J4:OR10J1:OR10J5:OR10J6P:OR4A16:OR5W2:OR5I1:OR5F1:OR5AS1:OR8I2:OR8H2:OR8K5:OR5J2:OR8J2:OR8K3:OR8K1:OR5M11:OR5M10:OR5M1:OR5G3:OR5AK3P:OR5AK2:TIMM10:OR5A1:OR4D6:OR4D10:OR4D11:DLG2:ADCYAP1:OR7A5:OR7A10:RAB3A:CRYBB1:CRYBA4:SOX14:F2R:TFAP2A:EYS*	http://www.gsea-msigdb.org/gsea/msigdb/cards/GO_SENSORY_PERCEPTION
Positional_gene_sets	chr3p26	39	10	7.24E-10	1.81E-08	*RN7SL120P:CNTN6:RPL23AP39:RPL21P17:RN7SKP144:CNTN4:DNAJC19P4:CNTN4-AS1:CRBN:SETMAR*	http://www.gsea-msigdb.org/gsea/msigdb/cards/chr3p26
Positional_gene_sets	chr10p15	66	12	1.05E-09	2.41E-08	*RNA5SP297:DIP2C:RNA5SP298:RN7SL754P:MIR5699:PRR26:LARP4B:GTPBP4:IDI1:WDR37:PFKP:PITRM1-AS1*	http://www.gsea-msigdb.org/gsea/msigdb/cards/chr10p15
GWAScatalog	Neutrophil count in HIV-infection	6	6	2.28E-11	4.14E-08	*PYHIN1:IFI16:AIM2:CADM3:DARC:MPTX1*	

Reactome	REACTOME_SIGNALING_BY_GPCR	1169	52	3.14E-10	1.57E-07	*OR10T2:OR10K2:OR10R2:OR6Y1:OR6P1:OR10X1:OR10Z1:OR6K2:OR6K3:OR6K6:OR6N1:OR6N2:DARC:OR10J3:OR10J1:OR10J5:HTR7:PRLHR:OR4A16:OR5W2:OR5I1:OR5F1:OR5AS1:OR8I2:OR8H2:OR8K5:OR5J2:OR8K3:OR8K1:OR5M11:OR5M10:OR5M1:OR5AK2:APLNR:OR5A1:OR4D6:OR4D10:OR4D11:PPP2R1B:PMCH:ADCYAP1:OR7A5:OR7A10:F2RL3:INSL3:CXCR4:F2RL2:F2R:F2RL1:CRHBP:RLN2:AWAT2*	http://www.gsea-msigdb.org/gsea/msigdb/cards/REACTOME_SIGNALING_BY_GPCR
Positional_gene_sets	chrXq13	132	15	7.74E-09	1.65E-07	*ACTR3P2:SERBP1P1:PJA1:HMGN1P35:LINC00269:CYCSP43:FAM155B:EDA:MIR676:AWAT2:OTUD6A:MTND4P31:IGBP1:RNU1-56P:SOCS5P4*	http://www.gsea-msigdb.org/gsea/msigdb/cards/chrXq13
Canonical_Pathways	REACTOME_SIGNALING_BY_GPCR	1169	52	3.14E-10	1.73E-07	*OR10T2:OR10K2:OR10R2:OR6Y1:OR6P1:OR10X1:OR10Z1:OR6K2:OR6K3:OR6K6:OR6N1:OR6N2:DARC:OR10J3:OR10J1:OR10J5:HTR7:PRLHR:OR4A16:OR5W2:OR5I1:OR5F1:OR5AS1:OR8I2:OR8H2:OR8K5:OR5J2:OR8K3:OR8K1:OR5M11:OR5M10:OR5M1:OR5AK2:APLNR:OR5A1:OR4D6:OR4D10:OR4D11:PPP2R1B:PMCH:ADCYAP1:OR7A5:OR7A10:F2RL3:INSL3:CXCR4:F2RL2:F2R:F2RL1:CRHBP:RLN2:AWAT2*	http://www.gsea-msigdb.org/gsea/msigdb/cards/REACTOME_SIGNALING_BY_GPCR

GO_mf	GO_TRANSMEMBRANE_SIGNALING_RECEPTOR_ACTIVITY	1253	54	4.38E-10	1.80E-07	*OR10T2:OR10K2:OR10R2:OR6Y1:OR6P1:OR10X1:OR10Z1:OR6K2:OR6K3:OR6K6:OR6N1:OR6N2:DARC:OR10J3:OR10J4:OR10J1:OR10J5:OR10J6P:CD247:HTR7:PRLHR:FGFR2:OR4A16:OR5W2:OR5I1:OR5F1:OR5AS1:OR8I2:OR8H2:OR8K5:OR5J2:OR8J2:OR8K3:OR8K1:OR5M11:OR5M10:OR5M1:OR5G3:OR5AK3P:OR5AK2:APLNR:OR5A1:OR4D6:OR4D10:OR4D11:GPR133:NTRK3:OR7A5:OR7A10:F2RL3:CXCR4:F2RL2:F2R:F2RL1*	http://www.gsea-msigdb.org/gsea/msigdb/cards/GO_TRANSMEMBRANE_SIGNALING_RECEPTOR_ACTIVITY
GO_bp	GO_G_PROTEIN_COUPLED_RECEPTOR_SIGNALING_PATHWAY	1363	58	1.53E-10	1.88E-07	*OR10T2:OR10K2:OR10R2:OR6Y1:OR6P1:OR10X1:OR10Z1:OR6K2:OR6K3:OR6K6:OR6N1:OR6N2:DARC:OR10J3:OR10J4:OR10J1:OR10J5:OR10J6P:HTR7:PRLHR:OR4A16:OR5W2:OR5I1:OR5F1:OR5AS1:OR8I2:OR8H2:OR8K5:OR5J2:OR8J2:OR8K3:OR8K1:OR5M11:OR5M10:OR5M1:OR5G3:OR5AK3P:OR5AK2:APLNR:OR5A1:OR4D6:OR4D10:OR4D11:PMCH:GPR133:ADCYAP1:OR7A5:OR7A10:F2RL3:INSL3:CXCR4:NXPH2:IQGAP2:F2RL2:F2R:F2RL1:JAK2:RLN2*	http://www.gsea-msigdb.org/gsea/msigdb/cards/GO_G_PROTEIN_COUPLED_RECEPTOR_SIGNALING_PATHWAY

Curated_gene_sets	REACTOME_SIGNALING_BY_GPCR	1169	52	3.14E-10	4.32E-07	*OR10T2:OR10K2:OR10R2:OR6Y1:OR6P1:OR10X1:OR10Z1:OR6K2:OR6K3:OR6K6:OR6N1:OR6N2:DARC:OR10J3:OR10J1:OR10J5:HTR7:PRLHR:OR4A16:OR5W2:OR5I1:OR5F1:OR5AS1:OR8I2:OR8H2:OR8K5:OR5J2:OR8K3:OR8K1:OR5M11:OR5M10:OR5M1:OR5AK2:APLNR:OR5A1:OR4D6:OR4D10:OR4D11:PPP2R1B:PMCH:ADCYAP1:OR7A5:OR7A10:F2RL3:INSL3:CXCR4:F2RL2:F2R:F2RL1:CRHBP:RLN2:AWAT2*	http://www.gsea-msigdb.org/gsea/msigdb/cards/REACTOME_SIGNALING_BY_GPCR
Positional_gene_sets	chr4q28	58	10	4.46E-08	8.78E-07	*SPRY1:LINC01091:TECRP2:RP11-93I21.3:ANKRD50:FAT4:NUPL1P1:TMEM248P1:RBM48P1:SLC25A31*	http://www.gsea-msigdb.org/gsea/msigdb/cards/chr4q28
Positional_gene_sets	chr5q11	74	11	4.70E-08	8.78E-07	*MIER3:SALL4P1:ACTBL2:RP11-478P10.1:PGAM1P1:PLK2:GAPT:MIR548AE2:CTD-2117L12.1:RAB3C:RPL5P15*	http://www.gsea-msigdb.org/gsea/msigdb/cards/chr5q11
Positional_gene_sets	chr6p25	61	10	7.36E-08	1.29E-06	*LYRM4:MIR3691:FARS2:PKMP5:F13A1:MIR5683:LY86-AS1:SNAPC5P1:CNN3P1:LY86*	http://www.gsea-msigdb.org/gsea/msigdb/cards/chr6p25
GWAScatalog	C-reactive protein	31	9	1.54E-09	1.40E-06	*IFI16:DARC:FCER1A:OR10J3:OR10J1:OR10J5:CRPP1:CRP:DUSP23*	

GO_mf	GO_MOLECULAR_TRANSDUCER_ACTIVITY	1518	57	1.99E-08	6.55E-06	*FCRL1:OR10T2:OR10K2:OR10R2:OR6Y1:OR6P1:OR10X1:OR10Z1:OR6K2:OR6K3:OR6K6:OR6N1:OR6N2:DARC:OR10J3:OR10J4:OR10J1:OR10J5:OR10J6P:RXRG:GPA33:CD247:HTR7:PRLHR:FGFR2:OR4A16:OR5W2:OR5I1:OR5F1:OR5AS1:OR8I2:OR8H2:OR8K5:OR5J2:OR8J2:OR8K3:OR8K1:OR5M11:OR5M10:OR5M1:OR5G3:OR5AK3P:OR5AK2:APLNR:OR5A1:OR4D6:OR4D10:OR4D11:GPR133:NTRK3:OR7A5:OR7A10:F2RL3:CXCR4:F2RL2:F2R:F2RL1*	http://www.gsea-msigdb.org/gsea/msigdb/cards/GO_MOLECULAR_TRANSDUCER_ACTIVITY
GO_bp	GO_NERVOUS_SYSTEM_PROCESS	1409	55	9.74E-09	1.02E-05	*OR10T2:OR10K2:OR10R2:OR6Y1:OR6P1:OR10X1:OR10Z1:OR6K2:OR6K3:OR6K6:OR6N1:OR6N2:OR10J3:OR10J4:OR10J1:OR10J5:OR10J6P:LMX1A:OR4A16:OR5W2:OR5I1:OR5F1:OR5AS1:OR8I2:OR8H2:OR8K5:OR5J2:OR8J2:OR8K3:OR8K1:OR5M11:OR5M10:OR5M1:OR5G3:OR5AK3P:OR5AK2:TIMM10:OR5A1:OR4D6:OR4D10:OR4D11:DLG2:NTRK3:ADCYAP1:OR7A5:OR7A10:RAB3A:CRYBB1:CRYBA4:SOX14:PLK2:F2R:CRHBP:TFAP2A:EYS*	http://www.gsea-msigdb.org/gsea/msigdb/cards/GO_NERVOUS_SYSTEM_PROCESS
Positional_gene_sets	chr15q25	135	12	3.42E-06	5.69E-05	*RNU6-1339P:RNU6-231P:MIR548AP:RP11-553E24.2:RNA5SP400:AGBL1:AGBL1-AS1:RNU6-185P:LINC00052:NTRK3:MED28P6:NTRK3-AS1*	http://www.gsea-msigdb.org/gsea/msigdb/cards/chr15q25

Positional_gene_sets	chr2q22	39	7	3.67E-06	5.77E-05	*CXCR4:HNMT:AC069394.1:YWHAEP5:SPOPL:NXPH2:RN7SL283P*	http://www.gsea-msigdb.org/gsea/msigdb/cards/chr2q22
Positional_gene_sets	chr19p13	706	30	4.81E-06	7.20E-05	*SNORD37:SNORA68:SNORD37:SNORA68:SNORD37:OR7A5:OR7A10:MED26:SIN3B:F2RL3:RN7SL835P:RN7SL823P:ANO8:GTPBP3:TMEM221:SLC27A1:PGLS:FAM129C:COLGALT1:UNC13A:MAP1S:FCHO1:INSL3:JAK3:RPL18A:SNORA68:SLC5A5:CCDC124:KCNN1:ARRDC2:IFI30:MPV17L2:RAB3A:KXD1:SNORA68:SNORA68*	http://www.gsea-msigdb.org/gsea/msigdb/cards/chr19p13
GO_mf	GO_THROMBIN_ACTIVATED_RECEPTOR_ACTIVITY	5	4	3.99E-07	9.38E-05	*F2RL3:F2RL2:F2R:F2RL1*	http://www.gsea-msigdb.org/gsea/msigdb/cards/GO_THROMBIN_ACTIVATED_RECEPTOR_ACTIVITY
GO_mf	GO_GLYCINE_N_ACYLTRANSFERASE_ACTIVITY	5	4	3.99E-07	9.38E-05	*GLYAT:GLYATL2:GLYATL1:GLYATL1P3*	http://www.gsea-msigdb.org/gsea/msigdb/cards/GO_GLYCINE_N_ACYLTRANSFERASE_ACTIVITY
Positional_gene_sets	chr6q12	19	5	1.30E-05	0.000185	*RNU6-1221P:EYS:GCNT1P4:ADH5P4:RNU6-280P:RP11-406O16.1*	http://www.gsea-msigdb.org/gsea/msigdb/cards/chr6q12
GO_bp	GO_THROMBIN_ACTIVATED_RECEPTOR_SIGNALING_PATHWAY	12	5	9.76E-07	0.000896	*F2RL3:IQGAP2:F2RL2:F2R:F2RL1*	http://www.gsea-msigdb.org/gsea/msigdb/cards/GO_THROMBIN_ACTIVATED_RECEPTOR_SIGNALING_PATHWAY
GWAScatalog	Select biomarker traits	14	5	2.40E-06	0.001451	*CADM3:FCER1A:OR10J3:OR10J1:CRP*	

Positional_gene_sets	chr10q23	169	11	0.000154	0.002086	*ACTA2-AS1:LIPA:PANK1:KIF20B:LINC00865:RP11-478K7.2:SNRPD2P1:RN7SKP143:RP11-15K3.1:HTR7:RPP30*	http://www.gsea-msigdb.org/gsea/msigdb/cards/chr10q23
GWAScatalog	Obesity (extreme) (SNP x SNP interaction)	3	3	4.81E-06	0.002182	*OR10R1P:OR6Y1:OR6P1*	
Positional_gene_sets	chr18q22	51	6	0.000215	0.002796	*DSEL:RP11-638L3.3:AKR1B10P2:TMX3:CCDC102B:RPS2P6*	http://www.gsea-msigdb.org/gsea/msigdb/cards/chr18q22
Positional_gene_sets	chr8q23	34	5	0.000252	0.00314	*RP11-946L20.4:EEF1A1P37:CSMD3:RPL30P16:MIR2053*	http://www.gsea-msigdb.org/gsea/msigdb/cards/chr8q23
Hallmark_gene_sets	HALLMARK_BILE_ACID_METABOLISM	112	9	0.000125	0.006237	*RXRG:ALDH9A1:IDI1:PXMP2:ABCA8:ABCA9:ABCA6:ABCA5:BMP6*	http://www.gsea-msigdb.org/gsea/msigdb/cards/HALLMARK_BILE_ACID_METABOLISM
Positional_gene_sets	chr14q12	86	7	0.000647	0.007737	*OR7K1P:RP11-626P14.2:RP11-384J4.1:MIR4307:BNIP3P1:RPL26P3:EIF4A1P12*	http://www.gsea-msigdb.org/gsea/msigdb/cards/chr14q12
Wikipathways	Amino acid conjugation of benzoic acid	4	3	1.90E-05	0.010315	*GLYAT:GLYATL2:GLYATL1*	http://www.wikipathways.org/instance/WP521_r106159
Positional_gene_sets	chr4q27	27	4	0.001042	0.011988	*IL21:IL21-AS1:CETN4P:BBS12*	http://www.gsea-msigdb.org/gsea/msigdb/cards/chr4q27

GWAScatalog	Night sleep phenotypes	532	23	4.68E-05	0.016972	*OR6P1:OR10X1:OR10Z1:SPTA1:PBX1:PXMP2:MIR1179:MIR7-2:DSEL:RP11-638L3.1:RPS2P6:RPL21P17:CNTN4:IL21:IL21-AS1:CETN4P:BBS12:SALL4P1:ACTBL2:PKMP5:F13A1:MIR5683:OFCC1*	
Positional_gene_sets	chr11q22	80	6	0.002378	0.026335	*RPS2P39:C11orf87:TFAMP2:ZC3H12C:RDX:FDX1*	http://www.gsea-msigdb.org/gsea/msigdb/cards/chr11q22
GWAScatalog	Breast cancer in BRCA2 mutation carriers	6	3	9.26E-05	0.028013	*FGFR2:TFAP2A:LINC00518*	
Positional_gene_sets	chr11q23	174	9	0.002963	0.03164	*ARHGAP20:RP11-89C3.4:RPS17P15:C11orf53:MIR4491:POU2AF1:RNU2-60P:LAYN:PPP2R1B*	http://www.gsea-msigdb.org/gsea/msigdb/cards/chr11q23
Reactome	REACTOME_CONJUGATION_OF_BENZOATE_WITH_GLYCINE	6	3	9.26E-05	0.034703	*GLYAT:GLYATL2:GLYATL1*	http://www.gsea-msigdb.org/gsea/msigdb/cards/REACTOME_CONJUGATION_OF_BENZOATE_WITH_GLYCINE
Canonical_Pathways	REACTOME_CONJUGATION_OF_BENZOATE_WITH_GLYCINE	6	3	9.26E-05	0.040727	*GLYAT:GLYATL2:GLYATL1*	http://www.gsea-msigdb.org/gsea/msigdb/cards/REACTOME_CONJUGATION_OF_BENZOATE_WITH_GLYCINE

Supplementary Table 7. External replication with Wooton at al's top sequence variants associated with social trust. 44 variants were available in our dataset and are shown below.

RS	Gene	ALLELE1	ALLELE0	A1FREQ	BETA Wootton	P Wootton	BETA DBDS	P DBDS
rs1018991	*CNTNAP2*	G	A	0.1177	-0.72	4.22E-06	0.0455	0.03543
rs10228191	*CNTNAP2*	T	C	0.1093	-0.73	5.25E-06	0.0387	0.08232
rs10264424	*CNTNAP2*	A	G	0.1099	0.73	5.41E-06	0.0384	0.08426
rs10265626	*CNTNAP2*	A	C	0.1074	0.72	7.17E-06	0.0432	0.05404
rs10267232	*CNTNAP2*	T	C	0.1175	-0.73	3.55E-06	0.0452	0.03666
rs1528532	*CNTNAP2*	T	C	0.1098	-0.79	5.79E-06	0.0386	0.08295
rs6464815	*CNTNAP2*	T	C	0.1343	-0.71	3.55E-06	0.0463	0.02324
rs6973129	*CNTNAP2*	A	C	0.1177	0.71	6.24E-06	0.0438	0.04245
rs700313	*CNTNAP2*	A	C	0.1107	0.71	9.09E-06	0.0387	0.08048
rs7778880	*CNTNAP2*	A	G	0.1165	0.71	6.56E-06	0.046	0.03351
rs7779464	*CNTNAP2*	A	G	0.1165	0.71	6.40E-06	0.046	0.03355
rs826647	*CNTNAP2*	A	G	0.1092	0.73	4.71E-06	0.039	0.0798
rs826650	*CNTNAP2*	G	A	0.1092	-0.73	4.64E-06	0.0392	0.07877
rs826651	*CNTNAP2*	T	C	0.1092	-0.73	4.37E-06	0.0391	0.07914
rs826655	*CNTNAP2*	T	C	0.1104	-0.7	1.03E-05	0.0397	0.07307
rs826656	*CNTNAP2*	T	C	0.1107	-0.71	8.07E-06	0.0385	0.08221
rs826657	*CNTNAP2*	T	C	0.1107	-0.71	1.03E-05	0.0386	0.08118
rs826658	*CNTNAP2*	C	T	0.1107	0.71	8.15E-06	0.0385	0.08198
rs826659	*CNTNAP2*	T	C	0.1107	-0.71	8.17E-06	0.0386	0.08162
rs826661	*CNTNAP2*	C	T	0.1107	0.71	8.27E-06	0.0386	0.08157
rs10170802	*GCFC2;LRRTM4*	C	T	0.0693	1.1	3.88E-06	-0.0143	0.5975
rs12473082	*GCFC2;LRRTM4*	T	G	0.0689	-1.06	7.79E-06	-0.0182	0.5005
rs1372007	*GCFC2;LRRTM4*	G	T	0.0692	1.14	2.85E-06	-0.0143	0.5973
rs1404089	*GCFC2;LRRTM4*	G	C	0.0692	-1.14	2.85E-06	-0.0143	0.5972
rs1524214	*GCFC2;LRRTM4*	C	A	0.0692	-1.14	2.85E-06	-0.0143	0.5972
rs1524216	*GCFC2;LRRTM4*	A	C	0.0687	1.15	1.75E-06	-0.0203	0.4549
rs1608588	*GCFC2;LRRTM4*	T	C	0.0688	-1.14	2.85E-06	-0.0158	0.5594
rs1660568	*GCFC2;LRRTM4*	T	C	0.0687	-1.08	6.22E-06	-0.0158	0.5596

rs2140571	*GCFC2;LRRTM4*	C	T	0.0688	1.16	1.58E-06	-0.0205	0.4494
rs2901742	*GCFC2;LRRTM4*	G	T	0.0691	1.1	3.94E-06	-0.0134	0.6193
rs317288	*GCFC2;LRRTM4*	A	G	0.0687	1.14	2.90E-06	-0.0156	0.5653
rs317290	*GCFC2;LRRTM4*	G	T	0.0691	1.13	2.84E-06	-0.0133	0.6232
rs425915	*GCFC2;LRRTM4*	C	T	0.0691	1.14	2.59E-06	-0.0132	0.6256
rs449418	*GCFC2;LRRTM4*	G	A	0.0691	-1.13	2.60E-06	-0.0134	0.6194
rs6753413	*GCFC2;LRRTM4*	T	C	0.0689	-1.14	2.85E-06	-0.0145	0.5933
rs6759874	*GCFC2;LRRTM4*	T	C	0.0693	-1.14	2.66E-06	-0.0125	0.6433
rs7321687	*GPC6*	T	G	0.3159	-0.51	7.17E-06	-0.0024	0.8734
rs9516371	*GPC6*	T	C	0.341	-0.48	1.03E-05	-0.0024	0.8709
rs4145891	*LINC00540;LINC0062*	A	T	0.1306	0.67	6.83E-06	0.0024	0.9115
rs10824657	*LINC00595;ZMIZ1-AS*	A	G	0.2135	-0.6	1.06E-06	0.0079	0.6407
rs10501366	*LRRC55;APLNR*	A	G	0.2799	0.54	9.74E-06	0.0029	0.8535
rs2239879	*MTMR7*	C	T	0.3299	0.52	7.02E-06	0.012	0.417
rs7005753	*MTMR7*	G	T	0.3467	0.5	6.81E-06	0.0132	0.3679
rs12700790	*SDK1*	A	G	0.0888	0.8	6.76E-06	-0.0415	0.08639

Supplementary Table 8 *. Studies used for computing genetic correlation with social trust. Summary statistics for 14 psychiatric phenotypes were obtained from the Psychiatric Genomics Consortium resource; those for 5 personality traits were obtained from the corresponding publications.*

Phenotype	Reference	Summary statistics	Sample size	Acronym
ADHD	https://pubmed.ncbi.nlm.nih.gov/30478444/	https://pgc.unc.edu/for-researchers/download-results/	55374	ADHD
Alcohol dependence	https://pubmed.ncbi.nlm.nih.gov/30482948/	https://pgc.unc.edu/for-researchers/download-results/	46568	AUD
Alzheimers disease	https://pubmed.ncbi.nlm.nih.gov/30617256/	https://pgc.unc.edu/for-researchers/download-results/	455258	AD
Anorexia nervosa	https://pubmed.ncbi.nlm.nih.gov/31308545/	https://pgc.unc.edu/for-researchers/download-results/	72517	AN
Anxiety disorders (factor score)	https://pubmed.ncbi.nlm.nih.gov/26754954/	https://pgc.unc.edu/for-researchers/download-results/	18186	ANX
Autism spectrum disorder	https://pubmed.ncbi.nlm.nih.gov/30804558/	https://pgc.unc.edu/for-researchers/download-results/	46350	ASD
Bipolar disorder	https://pubmed.ncbi.nlm.nih.gov/34002096/	https://pgc.unc.edu/for-researchers/download-results/	413466	BIP
Depressive symptoms	https://www.nature.com/articles/s41588-018-0320-9	https://pgc.unc.edu/for-researchers/download-results/	1067913	DEP
Major depressive disorder	https://pubmed.ncbi.nlm.nih.gov/29700475/	https://pgc.unc.edu/for-researchers/download-results/	480359	MDD
OCD	https://pubmed.ncbi.nlm.nih.gov/28761083/	https://pgc.unc.edu/for-researchers/download-results/	9725	OCD
PTSD	https://pubmed.ncbi.nlm.nih.gov/31594949/	https://pgc.unc.edu/for-researchers/download-results/	206655	PTSD
Schizophrenia	https://pubmed.ncbi.nlm.nih.gov/35396580/	https://pgc.unc.edu/for-researchers/download-results/	320404	SCZ
Tourette's syndrome	https://pubmed.ncbi.nlm.nih.gov/30818990/	https://pgc.unc.edu/for-researchers/download-results/	14307	TS

Neuroticism	https://www.nature.com/articles/s41588-018-0320-8	https://surfdrive.surf.nl/files/index.php/s/Ow1qCDpFT421ZOO/download?path=%2FMultivariate_GWAMA_sumstats%2FUnivariate&files=Uni_NEU_no23andMe.txt.gz	523783	NEU
Openness	https://www.nature.com/articles/mp2010128#Sec29	https://tweelingenregister.vu.nl/gpc	17375	OPN
Extraversion	https://www.nature.com/articles/mp2010128#Sec29	https://tweelingenregister.vu.nl/gpc	17375	EXTR
Conscientiousness	https://www.nature.com/articles/mp2010128#Sec29	https://tweelingenregister.vu.nl/gpc	17375	CONS
Agreeableness	https://www.nature.com/articles/mp2010128#Sec29	https://tweelingenregister.vu.nl/gpc	17375	AGR

Supplementary Table 9. Full table of PheWAS associations between discovery lead sequence variant, rs12776883, and phenotypes found in the GWAS ATLAS resource (https://atlas.ctglab.nl/). Associations are sorted by Bonferroni-corrected p-value.

atlas ID	PMID	Year	Domain	Trait	P-value	P-adj	N	EA	NEA	File	Consortium	Population
4553	31676860	2019	Neurological	Left isthmus cingulate	6.72E-04	0.06	21821	T	C	https://www.med.unc.edu/bigs2/data/gwas-summary-statistics/	NA	UKB2 (EUR meta)
4452	31676860	2019	Neurological	Left isthmus cingulate	8.42E-04	0.076	19629	T	C	https://www.med.unc.edu/bigs2/data/gwas-summary-statistics/	NA	UKB2 (EUR)
999	27989323	2017	Immunological	Monocyte specific chemokine 3 (CCL7)	9.64E-04	0.087	843	C	T	http://www.computationalmedicine.fi/data#Cytokine_GWAS	NA	EUR
397	24816252	2014	Metabolic	Amino acid::Phenylalanine & tyrosine metabolism::phenylacetate	0.00282	0.254	4754	T	C	http://mips.helmholtz-muenchen.de/proj/GWAS/gwas/gwas_server/shin_et_al.metal.out.tar.gz	NA	EUR
1225	25607358	2015	Neurological	Mean Thalamus	0.00377	0.339	13171	T	C	http://enigma.ini.usc.edu/research/download-enigma-gwas-results/	ENIGMA	EUR
4255	30952852	2019	Psychiatric	Sleep efficiency	0.0039	0.351	84810	C	T	https://personal.broadinstitute.org/mvon/accel_GWAS_all_BOLT.output_HRC.only_plus.metrics_maf0.001_hwep1em12_info0.3.txt.zip	NA	UKB2 (EUR)
336	25772697	2015	Immunological	NKeff:%2-158a+	0.00398	0.358	669	T	C	ftp://twinr-ftp.kcl.ac.uk/ImmuneCellScience/2-GWASResults/GWA_P4_3553.txt.gz	NA	EUR

3241	31427789	2019	Nutritional	Salad / raw vegetable intake	0.00483	0.435	363780	T	C	https://atlas.ctglab.nl/ukb2_sumstats/f.1299.0.0_res.EUR.sumstats.MACfilt.txt.gz	NA	UKB2 (EUR)
3539	31427789	2019	Activities	Reason for glasses/contact lenses: For short-sightedness, i.e. only or mainly for distance viewing such as driving, cinema etc (called 'myopia')	0.00533	0.48	78647	C	T	https://atlas.ctglab.nl/ukb2_sumstats/6147_1_logistic.EUR.sumstats.MACfilt.txt.gz	NA	UKB2 (EUR)
4014	29700475	2018	Psychiatric	Major depressive disorder	0.00581	0.523	173005	T	C	https://www.med.unc.edu/pgc/results-and-downloads/downloads	PGC	UKB1 (EUR meta)
334	25772697	2015	Immunological	NKeff:%Kir (1)	0.00628	0.565	669	T	C	ftp://twinr-ftp.kcl.ac.uk/ImmuneCellScience/2-GWASResults/GWA_P4_3546.txt.gz	NA	EUR
4280	30804560	2019	Respiratory	FEV1/FVC ratio	0.00671	0.604	400102	C	T	ftp://ftp.ebi.ac.uk/pub/databases/gwas/summary_statistics/ShrineN_30804560_GCST007431/Shrine_30804560_FEV1_to_FVC_RATIO_meta-analysis.txt.gz	NA	UKB2 (EUR meta)
338	25772697	2015	Immunological	NKeff:%Kir+ (3)	0.00709	0.638	669	T	C	ftp://twinr-ftp.kcl.ac.uk/ImmuneCellScience/2-GWASResults/GWA_P4_3763.txt.gz	NA	EUR
4586	31676860	2019	Neurological	Right lateral orbitofrontal	0.00832	0.749	21821	T	C	https://www.med.unc.edu/bigs2/data/gwas-summary-statistics/	NA	UKB2 (EUR meta)

337	25772697	2015	Immunological	NKeff:%Kir+ (2)	0.00895	0.806	669	C	T	ftp://twinr-ftp.kcl.ac.uk/ImmuneCellScience/2-GWASResults/GWA_P4_3636.txt.gz	NA	EUR
3541	31427789	2019	Activities	Reason for glasses/contact lenses: For just reading/near work as you are getting older (called 'presbyopia')	0.00947	0.853	78647	T	C	https://atlas.ctglab.nl/ukb2_sumstats/6147_3_logistic.EUR.sumstats.MACfilt.txt.gz	NA	UKB2 (EUR)
3815	20045101	2010	Immunological	CD4:CD8 lymphocyte ratio	0.0112	1	2538	NA	NA	https://genepi.qimr.edu.au/staff/manuelF/gwas_results/CD4_8.assoc.gz	NA	EUR
4293	30718901	2019	Psychiatric	Depression	0.01175	1	500199	T	C	https://datashare.is.ed.ac.uk/bitstream/handle/10283/3203/PGC_UKB_depression_genome-wide.txt	NA	UKB2 (EUR meta)
1002	27989323	2017	Immunological	Monokine induced by interferon-gamma (CXCL9)	0.01191	1	3685	T	C	http://www.computationalmedicine.fi/data#Cytokine_GWAS	NA	EUR
592	24816252	2014	Metabolic	Peptide::Dipeptide::cyclo(leu-pro)	0.01198	1	4833	C	T	http://mips.helmholtz-muenchen.de/proj/GWAS/gwas/gwas_server/shin_et_al.metal.out.tar.gz	NA	EUR
400	24816252	2014	Metabolic	Amino acid::Phenylalanine & tyrosine metabolism::phenyllactate (PLA)	0.01265	1	6064	C	T	http://mips.helmholtz-muenchen.de/proj/GWAS/gwas/gwas_server/shin_et_al.metal.out.tar.gz	NA	EUR

602	24816252	2014	Metabolic	Peptide::gamma-glutamyl::gamma-glutamylglutamine	0.01276	1	7662	T	C	http://mips.helmholtz-muenchen.de/proj/GWAS/gwas/gwas_server/shin_et_al.metal.out.tar.gz	NA	EUR
704	24816252	2014	Metabolic	::::X-11441	0.01398	1	7072	T	C	http://mips.helmholtz-muenchen.de/proj/GWAS/gwas/gwas_server/shin_et_al.metal.out.tar.gz	NA	EUR
4277	30807572	2019	Gastrointestinal	Meconium ileus in cystic fibrosis	0.01415	1	6770	T	C	ftp://ftp.ebi.ac.uk/pub/databases/gwas/summary_statistics/GongJ_30807572_GCST007367/GongJ_prePMID_Meconium_Ileus.tsv.gz	NA	EUR
4670	BioRxiv: ht	2019	Neurological	Anterior limb of internal capsule axial diusivities	0.01475	1	17706	T	C	https://www.dropbox.com/s/8heofmigp5m7q85/ukbiobank_dti110_pheno67_txt.zip	NA	UKB2 (EUR)
4485	31676860	2019	Neurological	Right lateral orbitofrontal	0.01534	1	19629	T	C	https://www.med.unc.edu/bigs2/data/gwas-summary-statistics/	NA	UKB2 (EUR)
3396	31427789	2019	Activities	Other sociodemographic factors - Private healthcare	0.01543	1	128472	C	T	https://atlas.ctglab.nl/ukb2_sumstats/f.4674.0.0_res.EUR.sumstats.MACfilt.txt.gz	NA	UKB2 (EUR)
597	24816252	2014	Metabolic	Peptide::Dipeptide::pyroglutamylglycine	0.01612	1	1586	C	T	http://mips.helmholtz-muenchen.de/proj/GWAS/gwas/gwas_server/shin_et_al.metal.out.tar.gz	NA	EUR
63	28436984	2017	Reproduction	Age at menarche	0.017	1	252514	C	T	http://www.reprogen.org/Menarche_1KG_NatGen2017_WebsiteUpload.zip	ReproGen	EUR
3814	20045101	2010	Immunological	CD8+ T cell level	0.017	1	2538	NA	NA	https://genepi.qimr.edu.au/staff/manuelF/gwas_results/CD8.assoc.gz	NA	EUR

3218	31427789	2019	Activities	Time spent outdoors in winter	0.01796	1	304316	T	C	https://atlas.ctglab.nl/ukb2_sumstats/f.1060.0.0_res.EUR.sumstats.MACfilt.txt.gz	NA	UKB2 (EUR)
4208	31152163	2019	Metabolic	Blood urea nitrogen	0.01799	1	243031	C	T	http://ckdgen.imbi.uni-freiburg.de/files/Wuttke2019/BUN_overall_EA_YL_20171108_METAL1_nstud24.dbgap.txt.gz	CDKGen	EUR
845	24816252	2014	Metabolic	::::X-14658	0.01828	1	3533	C	T	http://mips.helmholtz-muenchen.de/proj/GWAS/gwas/gwas_server/shin_et_al.metal.out.tar.gz	NA	EUR
248	25772697	2015	Immunological	CD337 on NK early	0.0186	1	669	T	C	ftp://twinr-ftp.kcl.ac.uk/ImmuneCellScience/2-GWASResults/GWA_MFI_484.txt.gz	NA	EUR
4284	30804560	2019	Respiratory	FEV1/FVC ratio	0.0187	1	321047	C	T	ftp://ftp.ebi.ac.uk/pub/databases/gwas/summary_statistics/ShrineN_30804560_GCST007431/Shrine_30804560_UKBiobank_FEV1_to_FVC_RATIO.txt.gz	NA	UKB2 (EUR)
1189	23388002	2013	Cardiovascular	Aortic-valve calcification	0.02009	1	6942	NA	NA	https://grasp.nhlbi.nih.gov/downloads/ResultsOctober2016/Thanassoulis/AVC_meta_results_dbGAP_2013Jan08.txt	CHARGE	EUR
4215	31015462	2019	Metabolic	Estimated glomerular filtration rate	0.02092	1	350504	T	C	http://csg.sph.umich.edu/willer/public/eGFR2018/2018_Graham_et_al_eGFR_meta.tbl	NA	EUR+EAS
1119	27918534	2017	Metabolic	Pericardial adipose tissue volume (female)	0.021	1	6362	NA	NA	https://fox.nhlbi.nih.gov/CKDGen/PAT.tar.gz	CKDGen	EUR+AFR+EAS

3486	31427789	2019	Nutritional	Bread type: Brown	0.0211	1	372617	T	C	https://atlas.ctglab.nl/ukb2_sumstats/1448_2_logistic.EUR.sumstats.MACfilt.txt.gz	NA	UKB2 (EUR)
253	25772697	2015	Immunological	CD123 on 11c+123+DC	0.02179	1	669	T	C	ftp://twinr-ftp.kcl.ac.uk/ImmuneCellScience/2-GWASResults/GWA_MFI_58.txt.gz	NA	EUR
1122	27918534	2017	Metabolic	Pericardial adipose tissue volume (adjusted for height and weight, female)	0.022	1	6362	NA	NA	https://fox.nhlbi.nih.gov/CKDGen/PATadjHtWt.tar.gz	CKDGen	EUR+AFR+EAS
568	24816252	2014	Metabolic	Lipid::Sterol, Steroid::4-androsten-3beta,17beta-diol disulfate 1*	0.02212	1	7804	C	T	http://mips.helmholtz-muenchen.de/proj/GWAS/gwas/gwas_server/shin_et_al.metal.out.tar.gz	NA	EUR
3960	28990592	2017	Reproduction	Gestational weight gain (maternal, total)	0.02275	1	10555	T	C	http://egg-consortium.org/downloads/EGG-TotalGWG-Maternal.txt.gz	EGG	EUR
739	24816252	2014	Metabolic	::::X-11850	0.02382	1	4868	T	C	http://mips.helmholtz-muenchen.de/proj/GWAS/gwas/gwas_server/shin_et_al.metal.out.tar.gz	NA	EUR

3703	31427789	2019	Mortality	Diagnoses - secondary ICD10: Y83 Surgical operation and other surgical procedures as the cause of abnormal reaction of the patient, or later complication, without mention of misadventure at the time of the procedure	0.02498	1	244890	T	C	https://atlas.ctglab.nl/ukb2_sumstats/41204_Y83_logistic.EUR.sumstats.MACfilt.txt.gz	NA	UKB2 (EUR)
627	24816252	2014	Metabolic	Xenobiotics::Drug::p-acetamidophenylglucuronide	0.02559	1	1494	C	T	http://mips.helmholtz-muenchen.de/proj/GWAS/gwas/gwas_server/shin_et_al.xeno.metal.out.tar.gz	NA	EUR
743	24816252	2014	Metabolic	::::X-11876	0.02577	1	4965	C	T	http://mips.helmholtz-muenchen.de/proj/GWAS/gwas/gwas_server/shin_et_al.metal.out.tar.gz	NA	EUR
4207	31152163	2019	Metabolic	Blood urea nitrogen	0.02635	1	416178	C	T	http://ckdgen.imbi.uni-freiburg.de/files/Wuttke2019/BUN_overall_ALL_YL_20171017_METAL1_nstud_33.dbgap.txt.gz	CDKGen	EUR+EAS+AFR+AMR
242	25772697	2015	Immunological	PD1 on CD4mem	0.02639	1	669	T	C	ftp://twinr-ftp.kcl.ac.uk/ImmuneCellScience/2-GWASResults/GWA_MFI_236.txt.gz	NA	EUR

519	24816252	2014	Metabolic	Lipid::Long chain fatty acid::eicosenoate (20:1n9 or 11)	0.02682	1	7799	C	T	http://mips.helmholtz-muenchen.de/proj/GWAS/gwas/gwas_server/shin_et_al.metal.out.tar.gz	NA	EUR
147	26426971	2015	Metabolic	Body Mass Index (male > 50 yrs)	0.027	1	74324	C	T	http://portals.broadinstitute.org/collaboration/giant/images/7/7d/BMI.MEN.GT50.publicrelease.txt.gz	GIANT	EUR
4549	31676860	2019	Neurological	Left entorhinal	0.02782	1	21821	T	C	https://www.med.unc.edu/bigs2/data/gwas-summary-statistics/	NA	UKB2 (EUR meta)
4245	30980028	2019	Cognitive	Left handed	0.02883	1	331037	C	T	ftp://ftp.ebi.ac.uk/pub/databases/gwas/summary_statistics/deKovelCGF_30980028_GCST007592/DeKovel_PMID30980028_left_pval_maf001.assoc.gz	NA	UKB2 (EUR)
199	26631737	2016	Metabolic	Urinary albumin-to-creatinine ratio (non-diabetic)	0.029	1	46061	T	C	https://fox.nhlbi.nih.gov/CKDGen/formatted_Round3_UACR_noDM_2GC_b36_MAFget005_Nget50_2014_09_29.csv.gz	CKDGen	EUR
4048	29534260	2018	Skeletal	Plantar fasciitis	0.03049	1	102503	NA	NA	https://grasp.nhlbi.nih.gov/downloads/FullResults/2017/2017_Kim_Plantar_fasciitis/PlantarSummary.txt.zip	NA	EUR+AMR+EAS+AFR
849	24816252	2014	Metabolic	::::X-18601	0.03093	1	7663	C	T	http://mips.helmholtz-muenchen.de/proj/GWAS/gwas/gwas_server/shin_et_al.metal.out.tar.gz	NA	EUR
3596	31427789	2019	Activities	Mineral and other dietary supplements: Iron	0.03263	1	385261	T	C	https://atlas.ctglab.nl/ukb2_sumstats/6179_5_logistic.EUR.sumstats.MACfilt.txt.gz	NA	UKB2 (EUR)

3934	28878392	2017	Metabolic	Monosialylation	0.03316	1	2078	NA	NA	https://datashare.is.ed.ac.uk/bitstream/handle/10283/2738/5104819.zip?sequence=1&isAllowed=y	NA	EUR
518	24816252	2014	Metabolic	Lipid::Long chain fatty acid::dihomo-linoleate (20:2n6)	0.03364	1	7800	C	T	http://mips.helmholtz-muenchen.de/proj/GWAS/gwas/gwas_server/shin_et_al.metal.out.tar.gz	NA	EUR
1117	27918534	2017	Metabolic	Pericardial adipose tissue volume	0.034	1	12204	NA	NA	https://fox.nhlbi.nih.gov/CKDGen/PAT.tar.gz	CKDGen	EUR+AFR+EAS
3707	31427789	2019	Mortality	Diagnoses - secondary ICD10: Z72 Problems related to lifestyle	0.03411	1	244890	T	C	https://atlas.ctglab.nl/ukb2_sumstats/41204_Z72_logistic.EUR.sumstats.MACfilt.txt.gz	NA	UKB2 (EUR)
576	24816252	2014	Metabolic	Lipid::Sterol, Steroid::dehydroisoandrosterone sulfate (DHEA-S)	0.03449	1	7793	C	T	http://mips.helmholtz-muenchen.de/proj/GWAS/gwas/gwas_server/shin_et_al.metal.out.tar.gz	NA	EUR
4246	30980028	2019	Cognitive	Right handed	0.03463	1	331037	T	C	ftp://ftp.ebi.ac.uk/pub/databases/gwas/summary_statistics/deKovelCGF_30980028_GCST007594/DeKovel_PMID30980028_right_pval_maf001.assoc.gz	NA	UKB2 (EUR)
4303	30664634	2019	Metabolic	Legs-leg fat ratio (male)	0.03579	1	167431	C	T	https://myfiles.uu.se/ssf/s/readFile/share/3993/1270878243748486898/publicLink/GWAS_summary_stats_ratios.zip	NA	UKB2 (EUR)
3773	31427789	2019	Psychiatric	Anxiety - Recent worrying too much about different things	0.03688	1	126284	T	C	https://atlas.ctglab.nl/ukb2_sumstats/f.20520.0.0_res.EUR.sumstats.MACfilt.txt.gz	NA	UKB2 (EUR)

3286	31427789	2019	Psychiatric	Irritability	0.03707	1	369232	C	T	https://atlas.ctglab.nl/ukb2_sumstats/f.1940.0.0_logistic.EUR.sumstats.MACfilt.txt.gz	NA	UKB2 (EUR)
4610	31676860	2019	Neurological	Cerebellar vermal lobules VIII X	0.03729	1	21821	C	T	https://www.med.unc.edu/bigs2/data/gwas-summary-statistics/	NA	UKB2 (EUR meta)
220	25772697	2015	Immunological	T:%Vd1	0.03741	1	669	T	C	ftp://twinr-ftp.kcl.ac.uk/ImmuneCellScience/2-GWASResults/GWA_Lin_20.txt.gz	NA	EUR
839	24816252	2014	Metabolic	::::X-14473	0.0376	1	6884	C	T	http://mips.helmholtz-muenchen.de/proj/GWAS/gwas/gwas_server/shin_et_al.metal.out.tar.gz	NA	EUR
4066	30038396	2018	Environment	Educational attainment	0.0379	1	766345	C	T	https://www.dropbox.com/s/ho58e9jmytmpaf8/GWAS_EA_excl23andMe.txt?dl=1	SSGAC	UKB2 (EUR meta)
840	24816252	2014	Metabolic	::::X-14541	0.03844	1	1941	C	T	http://mips.helmholtz-muenchen.de/proj/GWAS/gwas/gwas_server/shin_et_al.metal.out.tar.gz	NA	EUR
3369	31427789	2019	Activities	Frequency of other exercises in last 4 weeks	0.03854	1	184631	T	C	https://atlas.ctglab.nl/ukb2_sumstats/f.3637.0.0_res.EUR.sumstats.MACfilt.txt.gz	NA	UKB2 (EUR)
4616	31676860	2019	Neurological	4th ventricle	0.03855	1	21821	C	T	https://www.med.unc.edu/bigs2/data/gwas-summary-statistics/	NA	UKB2 (EUR meta)
3781	31427789	2019	Psychiatric	Depression - Trouble falling asleep	0.03937	1	48926	C	T	https://atlas.ctglab.nl/ukb2_sumstats/f.20533.0.0_logistic.EUR.sumstats.MACfilt.txt.gz	NA	UKB2 (EUR)

4305	30664634	2019	Metabolic	Trunk-trunk fat ratio (male)	0.03982	1	167431	T	C	https://myfiles.uu.se/ssf/s/readFile/share/3993/1270878243748486898/publicLink/GWAS_summary_stats_ratios.zip	NA	UKB2 (EUR)
2053	24084763	2013	Neoplasms	Advanced adenoma	0.04051	1	1406	A	G	ftp://ftp.ebi.ac.uk/pub/databases/gwas/summary_statistics/WangJ_24084763_GCST002212/adv_adno_assoc.assoc.txt.zip	NA	EUR
696	24816252	2014	Metabolic	::::X-11374	0.04087	1	2609	T	C	http://mips.helmholtz-muenchen.de/proj/GWAS/gwas/gwas_server/shin_et_al.metal.out.tar.gz	NA	EUR
3426	31427789	2019	Cognitive	Fluid intelligence test - FI3 : word interpolation	0.04177	1	99362	C	T	https://atlas.ctglab.nl/ukb2_sumstats/f.20169.0.0_logistic.EUR.sumstats.MACfilt.txt.gz	NA	UKB2 (EUR)
3310	31427789	2019	Activities	Age started wearing glasses or contact lenses	0.04272	1	332074	T	C	https://atlas.ctglab.nl/ukb2_sumstats/f.2217.0.0_res.EUR.sumstats.MACfilt.txt.gz	NA	UKB2 (EUR)
86	26367794	2015	Skeletal	Lumbar Spine BMD	0.04311	1	32965	T	C	http://www.gefos.org/sites/default/files/ls2stu.MAF0_.005.pos_.out_.gz	GEFOS	EUR
1219	25607358	2015	Neurological	Mean Accumbens	0.0443	1	13171	T	C	http://enigma.ini.usc.edu/research/download-enigma-gwas-results/	ENIGMA	EUR
520	24816252	2014	Metabolic	Lipid::Long chain fatty acid::margarate (17:0)	0.0445	1	7796	C	T	http://mips.helmholtz-muenchen.de/proj/GWAS/gwas/gwas_server/shin_et_al.metal.out.tar.gz	NA	EUR
4623	31676860	2019	Neurological	Left caudate	0.04547	1	21821	C	T	https://www.med.unc.edu/bigs2/data/gwas-summary-statistics/	NA	UKB2 (EUR meta)

997	27989323	2017	Immunological	Interferon gamma-induced protein 10 (CXCL10)	0.04614	1	3685	T	C	http://www.computationalmedicine.fi/data#Cytokine_GWAS	NA	EUR
515	24816252	2014	Metabolic	Lipid::Long chain fatty acid::10-nonadecenoate (19:1n9)	0.04803	1	7789	C	T	http://mips.helmholtz-muenchen.de/proj/GWAS/gwas/gwas_server/shin_et_al.metal.out.tar.gz	NA	EUR
348	25772697	2015	Immunological	CD8:%R5+	0.04822	1	669	C	T	ftp://twinr-ftp.kcl.ac.uk/ImmuneCellScience/2-GWASResults/GWA_P5_CD8_165.txt.gz	NA	EUR
4033	30482948	2018	Psychiatric	Alcohol dependency (unrelated genotyped individuals)	0.04827	1	28757	T	C	https://www.med.unc.edu/pgc/results-and-downloads/downloads	PGC	EUR
4461	31676860	2019	Neurological	Left pars orbitalis	0.04925	1	19629	T	C	https://www.med.unc.edu/bigs2/data/gwas-summary-statistics/	NA	UKB2 (EUR)
217	25772697	2015	Immunological	NKT:%4+R5-	0.04968	1	669	T	C	ftp://twinr-ftp.kcl.ac.uk/ImmuneCellScience/2-GWASResults/GWA_Lin_16.txt.gz	NA	EUR
3697	31427789	2019	Gastrointestinal	Diagnoses - secondary ICD10: K29 Gastritis and duodenitis	0.04986	1	244890	C	T	https://atlas.ctglab.nl/ukb2_sumstats/41204_K29_logistic.EUR.sumstats.MACfilt.txt.gz	NA	UKB2 (EUR)

Supplementary Table 10. Full table of PheWAS associations between discovery lead sequence variant, rs12776883, and phenotypes in the Copenhagen Hospital Biobank Cardiovascular Disease Cohort. Associations are sorted by p-value and adjusted as described in the Methods section. Only associations with an unadjusted p-value<0.05 are shown.

phecode	phenotype	phenotype-category	p-value	p-adj	log-odds	standard-error
458.9	Hypotension NOS	circulatory system	0.000649	0.0506152	-0.14155	0.0415057
618.2	Uterine/Uterovaginal prolapse	genitourinary	0.00089	0.0687493	0.183922	0.055345
198.2	Secondary malignancy of respiratory organs	neoplasms	0.001369	0.1037796	-0.16376	0.0511571
38.2	Gram positive septicemia	infectious diseases	0.001647	0.1235669	-0.16646	0.0528895
364.4	Corneal degenerations	sense organs	0.001692	0.1266904	-0.61085	0.194563
312	Conduct disorders	mental disorders	0.00195	0.144542	0.538026	0.17368
798	Malaise and fatigue	symptoms	0.00248	0.1801822	-0.44653	0.147578
527.7	Disturbance of salivary secretion	digestive	0.003744	0.2592242	-0.42082	0.145161
454	Varicose veins	circulatory system	0.004257	0.289171	0.227238	0.0794975
70	Viral hepatitis	infectious diseases	0.005463	0.3548271	-0.24022	0.0864606
614.54	Abscess or ulceration of vulva	genitourinary	0.005828	0.3735122	-0.18877	0.0684614
521.4	Tooth complications likely association with other diseases	digestive	0.006147	0.3893552	0.577628	0.210824
38.1	Gram negative septicemia	infectious diseases	0.007625	0.4579255	-0.11787	0.0441758
159	Malignant neoplasm of other and ill-defined sites within the digestive organs and peritoneum	neoplasms	0.008195	0.4822707	-0.19872	0.0751588
601.3	Orchitis and epididymitis	genitourinary	0.008389	0.4903169	0.484586	0.183834
686	Other local infections of skin and subcutaneous tissue	dermatologic	0.010061	0.5546764	-0.06769	0.0262985
365.2	Primary angle-closure glaucoma	sense organs	0.011396	0.6002389	0.239145	0.0945114
613	Other nonmalignant breast conditions	genitourinary	0.011893	0.6160104	0.251758	0.100091
324	Other CNS infection and poliomyelitis	neurological	0.012021	0.6199617	0.352741	0.140448
578.8	Hemorrhage of rectum and anus	digestive	0.012363	0.6303404	-0.07003	0.0279933
614.32	Chronic inflammatory pelvic disease	genitourinary	0.012914	0.6464997	0.263763	0.106094
276.42	Alkalosis	endocrine/metabolic	0.013091	0.6515249	-0.51759	0.208595
133	Arthropod-borne diseases	infectious diseases	0.015094	0.7037896	-0.39003	0.160496
442.3	Aneurysm of artery of lower extremity	circulatory system	0.015105	0.7040656	0.227314	0.0935507

701.5	Abnormal granulation tissue	dermatologic	0.015556	0.7147152	0.370131	0.152999
747.1	Cardiac congenital anomalies	congenital anomalies	0.0166	0.7379322	-0.20095	0.0838883
285.22	Anemia in neoplastic disease	hematopoietic	0.017681	0.7599988	-0.11725	0.0494243
772	Symptoms of the muscles	symptoms	0.018016	0.7664737	-0.21602	0.09133
580.2	Nephrotic syndrome without mention of glomerulonephritis	genitourinary	0.019087	0.7859816	-0.21006	0.0896239
38	Septicemia	infectious diseases	0.019308	0.7898051	-0.05166	0.0220808
603.2	Spermatocele	genitourinary	0.019541	0.7937634	0.138118	0.0591497
963	Poisoning by primarily systemic agents	injuries & poisonings	0.020166	0.8040179	-0.22582	0.0971993
228	Hemangioma and lymphangioma, any site	neoplasms	0.020456	0.8086183	0.15191	0.0655389
229	Benign neoplasm of unspecified sites	neoplasms	0.021194	0.8198154	0.164327	0.0713069
740.9	Osteoarthrosis NOS	musculoskeletal	0.021753	0.8278605	0.064326	0.0280331
350.2	Abnormality of gait	neurological	0.024005	0.8568501	0.200031	0.088625
465.4	Acute laryngitis and tracheitis	respiratory	0.024137	0.8583897	-0.18037	0.0799868
473.4	Voice disturbance	respiratory	0.02446	0.8620888	0.108173	0.0480804
634.3	Ectopic pregnancy	pregnancy complications	0.024831	0.8662237	0.119562	0.0532802
1005	Other symptoms	Unknown	0.025521	0.8735803	-0.04583	0.0205181
593	Hematuria	genitourinary	0.025534	0.8737205	0.306388	0.137195
625.1	Dyspareunia	genitourinary	0.026488	0.8832376	0.132712	0.0598076
427.3	Other specified cardiac dysrhythmias	circulatory system	0.027166	0.8895693	-0.0919	0.0416023
315.3	Mental retardation	mental disorders	0.027544	0.8929492	-0.21525	0.097674
374.1	Ectropion or entropion	sense organs	0.028019	0.897055	0.143112	0.0651392
320	Meningitis	neurological	0.029187	0.9064927	0.120583	0.0552889
618.1	Prolapse of vaginal walls	genitourinary	0.029767	0.9108603	0.07213	0.0331909
165.1	Cancer of bronchus; lung	neoplasms	0.029909	0.9118946	-0.06716	0.0309311
295.1	Schizophrenia	mental disorders	0.030539	0.9163599	0.135615	0.0626971
646	Other complications of pregnancy NEC	pregnancy complications	0.031194	0.9207593	0.066871	0.0310365
966	Poisoning by anticonvulsants and anti-Parkinsonism drugs	injuries & poisonings	0.03207	0.9262922	-0.20683	0.0964902
290.1	Dementias	mental disorders	0.032075	0.9263237	-0.14461	0.0674642
755.61	Congenital hip dysplasia and deformity	congenital anomalies	0.032626	0.9296059	-0.15601	0.0730181
214.1	Lipoma of skin and subcutaneous tissue	neoplasms	0.03359	0.935	0.085647	0.0403052
285	Other anemias	hematopoietic	0.033695	0.9355645	-0.04033	0.0189901

1015	Effects of other external causes	Unknown	0.03387	0.9364915	0.078297	0.0369045
433	Cerebrovascular disease	circulatory system	0.03464	0.9404149	0.129566	0.0613317
994.2	Sepsis	injuries & poisonings	0.035613	0.9450328	-0.04792	0.0228038
451.2	Phlebitis and thrombophlebitis of lower extremities	circulatory system	0.035796	0.9458625	-0.05786	0.0275635
512.7	Shortness of breath	respiratory	0.036034	0.9469183	-0.03409	0.0162597
303.4	Somatoform disorder	mental disorders	0.037255	0.9520394	0.163923	0.0786971
722.9	Other and unspecified disc disorder	musculoskeletal	0.037267	0.9520838	0.074655	0.035843
187.8	Neoplasm of uncertain behavior of male genital organs	neoplasms	0.037911	0.9545844	0.386172	0.186034
272.12	Hyperglyceridemia	endocrine/metabolic	0.039518	0.9602697	-0.26931	0.130814
355	Complex regional/central pain syndrome	neurological	0.040584	0.9636471	-0.39726	0.193996
250.13	Type 1 diabetes with ophthalmic manifestations	endocrine/metabolic	0.041014	0.9649257	0.130954	0.0640867
756.5	Congenital osteodystrophies	congenital anomalies	0.041229	0.9655491	-0.26418	0.129422
674	Other complications of the puerperium NEC	pregnancy complications	0.041237	0.9655736	0.131889	0.0646156
569.1	Toxic gastroenteritis and colitis	digestive	0.04164	0.9667113	-0.30076	0.147642
773	Pain in limb	symptoms	0.042416	0.9688004	-0.03687	0.0181681
716.8	Palindromic rheumatism	musculoskeletal	0.043909	0.9724608	0.318456	0.158045
524.3	Anomalies of tooth position/malocclusion	digestive	0.044548	0.9738962	0.152205	0.0757654
509.5	Respiratory arrest	respiratory	0.044672	0.9741665	-0.18834	0.0938059
151	Cancer of stomach	neoplasms	0.046523	0.9778788	-0.12058	0.0605762
626.12	Excessive or frequent menstruation	genitourinary	0.046643	0.9781007	0.07988	0.0401504
370	Keratitis	sense organs	0.046862	0.9784986	0.115226	0.0579742
602	Other disorders of prostate	genitourinary	0.047971	0.9804111	-0.34791	0.175924
519.2	Respiratory complications	respiratory	0.048407	0.9811161	-0.28176	0.142753
250.21	Type 2 diabetes with ketoacidosis	endocrine/metabolic	0.048472	0.9812185	-0.25669	0.130088
597.1	Urethral stricture (not specified as infectious)	genitourinary	0.048556	0.9813504	-0.11098	0.0562626
627	Menopausal and postmenopausal disorders	genitourinary	0.048985	0.9820112	0.293956	0.149313
204	Leukemia	neoplasms	0.049864	0.9832949	-0.26093	0.133049

Supplementary Table 11. Full table of PheWAS associations between the meta-analysis lead sequence variant, rs71543507, and phenotypes found in the GWAS ATLAS resource. (https://atlas.ctglab.nl/). Associations are sorted by Bonferroni-corrected p-value. Only associations with an unadjusted p-value<0.05 are shown.

atlas ID	PMID	Year	Domain	Trait	P-value	P-adj	N	EA	NEA	File	Consortium	Population
3751	31427789	2019	Psychiatric	Traumatic events - Avoided activities or situations because of previous stressful experience in past month	0.00199	1	126606	G	A	https://atlas.ctglab.nl/ukb2_sumstats/f.20495.0.0_res.EUR.sumstats.MACfilt.txt.gz	NA	UKB2 (EUR)
4361	30061737	2018	Cardiovascular	Atrial fibrillation	0.00211	1	1030836	G	A	http://csg.sph.umich.edu/willer/public/afib2018/nielsen-thorolfsdottir-willer-NG2018-AFib-gwas-summary-statistics.tbl.gz	NA	EUR
3752	31427789	2019	Psychiatric	Traumatic events - Felt distant from other people in past month	0.00377	1	57017	G	A	https://atlas.ctglab.nl/ukb2_sumstats/f.20496.0.0_res.EUR.sumstats.MACfilt.txt.gz	NA	UKB2 (EUR)
4485	31676860	2019	Neurological	Right lateral orbitofrontal	0.00402	1	19629	A	G	https://www.med.unc.edu/bigs2/data/gwas-summary-statistics/	NA	UKB2 (EUR)
4498	31676860	2019	Neurological	Right precuneus	0.00414	1	19629	A	G	https://www.med.unc.edu/bigs2/data/gwas-summary-statistics/	NA	UKB2 (EUR)
4586	31676860	2019	Neurological	Right lateral orbitofrontal	0.00438	1	21821	A	G	https://www.med.unc.edu/bigs2/data/gwas-summary-statistics/	NA	UKB2 (EUR meta)

4599	31676860	2019	Neurological	Right precuneus	0.00446	1	21821	A	G	https://www.med.unc.edu/bigs2/data/gwas-summary-statistics/	NA	UKB2 (EUR meta)
4268	29892015	2018	Cardiovascular	Atrial Fibrillation	0.00508	1	588190	G	A	https://personal.broadinstitute.org/mvon/AF_HRC_GWAS_ALLv11.zip	NA	EUR+AFR+EAS+AMR
4362	29899525	2018	Activities	Acceleration average	0.0055	1	91084	A	G	ftp://ftp.ebi.ac.uk/pub/databases/gwas/summary_statistics/KlimentidisYC_29899525_GCST006099/Klimentidis_29899525_AccAve_Model1_BOLTLMM_500K.txt.gz	NA	UKB2 (EUR)
4041	29906448	2018	Psychiatric	Schizophrenia vs Bipolar disorder	0.00562	1	38855	G	A	https://www.med.unc.edu/pgc/results-and-downloads/downloads	PGC	EUR
3254	31427789	2019	Nutritional	Salt added to food	0.00782	1	386322	G	A	https://atlas.ctglab.nl/ukb2_sumstats/f.1478.0.0_res.EUR.sumstats.MACfilt.txt.gz	NA	UKB2 (EUR)
3563	31427789	2019	Activities	Vitamin and mineral supplements: Vitamin C	0.00787	1	384452	A	G	https://atlas.ctglab.nl/ukb2_sumstats/6155_3_logistic.EUR.sumstats.MACfilt.txt.gz	NA	UKB2 (EUR)
3404	31427789	2019	Cognitive	Fluid intelligence test - FI6 : conditional arithmetic	0.00843	1	96994	G	A	https://atlas.ctglab.nl/ukb2_sumstats/f.4990.0.0_logistic.EUR.sumstats.MACfilt.txt.gz	NA	UKB2 (EUR)

3422	31427789	2019	Cognitive	Trail making test - Duration to complete numeric path (trail #1)	0.01004	1	84261	G	A	https://atlas.ctglab.nl/ukb2_sumstats/f.20156.0.0_res.EUR.sumstats.MACfilt.txt.gz	NA	UKB2 (EUR)
1198	25027320	2014	Psychiatric	Phosphorylated Tau at position 181	0.0101	1	363	G	A	https://grasp.nhlbi.nih.gov/downloads/ResultsOctober2016/Ramirez/MA_pTau181_Short.zip	NA	EUR
4415	30053915	2018	Immunological	Varicella Zoster Virus seropositivity	0.0108	1	1000	A	G	https://zenodo.org/record/1217136/files/GWAS_Serostatus.tar.gz	Milieu Int?©	EUR
4335	30531941	2018	Activities	Overall activity	0.011	1	91105	A	G	https://ora.ox.ac.uk/objects/uuid:ff479f44-bf35-48b9-9e67-e690a2937b22/download_file?file_format=gzip&safe_filename=Doherty-2018-NatureComms-overall-activity.csv.gz&type_of_work=Dataset	NA	UKB2 (EUR)
1135	26482879	2015	Dermatological	Eczema	0.01184	1	40835	G	A	https://data.bris.ac.uk/datasets/28uchsdpmub118uex26ylacqm/EAGLE_AD_GWAS_results_2015.txt.gz	EAGLE	EUR
4386	30898391	2019	Neoplasms	Epithelial ovarian cancer (Borderline serous and mucinous)	0.0139	1	4340	G	A	ftp://ftp.ebi.ac.uk/pub/databases/gwas/summary_statistics/LawrensonK_30898391_GCST007728	NA	EAS

4339	30531941	2018	Activities	Walking	0.014	1	91105	A	G	https://ora.ox.ac.uk/objects/uuid:ff479f44-bf35-48b9-9e67-e690a2937b22/download_file?file_format=gzip&safe_filename=Doherty-2018-NatureComms-walking.csv.gz&type_of_work=Dataset	NA	UKB2 (EUR)
3371	31427789	2019	Respiratory	Chest pain or discomfort when walking uphill or hurrying	0.01796	1	48190	G	A	https://atlas.ctglab.nl/ukb2_sumstats/f.3751.0.0_logistic.EUR.sumstats.MACfilt.txt.gz	NA	UKB2 (EUR)
4239	30995994	2019	Cognitive	Latent naming speed	0.01832	1	441	A	G	ftp://ftp.ebi.ac.uk/pub/databases/gwas/summary_statistics/TruongDT_30995994_GCST007862/TruongDT_prePMID_GRaDAA_LatentNamingSpeedGWAS_SummaryStats.txt	NA	AFR
3778	31427789	2019	Psychiatric	Traumatic events - Victim of physically violent crime	0.02044	1	126580	G	A	https://atlas.ctglab.nl/ukb2_sumstats/f.20529.0.0_logistic.EUR.sumstats.MACfilt.txt.gz	NA	UKB2 (EUR)
4098	29403010	2018	Metabolic	Total cholesterol	0.0209	1	128305	A	G	http://jenger.riken.jp/en/result	NA	EAS
3766	31427789	2019	Psychiatric	Depression - Recent thoughts of suicide or self-harm	0.02129	1	125844	G	A	https://atlas.ctglab.nl/ukb2_sumstats/f.20513.0.0_res.EUR.sumstats.MACfilt.txt.gz	NA	UKB2 (EUR)

862	27005778	2016	Metabolic	Creatinine	0.02229	1	24810	A	G	http://www.computationalmedicine.fi/data/NMR_GWAS/	NA	EUR
3439	31427789	2019	Psychiatric	Tobacco smoking	0.02309	1	97172	G	A	https://atlas.ctglab.nl/ukb2_sumstats/f.22506.0.0_res.EUR.sumstats.MACfilt.txt.gz	NA	UKB2 (EUR)
3427	31427789	2019	Cognitive	Fluid intelligence test - FI4 : positional arithmetic	0.02386	1	98875	A	G	https://atlas.ctglab.nl/ukb2_sumstats/f.20171.0.0_logistic.EUR.sumstats.MACfilt.txt.gz	NA	UKB2 (EUR)
3234	31427789	2019	Psychiatric	Daytime dozing / sleeping (narcolepsy)	0.02457	1	384879	G	A	https://atlas.ctglab.nl/ukb2_sumstats/f.1220.0.0_res.EUR.sumstats.MACfilt.txt.gz	NA	UKB2 (EUR)
3274	31427789	2019	Dermatological	Ease of skin tanning	0.02545	1	378364	G	A	https://atlas.ctglab.nl/ukb2_sumstats/f.1727.0.0_res.EUR.sumstats.MACfilt.txt.gz	NA	UKB2 (EUR)
3670	31427789	2019	Gastrointestinal	Diagnoses - main ICD10: K29 Gastritis and duodenitis	0.02621	1	300791	G	A	https://atlas.ctglab.nl/ukb2_sumstats/41202_K29_logistic.EUR.sumstats.MACfilt.txt.gz	NA	UKB2 (EUR)
3750	31427789	2019	Psychiatric	Traumatic events - Felt irritable or had angry outbursts in past month	0.02639	1	57010	G	A	https://atlas.ctglab.nl/ukb2_sumstats/f.20494.0.0_res.EUR.sumstats.MACfilt.txt.gz	NA	UKB2 (EUR)
4316	30643251	2019	Psychiatric	Smoking cessation	0.0275	1	312821	G	A	https://conservancy.umn.edu/handle/11299/201564	NA	UKB2 (EUR meta)

3951	28931804	2017	Neurological	Amyotrophic lateral sclerosis (LMM)	0.02889	1	4084	G	A	http://cnsgenomics.com/data/benyamin_et_al_2017_nc/BenyaminEtAl_NatComm_Data.zip	NA	EAS
3950	28931804	2017	Neurological	Amyotrophic lateral sclerosis (logistic)	0.02896	1	4084	G	A	http://cnsgenomics.com/data/benyamin_et_al_2017_nc/BenyaminEtAl_NatComm_Data.zip	NA	EAS
40	27046643	2016	Cognitive	Reaction time	0.02901	1	111483	G	A	http://www.psy.ed.ac.uk/ccace/downloads/Davies_MP_2016.zip	CCACE	UKB1 (EUR)
3768	31427789	2019	Psychiatric	Anxiety - Recent trouble relaxing	0.0292	1	126422	G	A	https://atlas.ctglab.nl/ukb2_sumstats/f.20515.0.0_res.EUR.sumstats.MACfilt.txt.gz	NA	UKB2 (EUR)
3241	31427789	2019	Nutritional	Salad / raw vegetable intake	0.02923	1	363780	G	A	https://atlas.ctglab.nl/ukb2_sumstats/f.1299.0.0_res.EUR.sumstats.MACfilt.txt.gz	NA	UKB2 (EUR)
3488	31427789	2019	Nutritional	Cereal type: Bran cereal (e.g. All Bran, Branflakes)	0.03004	1	319477	A	G	https://atlas.ctglab.nl/ukb2_sumstats/1468_1_logistic.EUR.sumstats.MACfilt.txt.gz	NA	UKB2 (EUR)
4607	31676860	2019	Neurological	Right insula	0.03067	1	21821	A	G	https://www.med.unc.edu/bigs2/data/gwas-summary-statistics/	NA	UKB2 (EUR meta)
4413	30053915	2018	Cell	Total IgE levels	0.03141	1	1000	A	G	https://zenodo.org/record/1217136/files/GWAS_TotalIg_levels.tar.gz	Milieu Int?©ı	EUR

4241	30995994	2019	Cognitive	Rapid alternating stimulus test for letters/numbers	0.03147	1	1324	A	G	ftp://ftp.ebi.ac.uk/pub/databases/gwas/summary_statistics/TruongDT_30995994_GCST007863/TruongDT_prePMID_RanLettNum_SummaryStats.txt	NA	AMR+AFR
4528	31676860	2019	Neurological	Left ventral DC	0.03169	1	19629	G	A	https://www.med.unc.edu/bigs2/data/gwas-summary-statistics/	NA	UKB2 (EUR)
4629	31676860	2019	Neurological	Left ventral DC	0.03248	1	21821	G	A	https://www.med.unc.edu/bigs2/data/gwas-summary-statistics/	NA	UKB2 (EUR meta)
4243	30995994	2019	Cognitive	Rapid automised naming of objects	0.03331	1	1324	A	G	ftp://ftp.ebi.ac.uk/pub/databases/gwas/summary_statistics/TruongDT_30995994_GCST007865/TruongDT_prePMID_RanObj_SummaryStats.txt	NA	AMR+AFR
1136	27015805	2016	Mortality	Parents' age at death	0.036	1	75244	G	A	http://www.t2diabetesgenes.org/datasets/results.UKBiobank_9millionSNPs.parents_lifespan.Pilling_et_al_2016_Aging.txt.gz	T2D-GENES	UKB1 (EUR)
46	28196072	2017	Dermatological	Baldness	0.03703	1	52874	G	A	http://www.psy.ed.ac.uk/ccace/downloads/Hagenaars2017_UKB_MPB_summary_results.zip	CCACE	UKB1 (EUR)
4112	29403010	2018	Metabolic	Sodium	0.03765	1	127304	A	G	http://jenger.riken.jp/en/result	NA	EAS

3196	31427789	2019	Environment	Time employed in main current job	0.038	1	208904	G	A	https://atlas.ctglab.nl/ukb2_sumstats/f.757.0.0_res.EUR.sumstats.MACfilt.txt.gz	NA	UKB2 (EUR)
3740	31427789	2019	Psychiatric	Depression - Depression possibly related to stressful or traumatic event	0.03829	1	71568	G	A	https://atlas.ctglab.nl/ukb2_sumstats/f.20447.0.0_logistic.EUR.sumstats.MACfilt.txt.gz	NA	UKB2 (EUR)
4732	BioRxiv: ht	2019	Neurological	Superior longitudinal fasciculus mode of anisotropy	0.03888	1	17706	G	A	https://www.dropbox.com/s/cg4dbfhtxx3686y/ukbiobank_dti110_pheno370_txt.zip	NA	UKB2 (EUR)
4340	30531941	2018	Activities	Walking (conditioning sex and BMI)	0.04	1	91105	A	G	https://ora.ox.ac.uk/objects/uuid:ff479f44-bf35-48b9-9e67-e690a2937b22/download_file?file_format=gzip&safe_filename=Doherty-2018-NatureComms-walking-sexBMI.csv.gz&type_of_work=Dataset	NA	UKB2 (EUR)
3971	29304378	2018	Skeletal	Total body BMD	0.04052	1	66628	A	G	http://www.gefos.org/sites/default/files/METAANALYSIS2016_all_GEFOS.txt.gz	GEFOS	EUR+AFR+Others
4188	31217584	2019	Metabolic	Fasting insulin	0.04064	1	21551	G	A	ftp://ftp.ebi.ac.uk/pub/databases/gwas/summary_statistics/WojcikGL_31217584_GCST008033/WojcikG_PMID_lnFI.adjBMI.invn.gz	NA	AMR+AFR+EAS+Others

4336	30531941	2018	Activities	Overall activity (conditioning sex and BMI)	0.041	1	91105	A	G	https://ora.ox.ac.uk/objects/uuid:ff479f44-bf35-48b9-9e67-e690a2937b22/download_file?file_format=gzip&safe_filename=Doherty-2018-NatureComms-overall-activity-sexBMI.csv.gz&type_of_work=Dataset	NA	UKB2 (EUR)
43	27818178	2016	Environment	Social deprivation	0.04128	1	112151	A	G	http://www.psy.ed.ac.uk/ccace/downloads/Hill_CB_2016.zip	CCACE	UKB1 (EUR)
4465	31676860	2019	Neurological	Left posterior cingulate	0.04187	1	19629	A	G	https://www.med.unc.edu/bigs2/data/gwas-summary-statistics/	NA	UKB2 (EUR)
4576	31676860	2019	Neurological	Left insula	0.04295	1	21821	A	G	https://www.med.unc.edu/bigs2/data/gwas-summary-statistics/	NA	UKB2 (EUR meta)
3279	31427789	2019	Mortality	Father's age at death	0.04565	1	283990	G	A	https://atlas.ctglab.nl/ukb2_sumstats/f.1807.0.0_res.EUR.sumstats.MACfilt.txt.gz	NA	UKB2 (EUR)
4727	BioRxiv: ht	2019	Neurological	Posterior thalamic radiation (include optic radiation) mode of anisotropy	0.04657	1	17706	G	A	https://www.dropbox.com/s/93jfxk23u77dq9q/ukbiobank_dti110_pheno357_txt.zip	NA	UKB2 (EUR)

3839	27863252	2016	Immunological	Eosinophil percentage of white cells (two-way meta)	0.0482	1	132052	G	A	ftp://ftp.sanger.ac.uk/pub/project/humgen/summary_statistics/human/2017-12-12/hematological_traits/eo_p/eo_p_N172378_ukbb_ukbil_meta.tsv.gz	NA	UKB1 (EUR meta)
4475	31676860	2019	Neurological	Left insula	0.04844	1	19629	A	G	https://www.med.unc.edu/bigs2/data/gwas-summary-statistics/	NA	UKB2 (EUR)
4539	31676860	2019	Neurological	Right amygdala	0.04853	1	19629	A	G	https://www.med.unc.edu/bigs2/data/gwas-summary-statistics/	NA	UKB2 (EUR)
4175	30048462	2018	Skeletal	Heel bone mineral density	0.049	1	394929	A	G	https://grasp.nhlbi.nih.gov/downloads/ResultsMarch2019/2018_Kim/BMD_v3_SumStats.txt.zip	NA	UKB2 (EUR)
3225	31427789	2019	Activities	Hands-free device/speakerphone use with mobile phone in last 3 month	0.04958	1	324453	G	A	https://atlas.ctglab.nl/ukb2_sumstats/f.1130.0.0_res.EUR.sumstats.MACfilt.txt.gz	NA	UKB2 (EUR)

Supplementary Table 12. Full table of PheWAS associations between the meta-analysis lead sequence variant, rs71543507, and phenotypes in the Copenhagen Hospital Biobank Cardiovascular Disease Cohort. Associations are sorted by p-value and adjusted as described in the Methods section. Only associations with an unadjusted p-value<0.05 are shown.

phecode	phenotype	phenotype-category	p-value	p-adj	log-odds	standard-error
721.2	Spondylosis with myelopathy	musculoskeletal	0.000111828	0.004463374	0.00158638	0.00041062
10	Tuberculosis	infectious diseases	0.000388436	0.015420347	0.00142414	0.000401412
527.1	Hypertrophy of salivary gland	digestive	0.001029817	0.040376155	0.000480217	0.000146307
639	Complications following abortion or ectopic and molar pregnancies	pregnancy complications	0.001252016	0.048877137	0.00104196	0.000322913
634	Miscarriage; stillbirth	pregnancy complications	0.001261798	0.049249692	0.00625829	0.00194084
386.21	Central origin vertigo	sense organs	0.00195326	0.075226831	0.000826599	0.000266882
523.1	Gingivitis	digestive	0.002223566	0.085192544	0.000743042	0.000242934
735	Acquired foot deformities	musculoskeletal	0.003087877	0.116360562	0.00144592	0.000488675
242.2	Toxic multinodular goiter	endocrine/metabolic	0.00335112	0.125645964	0.00170003	0.000579515
374	Other disorders of eyelids	sense organs	0.004052938	0.149938816	0.00110369	0.000384025
280.1	Iron deficiency anemias, unspecified or not due to blood loss	hematopoietic	0.005277157	0.190748237	0.00311813	0.00111777
871.3	Open wound of foot except toe(s) alone	injuries & poisonings	0.006973969	0.244167904	0.00379958	0.00140825
198.7	Secondary malignant neoplasm of skin	neoplasms	0.007060411	0.246795236	-0.00092497	0.000343346
535.6	Duodenitis	digestive	0.007530087	0.260915672	0.00112891	0.000422425
647.1	Infections of genitourinary tract during pregnancy	pregnancy complications	0.008029713	0.275653184	0.00160215	0.000604399
366.2	Senile cataract	sense organs	0.008241002	0.281799037	-0.0057525	0.0021773
386.3	Labyrinthitis	sense organs	0.008772432	0.29703308	-0.0007253	0.000276749
433.3	Cerebral ischemia	circulatory system	0.00890738	0.300851093	-0.00043247	0.000165342
204.11	Lymphoid leukemia, acute	neoplasms	0.009981826	0.330536841	-0.00036596	0.000142038
965.3	Salicylates causing adverse effects in therapeutic use	injuries & poisonings	0.009985044	0.330623885	0.00141228	0.000548173

433.8	Late effects of cerebrovascular disease	circulatory system	0.010662769	0.348710442	-0.00463535	0.00181525
250.41	Impaired fasting glucose	endocrine/metabolic	0.010791703	0.352096975	-0.00062141	0.000243748
300	Anxiety, phobic and dissociative disorders	mental disorders	0.011309932	0.365536191	-0.00136316	0.000538168
339	Other headache syndromes	neurological	0.011878183	0.379960242	-0.0014364	0.000570965
446.9	Arteritis NOS	circulatory system	0.012192422	0.387798859	0.00052601	0.000209856
198.5	Secondary malignancy of brain/spine	neoplasms	0.012724479	0.4008511	-0.00114163	0.000458232
250.42	Other abnormal glucose	endocrine/metabolic	0.015963937	0.47465985	0.00142887	0.000592957
722.8	Postlaminectomy syndrome	musculoskeletal	0.016389662	0.483674727	0.000409292	0.00017053
1007	Injury to blood vessels	Unknown	0.018916901	0.534165379	-0.00125355	0.00053407
440.1	Atherosclerosis of renal artery	circulatory system	0.02072717	0.567338538	-0.0005449	0.000235592
187.2	Malignant neoplasm of testis	neoplasms	0.020738627	0.567540976	0.000806762	0.000348839
261	Vitamin deficiency	endocrine/metabolic	0.020742448	0.56760846	-0.00054778	0.000236863
253.3	Diabetes insipidus	endocrine/metabolic	0.021703511	0.584261933	-0.0004211	0.000183443
465	Acute upper respiratory infections of multiple or unspecified sites	respiratory	0.02214624	0.591721601	0.00237411	0.0010377
732.1	Juvenile osteochondrosis	musculoskeletal	0.0221544	0.591857867	-0.00126603	0.000553408
360.2	Progressive myopia	sense organs	0.023685361	0.61665301	0.000881418	0.000389629
440	Atherosclerosis	circulatory system	0.02394749	0.620748502	-0.00237234	0.00105065
586	Other disorders of the kidney and ureters	genitourinary	0.023948593	0.620765642	0.00086145	0.000381519
426.24	Atrioventricular block, complete	circulatory system	0.024066337	0.622591279	0.00206121	0.000913629
251.1	Hypoglycemia	endocrine/metabolic	0.024647337	0.631474991	0.00200781	0.000893596
634.3	Ectopic pregnancy	pregnancy complications	0.026653239	0.660606505	0.00165294	0.000745721
433.1	Occlusion and stenosis of precerebral arteries	circulatory system	0.027489067	0.672071098	-0.00173449	0.000786797
528	Diseases of the oral soft tissues, excluding lesions specific for gingiva and tongue	digestive	0.028000772	0.678902588	-0.00078531	0.000357402
446.4	Wegener's granulomatosis	circulatory system	0.028904802	0.690634228	-0.00048088	0.000220104
274.2	Crystal arthropathies	endocrine/metabolic	0.029415755	0.697078922	-0.00048988	0.000224934
550.5	Ventral hernia	digestive	0.02981261	0.70199399	-0.00250232	0.00115178
306	Other mental disorder	mental disorders	0.030285169	0.707745292	-0.00069146	0.000319186
377.1	Optic atrophy	sense organs	0.030423558	0.709408976	-0.00044145	0.000203947
750.21	Congenital anomalies of intestine	congenital anomalies	0.031583481	0.722995038	0.000514749	0.000239458
516.1	Hemoptysis	respiratory	0.031817057	0.725654971	0.00153271	0.000713985

343	Infantile cerebral palsy	neurological	0.033898462	0.748283936	0.000690909	0.000325704
614.52	Vaginitis and vulvovaginitis	genitourinary	0.034459582	0.754066133	-0.00059044	0.000279214
364.5	Corneal dystrophy	sense organs	0.034707234	0.756576744	-0.00057311	0.000271389
275	Disorders of mineral metabolism	endocrine/metabolic	0.036240151	0.771569984	-0.00030239	0.000144395
366.1	Nonsenile Cataract	sense organs	0.036251001	0.771672825	-0.00077778	0.000371413
701.2	Scar conditions and fibrosis of skin	dermatologic	0.036696108	0.775853167	0.00122573	0.00058672
709.3	Systemic sclerosis	dermatologic	0.03750939	0.783299431	0.00049151	0.000236282
446.3	Hypersensitivity angiitis	circulatory system	0.038075008	0.788335346	0.00030619	0.000147629
245.1	Thyroiditis, acute and subacute	endocrine/metabolic	0.039955816	0.804273853	-0.0005751	0.000279961
276.14	Hypopotassemia	endocrine/metabolic	0.04085545	0.811477785	-0.00222464	0.00108785
604	Disorders of penis	genitourinary	0.04155947	0.816934365	0.000587	0.000288041
355	Complex regional/central pain syndrome	neurological	0.041609261	0.817314389	0.000376662	0.000184873
300.9	Posttraumatic stress disorder	mental disorders	0.043073484	0.828152366	-0.00088335	0.000436654
302.1	Decreased libido	mental disorders	0.043192665	0.829006405	0.000499705	0.000247153
282.9	Other hereditary hemolytic anemias	hematopoietic	0.044227256	0.836248363	0.00035118	0.000174549
274.21	Chondrocalcinosis	endocrine/metabolic	0.045379531	0.843962244	-0.0008653	0.000432408
709.2	Sicca syndrome	dermatologic	0.045631001	0.845597995	0.000757125	0.000378793
550	Abdominal hernia	digestive	0.045733033	0.846256902	-0.00232994	0.00116622
741.3	Difficulty in walking	musculoskeletal	0.045900773	0.8473342	0.000473714	0.000237296
535.2	Atrophic gastritis	digestive	0.046538245	0.851361573	-0.00058992	0.000296371
379.4	Anomalies of pupillary function	sense organs	0.048398288	0.862529793	0.000304356	0.000154194

Supplementary Table 13. Protein interaction network including *PLPP4* as obtained from the STRING database. Visit STRING at https://string-db.org/ for more details on column names and method behind the table. *PPAPDC1A* is another name for *PLPP4* .

**node1**	**node2**	**node1_string_id**	**node2_string_id**	**neighborhood_on_chromosome**	**gene_fusion**	**phylogenetic_cooccurrence**	**homology**	**coexpression**	**experimentally_determined_interaction**	**database_annotated**	**automated_textmining**	**combined_score**
CDS1	MOGAT2	9606.ENSP00000295887	9606.ENSP00000198801	0	0	0	0	0	0	0	0.417	0.417
CDS1	PLD2	9606.ENSP0000029	9606.ENSP00000263	0	0	0	0	0.063	0	0.9	0.3	0.928
CDS1	MOGAT1	9606.ENSP0000029	9606.ENSP00000406	0	0	0	0	0	0	0	0.403	0.403
CDS1	DGAT1	9606.ENSP0000029	9606.ENSP00000482	0	0	0	0	0.065	0	0	0.403	0.418
CDS1	CDS2	9606.ENSP0000029	9606.ENSP00000419	0	0	0	0.975	0.062	0	0.8	0.481	0.806
CDS1	PPAPDC1A	9606.ENSP00000295887	9606.ENSP00000381302	0	0	0	0	0.062	0	0.9	0.187	0.917
CDS1	MBOAT1	9606.ENSP00000295887	9606.ENSP00000324944	0	0	0	0	0.087	0	0.9	0.25	0.925

CDS1	PLD1	9606.ENSP00000295887	9606.ENSP00000342793	0	0	0	0	0.063	0	0.9	0.387	0.937
CDS1	MBOAT2	9606.ENSP00000295887	9606.ENSP00000302177	0	0	0	0	0.087	0	0.9	0.447	0.945
CDS2	MOGAT2	9606.ENSP00000419879	9606.ENSP00000198801	0	0	0	0	0	0	0	0.446	0.446
CDS2	PLD2	9606.ENSP00000419879	9606.ENSP00000263088	0	0	0	0	0.063	0	0.9	0.215	0.92
CDS2	CDS1	9606.ENSP00000419879	9606.ENSP00000295887	0	0	0	0.975	0.062	0	0.8	0.481	0.806
CDS2	MBOAT2	9606.ENSP00000419879	9606.ENSP00000302177	0	0	0	0	0.087	0	0.9	0.386	0.939
CDS2	MBOAT1	9606.ENSP00000419879	9606.ENSP00000324944	0	0	0	0	0.087	0	0.9	0.25	0.925
CDS2	PLD1	9606.ENSP00000419879	9606.ENSP00000342793	0	0	0	0	0.063	0	0.9	0.32	0.93

CDS2	PPAPDC1A	9606.ENSP0000041 9879	9606.ENSP00000381 302	0	0	0	0	0.062	0	0.9	0.187	0.917
CDS2	MOGAT1	9606.ENSP0000041 9879	9606.ENSP00000406 674	0	0	0	0	0	0	0	0.468	0.468
DGAT1	MOGAT2	9606.ENSP0000048 2264	9606.ENSP00000198 801	0	0	0	0	0.062	0.347	0.9	0.892	0.992
DGAT1	DGAT2	9606.ENSP0000048 2264	9606.ENSP00000228 027	0	0	0	0	0.064	0.347	0.8	0.948	0.992
DGAT1	CDS1	9606.ENSP0000048 2264	9606.ENSP00000295 887	0	0	0	0	0.065	0	0	0.403	0.418
DGAT1	MBOAT2	9606.ENSP0000048 2264	9606.ENSP00000302 177	0	0	0	0	0	0	0	0.839	0.839
DGAT1	MBOAT1	9606.ENSP0000048 2264	9606.ENSP00000324 944	0	0	0	0	0	0	0	0.672	0.672
DGAT1	PPAPDC1A	9606.ENSP0000048 2264	9606.ENSP00000381 302	0	0	0	0	0.062	0	0.9	0.325	0.931

DGAT1	MOGAT1	9606.ENSP0000482264	9606.ENSP00000406674	0	0	0	0	0.062	0.347	0.9	0.946	0.996
DGAT2	MOGAT2	9606.ENSP0000228027	9606.ENSP00000198801	0	0	0	0.937	0.049	0.152	0.9	0.91	0.917
DGAT2	MBOAT2	9606.ENSP0000228027	9606.ENSP00000302177	0	0	0	0	0.054	0	0	0.611	0.616
DGAT2	MOGAT1	9606.ENSP0000228027	9606.ENSP00000406674	0	0	0	0.942	0.062	0.145	0.9	0.913	0.917
DGAT2	PPAPDC1A	9606.ENSP0000228027	9606.ENSP00000381302	0	0	0	0	0.062	0	0.9	0.274	0.926
DGAT2	DGAT1	9606.ENSP0000228027	9606.ENSP00000482264	0	0	0	0	0.064	0.347	0.8	0.948	0.992
MBOAT1	PLD2	9606.ENSP0000324944	9606.ENSP00000263088	0	0	0	0	0.052	0	0.65	0.284	0.741
MBOAT1	CDS1	9606.ENSP0000324944	9606.ENSP00000295887	0	0	0	0	0.087	0	0.9	0.25	0.925

MBOAT1	MBOAT2	9606.ENSP0000032 4944	9606.ENSP00000302 177	0	0	0	0.955	0	0	0.8	0.653	0.805
MBOAT1	DGAT1	9606.ENSP0000032 4944	9606.ENSP00000482 264	0	0	0	0	0	0	0	0.672	0.672
MBOAT1	PLD1	9606.ENSP0000032 4944	9606.ENSP00000342 793	0	0	0	0	0.052	0	0.65	0.312	0.751
MBOAT1	PPAPDC1A	9606.ENSP0000032 4944	9606.ENSP00000381 302	0	0	0	0	0.049	0.069	0.9	0.158	0.915
MBOAT1	CDS2	9606.ENSP0000032 4944	9606.ENSP00000419 879	0	0	0	0	0.087	0	0.9	0.25	0.925
MBOAT2	MOGAT2	9606.ENSP0000030 2177	9606.ENSP00000198 801	0	0	0	0	0.054	0	0	0.404	0.412
MBOAT2	DGAT2	9606.ENSP0000030 2177	9606.ENSP00000228 027	0	0	0	0	0.054	0	0	0.611	0.616
MBOAT2	PLD2	9606.ENSP0000030 2177	9606.ENSP00000263 088	0	0	0	0	0.052	0	0.65	0.434	0.795

MBOAT2	CDS1	9606.ENSP0000030 2177	9606.ENSP00000295 887	0	0	0	0	0.087	0	0.9	0.447	0.945
MBOAT2	PLD1	9606.ENSP0000030 2177	9606.ENSP00000342 793	0	0	0	0	0.052	0	0.65	0.329	0.757
MBOAT2	MBOAT1	9606.ENSP0000030 2177	9606.ENSP00000324 944	0	0	0	0.955	0	0	0.8	0.653	0.805
MBOAT2	DGAT1	9606.ENSP0000030 2177	9606.ENSP00000482 264	0	0	0	0	0	0	0	0.839	0.839
MBOAT2	PPAPDC1A	9606.ENSP0000030 2177	9606.ENSP00000381 302	0	0	0	0	0.049	0.069	0.9	0.129	0.912
MBOAT2	CDS2	9606.ENSP0000030 2177	9606.ENSP00000419 879	0	0	0	0	0.087	0	0.9	0.386	0.939
MOGAT1	MOGAT2	9606.ENSP0000040 6674	9606.ENSP00000198 801	0	0	0	0.953	0	0	0.8	0.908	0.808
MOGAT1	DGAT2	9606.ENSP0000040 6674	9606.ENSP00000228 027	0	0	0	0.942	0.062	0.145	0.9	0.913	0.917

MOGAT1	CDS1	9606.ENSP0000040 6674	9606.ENSP00000295 887	0	0	0	0	0	0	0	0.403	0.403
MOGAT1	PPAPDC1A	9606.ENSP0000040 6674	9606.ENSP00000381 302	0	0	0	0	0.062	0	0.9	0.249	0.923
MOGAT1	CDS2	9606.ENSP0000040 6674	9606.ENSP00000419 879	0	0	0	0	0	0	0	0.468	0.468
MOGAT1	DGAT1	9606.ENSP0000040 6674	9606.ENSP00000482 264	0	0	0	0	0.062	0.347	0.9	0.946	0.996
MOGAT2	MBOAT2	9606.ENSP0000019 8801	9606.ENSP00000302 177	0	0	0	0	0.054	0	0	0.404	0.412
MOGAT2	CDS1	9606.ENSP0000019 8801	9606.ENSP00000295 887	0	0	0	0	0	0	0	0.417	0.417
MOGAT2	CDS2	9606.ENSP0000019 8801	9606.ENSP00000419 879	0	0	0	0	0	0	0	0.446	0.446
MOGAT2	MOGAT1	9606.ENSP0000019 8801	9606.ENSP00000406 674	0	0	0	0.953	0	0	0.8	0.908	0.808

MOGAT2	DGAT2	9606.ENSP0000198801	9606.ENSP00000228027	0	0	0	0.937	0.049	0.152	0.9	0.91	0.917
MOGAT2	PPAPDC1A	9606.ENSP0000198801	9606.ENSP00000381302	0	0	0	0	0.062	0	0.9	0.249	0.923
MOGAT2	DGAT1	9606.ENSP0000198801	9606.ENSP00000482264	0	0	0	0	0.062	0.347	0.9	0.892	0.992
PLD1	PLD2	9606.ENSP0000342793	9606.ENSP00000263088	0	0	0	0.955	0	0	0.8	0.836	0.807
PLD1	CDS1	9606.ENSP0000342793	9606.ENSP00000295887	0	0	0	0	0.063	0	0.9	0.387	0.937
PLD1	MBOAT2	9606.ENSP0000342793	9606.ENSP00000302177	0	0	0	0	0.052	0	0.65	0.329	0.757
PLD1	MBOAT1	9606.ENSP0000342793	9606.ENSP00000324944	0	0	0	0	0.052	0	0.65	0.312	0.751
PLD1	PPAPDC1A	9606.ENSP0000342793	9606.ENSP00000381302	0.049	0	0	0	0	0.08	0.9	0.14	0.914

PLD1	CDS2	9606.ENSP0000342793	9606.ENSP00000419879	0	0	0	0	0.063	0	0.9	0.32	0.93
PLD2	MBOAT1	9606.ENSP0000263088	9606.ENSP00000324944	0	0	0	0	0.052	0	0.65	0.284	0.741
PLD2	MBOAT2	9606.ENSP0000263088	9606.ENSP00000302177	0	0	0	0	0.052	0	0.65	0.434	0.795
PLD2	PLD1	9606.ENSP0000263088	9606.ENSP00000342793	0	0	0	0.955	0	0	0.8	0.836	0.807
PLD2	PPAPDC1A	9606.ENSP0000263088	9606.ENSP00000381302	0.049	0	0	0	0	0.08	0.9	0.14	0.914
PLD2	CDS2	9606.ENSP0000263088	9606.ENSP00000419879	0	0	0	0	0.063	0	0.9	0.215	0.92
PLD2	CDS1	9606.ENSP0000263088	9606.ENSP00000295887	0	0	0	0	0.063	0	0.9	0.3	0.928
PPAPDC1A	MOGAT2	9606.ENSP0000381302	9606.ENSP00000198801	0	0	0	0	0.062	0	0.9	0.249	0.923

PPAPDC1A	DGAT2	9606.ENSP0000381302	9606.ENSP00000228027	0	0	0	0	0.062	0	0.9	0.274	0.926
PPAPDC1A	PLD2	9606.ENSP0000381302	9606.ENSP00000263088	0.049	0	0	0	0	0.08	0.9	0.14	0.914
PPAPDC1A	CDS1	9606.ENSP0000381302	9606.ENSP00000295887	0	0	0	0	0.062	0	0.9	0.187	0.917
PPAPDC1A	MBOAT2	9606.ENSP0000381302	9606.ENSP00000302177	0	0	0	0	0.049	0.069	0.9	0.129	0.912
PPAPDC1A	MBOAT1	9606.ENSP0000381302	9606.ENSP00000324944	0	0	0	0	0.049	0.069	0.9	0.158	0.915
PPAPDC1A	PLD1	9606.ENSP0000381302	9606.ENSP00000342793	0.049	0	0	0	0	0.08	0.9	0.14	0.914
PPAPDC1A	CDS2	9606.ENSP0000381302	9606.ENSP00000419879	0	0	0	0	0.062	0	0.9	0.187	0.917
PPAPDC1A	MOGAT1	9606.ENSP0000381302	9606.ENSP00000406674	0	0	0	0	0.062	0	0.9	0.249	0.923

PPAPDC1A	DGAT1	9606.ENSP0000381302	9606.ENSP00000482264	0	0	0	0	0.062	0	0.9	0.325	0.931

Supplementary Table 14. Protein annotations of STRING network nodes in Supplementary Table 14.

node	identifier	annotation	domain_summary_url
CDS1	9606.ENSP00000295887	Phosphatidate cytidylyltransferase 1; Provides CDP-diacylglycerol, an important precursor for the synthesis of phosphatidylinositol (PtdIns), phosphatidylglycerol, and cardiolipin. Overexpression may amplify cellular signaling responses from cytokines. May also play an important role in the signal transduction mechanism of retina and neural cells	https://smart.embl.de/smart/DDvec.cgi?smart=461:Pfam_CTP_transf_1(87|417)+
CDS2	9606.ENSP00000419879	Phosphatidate cytidylyltransferase 2; Provides CDP-diacylglycerol, an important precursor for the synthesis of phosphatidylinositol, phosphatidylglycerol, and cardiolipin	https://smart.embl.de/smart/DDvec.cgi?smart=445:Pfam_CTP_transf_1(70|400)+
DGAT1	9606.ENSP00000482264	Diacylglycerol O-acyltransferase 1; Catalyzes the terminal and only committed step in triacylglycerol synthesis by using diacylglycerol and fatty acyl CoA as substrates. In contrast to DGAT2 it is not essential for survival. May be involved in VLDL (very low density lipoprotein) assembly. In liver, plays a role in esterifying exogenous fatty acids to glycerol. Functions as the major acyl-CoA retinol acyltransferase (ARAT) in the skin, where it acts to maintain retinoid homeostasis and prevent retinoid toxicity leading to skin and hair disorders; Belongs to the membrane-bound acyltransf [...]	https://smart.embl.de/smart/DDvec.cgi?smart=488:TRANS(87|109)+TRANS(129|151)+Pfam_MBOAT(153|474)+
DGAT2	9606.ENSP00000228027	Diacylglycerol O-acyltransferase 2; Essential acyltransferase that catalyzes the terminal and only committed step in triacylglycerol synthesis by using diacylglycerol and fatty acyl CoA as substrates. Required for synthesis and storage of intracellular triglycerides. Probably plays a central role in cytosolic lipid accumulation. In liver, is primarily responsible for incorporating endogenously synthesized fatty acids into triglycerides (By similarity). Functions also as an acyl-CoA retinol acyltransferase (ARAT); Diacylglycerol O-acyltransferase 2 family	https://smart.embl.de/smart/DDvec.cgi?smart=388:Pfam_DAGAT(92|388)+

MBOAT1	9606.ENSP00000324944	Lysophospholipid acyltransferase 1; Acyltransferase which mediates the conversion of lysophosphatidylserine (1-acyl-2-hydroxy-sn-glycero-3-phospho-L- serine or LPS) into phosphatidylserine (1,2-diacyl-sn-glycero-3- phospho-L-serine or PS) (LPSAT activity). Prefers oleoyl-CoA as the acyl donor. Lysophospholipid acyltransferases (LPLATs) catalyze the reacylation step of the phospholipid remodeling pathway also known as the Lands cycle; Membrane bound O-acyltransferases	https://smart.embl.de/smart/DDvec.cgi?smart=495:Pfam_MBOAT(33|439)+TRANS(456|473)+
MBOAT2	9606.ENSP00000302177	Lysophospholipid acyltransferase 2; Acyltransferase which mediates the conversion of lysophosphatidylethanolamine (1-acyl-sn-glycero-3-phosphoethanolamine or LPE) into phosphatidylethanolamine (1,2- diacyl-sn-glycero-3-phosphoethanolamine or PE) (LPEAT activity). Catalyzes also the acylation of lysophosphatidic acid (LPA) into phosphatidic acid (PA) (LPAAT activity). Has also a very weak lysophosphatidylcholine acyltransferase (LPCAT activity). Prefers oleoyl-CoA as the acyl donor. Lysophospholipid acyltransferases (LPLATs) catalyze the reacylation step of the phospholipid remodeling [...]	https://smart.embl.de/smart/DDvec.cgi?smart=520:Pfam_MBOAT(23|435)+TRANS(444|463)+
MOGAT1	9606.ENSP00000406674	2-acylglycerol O-acyltransferase 1; Catalyzes the formation of diacylglycerol from 2- monoacylglycerol and fatty acyl-CoA. Probably not involved in absorption of dietary fat in the small intestine (By similarity); Belongs to the diacylglycerol acyltransferase family	https://smart.embl.de/smart/DDvec.cgi?smart=335:Pfam_DAGAT(40|335)+
MOGAT2	9606.ENSP00000198801	2-acylglycerol O-acyltransferase 2; Catalyzes the formation of diacylglycerol from 2- monoacylglycerol and fatty acyl-CoA. Has a preference toward monoacylglycerols containing unsaturated fatty acids in an order of C18:3 > C18:2 > C18:1 > C18:0. Plays a central role in absorption of dietary fat in the small intestine by catalyzing the resynthesis of triacylglycerol in enterocytes. May play a role in diet-induced obesity	https://smart.embl.de/smart/DDvec.cgi?smart=334:Pfam_DAGAT(39|334)+
PLD1	9606.ENSP00000342793	Phospholipase D1; Implicated as a critical step in numerous cellular pathways, including signal transduction, membrane trafficking, and the regulation of mitosis. May be involved in the regulation of perinuclear intravesicular membrane traffic (By similarity); Phospholipases	https://smart.embl.de/smart/DDvec.cgi?smart=1074:PX(79|209)+PH(220|330)+PLDc(459|486)+PLDc(891|918)+

PLD2	9606.ENSP00000263088	Phospholipase D2; May have a role in signal-induced cytoskeletal regulation and/or endocytosis; Phospholipases	https://smart.embl.de/smart/DDvec.cgi?smart=933:PX(64|192)+PH(203|313)+PLDc(437|464)+PLDc(751|778)+
PPAPDC1A	9606.ENSP00000381302	Phospholipid phosphatase 4; Displays magnesium-independent phosphatidate phosphatase activity in vitro. Catalyzes the conversion of phosphatidic acid to diacylglycerol; Belongs to the PA-phosphatase related phosphoesterase family	https://smart.embl.de/smart/DDvec.cgi?smart=271:TRANS(7|24)+TRANS(49|71)+acidPPc(83|222)+

Supplementary Table 15. Protein interaction network including *ACTR3B* as obtained from the STRING database.

node1	node2	node1_string_id	node2_string_id	neighborhood_on_chromosome	gene_fusion	phylogenetic_cooccurrence	homology	coexpression	experimentally_determined_interaction	database_annotated	automated_textmining	combined_score
ACTB	WASL	9606.ENSP00000494750	9606.ENSP00000223023	0	0	0	0	0.046	0.492	0.9	0.63	0.979
ACTB	ARPC3	9606.ENSP00000494750	9606.ENSP00000228825	0	0	0	0	0.336	0.405	0.9	0.297	0.968
ACTB	ACTR3B	9606.ENSP00000494750	9606.ENSP00000256001	0	0	0	0.871	0.067	0.298	0.9	0.674	0.975
ACTB	ARPC1A	9606.ENSP00000494750	9606.ENSP00000262942	0	0	0	0	0.121	0.362	0.9	0.174	0.947
ACTB	ARPC5	9606.ENSP00000494750	9606.ENSP00000294742	0	0	0	0	0.202	0.281	0.9	0.272	0.952
ACTB	ARPC2	9606.ENSP00000494750	9606.ENSP00000295685	0	0	0	0	0.259	0.365	0.9	0.305	0.962
ACTB	ARPC5L	9606.ENSP00000494750	9606.ENSP00000345361	0	0	0	0	0.049	0.388	0.9	0.171	0.945

ACTB	WAS	9606.ENSP0000494750	9606.ENSP00000365891	0	0	0	0	0.087	0.284	0.9	0.387	0.954
ACTB	ACTR2	9606.ENSP0000494750	9606.ENSP00000367220	0	0	0	0.935	0.335	0.525	0.9	0.128	0.968
ACTB	ARPC4	9606.ENSP0000494750	9606.ENSP00000388169	0	0	0	0	0.335	0.414	0.9	0.12	0.961
ACTR2	WASL	9606.ENSP0000367220	9606.ENSP00000223023	0	0	0	0	0.056	0.985	0.9	0.993	0.999
ACTR2	ARPC3	9606.ENSP0000367220	9606.ENSP00000228825	0	0	0	0	0.769	0.997	0.9	0.998	0.999
ACTR2	ACTR3B	9606.ENSP0000367220	9606.ENSP00000256001	0	0	0.079	0.814	0.39	0.962	0.65	0.975	0.999
ACTR2	ARPC1A	9606.ENSP0000367220	9606.ENSP00000262942	0	0	0	0	0.112	0.995	0.72	0.949	0.999
ACTR2	ARPC5	9606.ENSP0000367220	9606.ENSP00000294742	0	0	0	0	0.782	0.997	0.9	0.997	0.999

ACTR2	ARPC2	9606.ENSP00000367220	9606.ENSP00000295685	0	0	0	0	0.759	0.995	0.9	0.997	0.999
ACTR2	ARPC5L	9606.ENSP00000367220	9606.ENSP00000345361	0	0	0	0	0.118	0.997	0.54	0.647	0.999
ACTR2	WAS	9606.ENSP00000367220	9606.ENSP00000365891	0	0	0	0	0.056	0.861	0.9	0.997	0.999
ACTR2	ACTB	9606.ENSP00000367220	9606.ENSP00000494750	0	0	0	0.935	0.335	0.525	0.9	0.128	0.968
ACTR2	ARPC4	9606.ENSP00000367220	9606.ENSP00000388169	0	0	0	0	0.779	0.997	0.9	0.809	0.999
ACTR3B	WASL	9606.ENSP00000256001	9606.ENSP00000223023	0	0	0	0	0.05	0.52	0.9	0.446	0.971
ACTR3B	ARPC3	9606.ENSP00000256001	9606.ENSP00000228825	0	0	0	0	0.053	0.97	0	0.69	0.99
ACTR3B	ARPC1A	9606.ENSP00000256001	9606.ENSP00000262942	0	0	0	0	0.269	0.915	0	0.462	0.964

ACTR3B	ACTB	9606.ENSP00000256001	9606.ENSP00000494750	0	0	0	0.871	0.067	0.298	0.9	0.674	0.975
ACTR3B	ARPC4	9606.ENSP00000256001	9606.ENSP00000388169	0	0	0	0	0.072	0.962	0	0.412	0.977
ACTR3B	WAS	9606.ENSP00000256001	9606.ENSP00000365891	0	0	0	0	0	0.52	0.9	0.588	0.978
ACTR3B	ARPC5L	9606.ENSP00000256001	9606.ENSP00000345361	0	0	0	0	0.119	0.92	0	0.731	0.979
ACTR3B	ARPC5	9606.ENSP00000256001	9606.ENSP00000294742	0	0	0	0	0.119	0.919	0	0.736	0.979
ACTR3B	ARPC2	9606.ENSP00000256001	9606.ENSP00000295685	0	0	0	0	0.188	0.927	0	0.727	0.982
ACTR3B	ACTR2	9606.ENSP00000256001	9606.ENSP00000367220	0	0	0.079	0.814	0.39	0.962	0.65	0.975	0.999
ARPC1A	WASL	9606.ENSP00000262942	9606.ENSP00000223023	0	0	0	0	0.063	0.405	0.9	0.697	0.98

ARPC1A	ARPC3	9606.ENSP0000026 2942	9606.ENSP000002288 25	0	0	0	0	0.147	0.995	0.9	0.955	0.999
ARPC1A	ACTR3B	9606.ENSP0000026 2942	9606.ENSP000002560 01	0	0	0	0	0.269	0.915	0	0.462	0.964
ARPC1A	ACTB	9606.ENSP0000026 2942	9606.ENSP000004947 50	0	0	0	0	0.121	0.362	0.9	0.174	0.947
ARPC1A	WAS	9606.ENSP0000026 2942	9606.ENSP000003658 91	0	0	0	0	0.042	0.405	0.9	0.48	0.966
ARPC1A	ACTR2	9606.ENSP0000026 2942	9606.ENSP000003672 20	0	0	0	0	0.112	0.995	0.72	0.949	0.999
ARPC1A	ARPC5	9606.ENSP0000026 2942	9606.ENSP000002947 42	0	0	0	0	0.062	0.992	0.9	0.893	0.999
ARPC1A	ARPC4	9606.ENSP0000026 2942	9606.ENSP000003881 69	0	0	0	0	0.118	0.994	0.9	0.336	0.999
ARPC1A	ARPC5L	9606.ENSP0000026 2942	9606.ENSP000003453 61	0	0	0	0	0.056	0.953	0.9	0.865	0.999

ARPC1A	ARPC2	9606.ENSP00000262942	9606.ENSP00000295685	0	0	0	0	0.106	0.992	0.9	0.935	0.999
ARPC2	WASL	9606.ENSP00000295685	9606.ENSP00000223023	0	0	0	0	0	0.91	0.9	0.848	0.998
ARPC2	ARPC3	9606.ENSP00000295685	9606.ENSP00000228825	0	0	0	0	0.829	0.997	0.9	0.999	0.999
ARPC2	ACTR3B	9606.ENSP00000295685	9606.ENSP00000256001	0	0	0	0	0.188	0.927	0	0.727	0.982
ARPC2	ARPC1A	9606.ENSP00000295685	9606.ENSP00000262942	0	0	0	0	0.106	0.992	0.9	0.935	0.999
ARPC2	ARPC5	9606.ENSP00000295685	9606.ENSP00000294742	0	0	0	0	0.792	0.992	0.9	0.999	0.999
ARPC2	ACTB	9606.ENSP00000295685	9606.ENSP00000494750	0	0	0	0	0.259	0.365	0.9	0.305	0.962
ARPC2	WAS	9606.ENSP00000295685	9606.ENSP00000365891	0	0	0	0	0.06	0.573	0.9	0.944	0.997

ARPC2	ACTR2	9606.ENSP00000295685	9606.ENSP00000367220	0	0	0	0	0.759	0.995	0.9	0.997	0.999
ARPC2	ARPC5L	9606.ENSP00000295685	9606.ENSP00000345361	0	0	0	0	0.15	0.992	0.9	0.799	0.999
ARPC2	ARPC4	9606.ENSP00000295685	9606.ENSP00000388169	0	0	0	0	0.784	0.997	0.9	0.884	0.999
ARPC3	WASL	9606.ENSP00000228825	9606.ENSP00000223023	0	0	0	0	0	0.93	0.9	0.767	0.998
ARPC3	ACTB	9606.ENSP00000228825	9606.ENSP00000494750	0	0	0	0	0.336	0.405	0.9	0.297	0.968
ARPC3	ACTR3B	9606.ENSP00000228825	9606.ENSP00000256001	0	0	0	0	0.053	0.97	0	0.69	0.99
ARPC3	WAS	9606.ENSP00000228825	9606.ENSP00000365891	0	0	0	0	0.062	0.796	0.9	0.914	0.998
ARPC3	ARPC2	9606.ENSP00000228825	9606.ENSP00000295685	0	0	0	0	0.829	0.997	0.9	0.999	0.999

ARPC3	ARPC5L	9606.ENSP000002288 25	9606.ENSP00000345361	0	0	0	0	0.211	0.997	0.9	0.816	0.999
ARPC3	ARPC4	9606.ENSP000002288 25	9606.ENSP00000388169	0	0	0	0	0.912	0.997	0.9	0.81	0.999
ARPC3	ARPC5	9606.ENSP000002288 25	9606.ENSP00000294742	0	0	0	0	0.803	0.997	0.9	0.999	0.999
ARPC3	ACTR2	9606.ENSP000002288 25	9606.ENSP00000367220	0	0	0	0	0.769	0.997	0.9	0.998	0.999
ARPC3	ARPC1A	9606.ENSP000002288 25	9606.ENSP00000262942	0	0	0	0	0.147	0.995	0.9	0.955	0.999
ARPC4	WASL	9606.ENSP000003881 69	9606.ENSP00000223023	0	0	0	0	0	0.934	0.9	0.262	0.994
ARPC4	ARPC3	9606.ENSP000003881 69	9606.ENSP00000228825	0	0	0	0	0.912	0.997	0.9	0.81	0.999
ARPC4	ACTR3B	9606.ENSP000003881 69	9606.ENSP00000256001	0	0	0	0	0.072	0.962	0	0.412	0.977

ARPC4	ARPC1A	9606.ENSP00000388169	9606.ENSP00000262942	0	0	0	0	0.118	0.994	0.9	0.336	0.999
ARPC4	ARPC5	9606.ENSP00000388169	9606.ENSP00000294742	0	0	0	0	0.786	0.997	0.9	0.845	0.999
ARPC4	ARPC2	9606.ENSP00000388169	9606.ENSP00000295685	0	0	0	0	0.784	0.997	0.9	0.884	0.999
ARPC4	ARPC5L	9606.ENSP00000388169	9606.ENSP00000345361	0	0	0	0	0.255	0.997	0.9	0.349	0.999
ARPC4	WAS	9606.ENSP00000388169	9606.ENSP00000365891	0	0	0	0	0.061	0.566	0.9	0.591	0.981
ARPC4	ACTR2	9606.ENSP00000388169	9606.ENSP00000367220	0	0	0	0	0.779	0.997	0.9	0.809	0.999
ARPC4	ACTB	9606.ENSP00000388169	9606.ENSP00000494750	0	0	0	0	0.335	0.414	0.9	0.12	0.961
ARPC5	WASL	9606.ENSP00000294742	9606.ENSP00000223023	0	0	0	0	0	0.853	0.9	0.577	0.993

ARPC5	ARPC3	9606.ENSP000000294742	9606.ENSP000000228825	0	0	0	0	0.803	0.997	0.9	0.999	0.999
ARPC5	ACTR3B	9606.ENSP000000294742	9606.ENSP000000256001	0	0	0	0	0.119	0.919	0	0.736	0.979
ARPC5	ARPC1A	9606.ENSP000000294742	9606.ENSP000000262942	0	0	0	0	0.062	0.992	0.9	0.893	0.999
ARPC5	ARPC5L	9606.ENSP000000294742	9606.ENSP000000345361	0	0	0.057	0.967	0.089	0.447	0.9	0.062	0.947
ARPC5	ACTB	9606.ENSP000000294742	9606.ENSP000000494750	0	0	0	0	0.202	0.281	0.9	0.272	0.952
ARPC5	WAS	9606.ENSP000000294742	9606.ENSP000000365891	0	0	0	0	0.068	0.299	0.9	0.907	0.993
ARPC5	ARPC2	9606.ENSP000000294742	9606.ENSP000000295685	0	0	0	0	0.792	0.992	0.9	0.999	0.999
ARPC5	ACTR2	9606.ENSP000000294742	9606.ENSP000000367220	0	0	0	0	0.782	0.997	0.9	0.997	0.999

ARPC5	ARPC4	9606.ENSP0000029 4742	9606.ENSP000003881 69	0	0	0	0	0.786	0.997	0.9	0.845	0.999
ARPC5L	WASL	9606.ENSP0000034 5361	9606.ENSP000002230 23	0	0	0	0	0.047	0.299	0.9	0.481	0.96
ARPC5L	ARPC3	9606.ENSP0000034 5361	9606.ENSP000002288 25	0	0	0	0	0.211	0.997	0.9	0.816	0.999
ARPC5L	ACTR3B	9606.ENSP0000034 5361	9606.ENSP000002560 01	0	0	0	0	0.119	0.92	0	0.731	0.979
ARPC5L	ARPC1A	9606.ENSP0000034 5361	9606.ENSP000002629 42	0	0	0	0	0.056	0.953	0.9	0.865	0.999
ARPC5L	ARPC5	9606.ENSP0000034 5361	9606.ENSP000002947 42	0	0	0.057	0.967	0.089	0.447	0.9	0.062	0.947
ARPC5L	ARPC2	9606.ENSP0000034 5361	9606.ENSP000002956 85	0	0	0	0	0.15	0.992	0.9	0.799	0.999
ARPC5L	ACTB	9606.ENSP0000034 5361	9606.ENSP000004947 50	0	0	0	0	0.049	0.388	0.9	0.171	0.945

ARPC5L	WAS	9606.ENSP0000345361	9606.ENSP00000365891	0	0	0	0	0.055	0.299	0.9	0.403	0.955
ARPC5L	ACTR2	9606.ENSP0000345361	9606.ENSP00000367220	0	0	0	0	0.118	0.997	0.54	0.647	0.999
ARPC5L	ARPC4	9606.ENSP0000345361	9606.ENSP00000388169	0	0	0	0	0.255	0.997	0.9	0.349	0.999
WAS	WASL	9606.ENSP0000365891	9606.ENSP00000223023	0	0	0.075	0.922	0	0	0.4	0.866	0.919
WAS	ARPC3	9606.ENSP0000365891	9606.ENSP00000228825	0	0	0	0	0.062	0.796	0.9	0.914	0.998
WAS	ACTR3B	9606.ENSP0000365891	9606.ENSP00000256001	0	0	0	0	0	0.52	0.9	0.588	0.978
WAS	ARPC1A	9606.ENSP0000365891	9606.ENSP00000262942	0	0	0	0	0.042	0.405	0.9	0.48	0.966
WAS	ARPC5	9606.ENSP0000365891	9606.ENSP00000294742	0	0	0	0	0.068	0.299	0.9	0.907	0.993

WAS	ARPC2	9606.ENSP0000036 5891	9606.ENSP000002956 85	0	0	0	0	0.06	0.573	0.9	0.944	0.997
WAS	ARPC5L	9606.ENSP0000036 5891	9606.ENSP000003453 61	0	0	0	0	0.055	0.299	0.9	0.403	0.955
WAS	ACTB	9606.ENSP0000036 5891	9606.ENSP000004947 50	0	0	0	0	0.087	0.284	0.9	0.387	0.954
WAS	ARPC4	9606.ENSP0000036 5891	9606.ENSP000003881 69	0	0	0	0	0.061	0.566	0.9	0.591	0.981
WAS	ACTR2	9606.ENSP0000036 5891	9606.ENSP000003672 20	0	0	0	0	0.056	0.861	0.9	0.997	0.999
WASL	WAS	9606.ENSP0000022 3023	9606.ENSP000003658 91	0	0	0.075	0.922	0	0	0.4	0.866	0.919
WASL	ARPC5L	9606.ENSP0000022 3023	9606.ENSP000003453 61	0	0	0	0	0.047	0.299	0.9	0.481	0.96
WASL	ACTR3B	9606.ENSP0000022 3023	9606.ENSP000002560 01	0	0	0	0	0.05	0.52	0.9	0.446	0.971

WASL	ACTB	9606.ENSP000002 23023	9606.ENSP000004 94750	0	0	0	0	0.046	0.492	0.9	0.63	0.979
WASL	ARPC1A	9606.ENSP000002 23023	9606.ENSP000002 62942	0	0	0	0	0.063	0.405	0.9	0.697	0.98
WASL	ARPC5	9606.ENSP000002 23023	9606.ENSP000002 94742	0	0	0	0	0	0.853	0.9	0.577	0.993
WASL	ARPC4	9606.ENSP000002 23023	9606.ENSP000003 88169	0	0	0	0	0	0.934	0.9	0.262	0.994
WASL	ARPC2	9606.ENSP000002 23023	9606.ENSP000002 95685	0	0	0	0	0	0.91	0.9	0.848	0.998
WASL	ARPC3	9606.ENSP000002 23023	9606.ENSP000002 28825	0	0	0	0	0	0.93	0.9	0.767	0.998
WASL	ACTR2	9606.ENSP000002 23023	9606.ENSP000003 67220	0	0	0	0	0.056	0.985	0.9	0.993	0.999

Supplementary Table 16. Protein annotations of STRING network nodes in Supplementary Table 17.

node	identifier	annotation	domain_summary_url
ACTB	9606.ENSP00000494750	Actin, cytoplasmic 1, N-terminally processed; Actin is a highly conserved protein that polymerizes to produce filaments that form cross-linked networks in the cytoplasm of cells. Actin exists in both monomeric (G-actin) and polymeric (F-actin) forms, both forms playing key functions, such as cell motility and contraction. In addition to their role in the cytoplasmic cytoskeleton, G- and F-actin also localize in the nucleus, and regulate gene transcription and motility and repair of damaged DNA.	https://smart.embl.de/smart/DDvec.cgi?smart=375:ACTIN(5|375)+
ACTR2	9606.ENSP00000367220	Actin-related protein 2; ATP-binding component of the Arp2/3 complex, a multiprotein complex that mediates actin polymerization upon stimulation by nucleation-promoting factor (NPF). The Arp2/3 complex mediates the formation of branched actin networks in the cytoplasm, providing the force for cell motility. Seems to contact the pointed end of the daughter actin filament. In addition to its role in the cytoplasmic cytoskeleton, the Arp2/3 complex also promotes actin polymerization in the nucleus, thereby regulating gene transcription and repair of damaged DNA. The Arp2/3 complex promote [...]	https://smart.embl.de/smart/DDvec.cgi?smart=399:ACTIN(6|395)+
ACTR3B	9606.ENSP00000256001	Actin-related protein 3B; Plays a role in the organization of the actin cytoskeleton. May function as ATP-binding component of the Arp2/3 complex which is involved in regulation of actin polymerization and together with an activating nucleation-promoting factor (NPF) mediates the formation of branched actin networks. May decrease the metastatic potential of tumors.	https://smart.embl.de/smart/DDvec.cgi?smart=418:ACTIN(5|413)+
ARPC1A	9606.ENSP00000262942	Actin-related protein 2/3 complex subunit 1A; Probably functions as component of the Arp2/3 complex which is involved in regulation of actin polymerization and together with an activating nucleation-promoting factor (NPF) mediates the formation of branched actin networks; Belongs to the WD repeat ARPC1 family.	https://smart.embl.de/smart/DDvec.cgi?smart=370:WD40(41|80)+WD40(85|124)+WD40(134|170)+WD40(191|232)+WD40(235|273)+WD40(312|356)+

ARPC2	9606.ENSP00000295685	Actin-related protein 2/3 complex subunit 2; Actin-binding component of the Arp2/3 complex, a multiprotein complex that mediates actin polymerization upon stimulation by nucleation-promoting factor (NPF). The Arp2/3 complex mediates the formation of branched actin networks in the cytoplasm, providing the force for cell motility. Seems to contact the mother actin filament. In addition to its role in the cytoplasmic cytoskeleton, the Arp2/3 complex also promotes actin polymerization in the nucleus, thereby regulating gene transcription and repair of damaged DNA. The Arp2/3 complex promot [...]	https://smart.embl.de/smart/DDvec.cgi?smart=300:Pfam_P34-Arc(57|284)+
ARPC3	9606.ENSP00000228825	Actin-related protein 2/3 complex subunit 3; Component of the Arp2/3 complex, a multiprotein complex that mediates actin polymerization upon stimulation by nucleation-promoting factor (NPF). The Arp2/3 complex mediates the formation of branched actin networks in the cytoplasm, providing the force for cell motility. In addition to its role in the cytoplasmic cytoskeleton, the Arp2/3 complex also promotes actin polymerization in the nucleus, thereby regulating gene transcription and repair of damaged DNA. The Arp2/3 complex promotes homologous recombination (HR) repair in response to DNA [...]	https://smart.embl.de/smart/DDvec.cgi?smart=178:Pfam_P21-Arc(1|173)+
ARPC4	9606.ENSP00000388169	Actin-related protein 2/3 complex subunit 4; Actin-binding component of the Arp2/3 complex, a multiprotein complex that mediates actin polymerization upon stimulation by nucleation-promoting factor (NPF). The Arp2/3 complex mediates the formation of branched actin networks in the cytoplasm, providing the force for cell motility. In addition to its role in the cytoplasmic cytoskeleton, the Arp2/3 complex also promotes actin polymerization in the nucleus, thereby regulating gene transcription and repair of damaged DNA. The Arp2/3 complex promotes homologous recombination (HR) repair in r [...]	https://smart.embl.de/smart/DDvec.cgi?smart=187:Pfam_ARPC4(20|186)+
ARPC5	9606.ENSP00000294742	Actin-related protein 2/3 complex subunit 5; Component of the Arp2/3 complex, a multiprotein complex that mediates actin polymerization upon stimulation by nucleation-promoting factor (NPF). The Arp2/3 complex mediates the formation of branched actin networks in the cytoplasm, providing the force for cell motility. In addition to its role in the cytoplasmic cytoskeleton, the Arp2/3 complex also promotes actin polymerization in the nucleus, thereby regulating gene transcription and repair of damaged DNA. The Arp2/3 complex promotes homologous recombination (HR) repair in response to DNA [...]	https://smart.embl.de/smart/DDvec.cgi?smart=154:Pfam_P16-Arc(10|154)+

ARPC5L	9606.ENSP00000345361	Actin-related protein 2/3 complex subunit 5-like protein; May function as component of the Arp2/3 complex which is involved in regulation of actin polymerization and together with an activating nucleation-promoting factor (NPF) mediates the formation of branched actin networks; Belongs to the ARPC5 family.	https://smart.embl.de/smart/DDvec.cgi?smart=153:Pfam_P16-Arc(9|153)+
WAS	9606.ENSP00000365891	Wiskott-Aldrich syndrome protein; Effector protein for Rho-type GTPases that regulates actin filament reorganization via its interaction with the Arp2/3 complex. Important for efficient actin polymerization. Possible regulator of lymphocyte and platelet function. Mediates actin filament reorganization and the formation of actin pedestals upon infection by pathogenic bacteria. In addition to its role in the cytoplasmic cytoskeleton, also promotes actin polymerization in the nucleus, thereby regulating gene transcription and repair of damaged DNA. Promotes homologous recombination (HR) r [...]	https://smart.embl.de/smart/DDvec.cgi?smart=502:WH1(39|145)+PBD(238|274)+WH2(430|447)+
WASL	9606.ENSP00000223023	Neural Wiskott-Aldrich syndrome protein; Regulates actin polymerization by stimulating the actin- nucleating activity of the Arp2/3 complex. Involved in various processes, such as mitosis and cytokinesis, via its role in the regulation of actin polymerization. Together with CDC42, involved in the extension and maintenance of the formation of thin, actin-rich surface projections called filopodia. In addition to its role in the cytoplasm, also plays a role in the nucleus by regulating gene transcription, probably by promoting nuclear actin polymerization. Binds to HSF1/HSTF1 and forms a [...]	https://smart.embl.de/smart/DDvec.cgi?smart=505:WH1(34|138)+PBD(203|239)+WH2(405|422)+WH2(433|450)+

Supplementary Table 17. Member list of the DBDS Genomic Consortium

First name	Surname	Email	Affiliation 1	Affiliation 2
Karina	Banasik	karina.banasik@cpr.ku.dk	Novo Nordisk Foundation Center for Protein Research, Faculty of Health and Medical Sciences, University of Copenhagen, Copenhagen, Denmark	Department of Gynecology and Obstetrics, Copenhagen University Hospital Hvidovre, Copenhagen, Denmark
Jakob	Bay	jabay@regionsjaelland.dk	Department of Clinical Immunology, Zealand University Hospital, Køge, Denmark	
Jens Kjærgaard	Boldsen	jenbol@rm.dk	Department of Clinical Immunology, Aarhus University Hospital, Aarhus, Denmark	
Thorsten	Brodersen	thobr@regionsjaelland.dk	Department of Clinical Immunology, Zealand University Hospital, Køge, Denmark	
Søren	Brunak	soren.brunak@cpr.ku.dk	Novo Nordisk Foundation Center for Protein Research, Faculty of Health and Medical Sciences, University of Copenhagen, Copenhagen, Denmark	
Kristoffer	Burgdorf	kristoffer.soelvsten.burgdorf@regionh.dk	Novo Nordisk Foundation Center for Protein Research, Faculty of Health and Medical Sciences, University of Copenhagen, Copenhagen, Denmark	
Mona Ameri	Chalmer	mona.ameri.chalmer@regionh.dk	Danish Headache Center, Department of Neurology, Copenhagen University Hospital, Rigshospitalet-Glostrup, Copenhagen, Denmark	
Maria	Didriksen	maria.didriksen@regionh.dk	Department of Clinical Immunology, Copenhagen University Hospital, Rigshospitalet, Copenhagen, Denmark	
Khoa Manh	Dinh	khoadinh@rm.dk	Department of Clinical Immunology, Aarhus University Hospital, Aarhus, Denmark	
Joseph	Dowsett	joseph.dowsett@regionh.dk	Department of Clinical Immunology, Copenhagen University Hospital, Rigshospitalet, Copenhagen, Denmark	

Christian	Erikstrup	christian.erikstrup@skejby.rm.dk	Department of Clinical Immunology, Aarhus University Hospital, Aarhus, Denmark	Department of Clinical Medicine, Health, Aarhus University, Aarhus, Denmark
Bjarke	Feenstra	Bjarke Feenstra <FEE@ssi.dk>	Department of Clinical Immunology, Copenhagen University Hospital, Rigshospitalet, Copenhagen, Denmark	Department of Epidemiology Research, Statens Serum Institut, Copenhagen, Denmark
Frank	Geller	FGE@ssi.dk	Department of Clinical Immunology, Copenhagen University Hospital, Rigshospitalet, Copenhagen, Denmark	Department of Epidemiology Research, Statens Serum Institut, Copenhagen, Denmark
Daniel	Gudbjartsson	Daniel.Gudbjartsson@decode.is	deCODE Genetics, Reykjavik, Iceland	
Thomas Folkmann	Hansen	thomas.hansen@regionh.dk	Danish Headache Center, Department of Neurology, Copenhagen University Hospital, Rigshospitalet-Glostrup, Copenhagen, Denmark	
Lotte	Hindhede	LOTHIN@rm.dk	Department of Clinical Immunology, Aarhus University Hospital, Aarhus, Denmark	
Henrik	Hjalgrim	HHJ@cancer.dk	Danish Cancer Society Research Center, Copenhagen, Denmark	Department of Epidemiology Research, Statens Serum Institut, Copenhagen, Denmark
Rikke Louise	Jacobsen	rikke.louise.jacobsen@regionh.dk	Department of Clinical Immunology, Copenhagen University Hospital, Rigshospitalet, Copenhagen, Denmark	
Gregor	Jemec	gbj@regionsjaelland.dk	Department of Dermatology, Zealand University hospital, Roskilde, Denmark	
Bitten Aagaard	Jensen	biaaj@rn.dk	Department of Clinical Immunology, Aalborg University Hospital, Aalborg, Denmark	
Katrine	Kaspersen	kathkasp@rm.dk	Department of Clinical Immunology, Aarhus University Hospital, Aarhus, Denmark	
Bertram Dalskov	Kjerulff	berkje@rm.dk	Department of Clinical Immunology, Aarhus University Hospital, Aarhus, Denmark	
Lisette	Kogelman	Lisette.kogelman@regionh.dk	Danish Headache Center, Department of Neurology, Copenhagen University Hospital, Rigshospitalet-Glostrup, Copenhagen, Denmark	

Margit Anita Hørup	Larsen	Margit.Anita.Hoerup.Larsen@regionh.dk	Department of Clinical Immunology, Copenhagen University Hospital, Rigshospitalet, Copenhagen, Denmark	
Ioannis	Louloudis	ioannis.louloudis@cpr.ku.dk	Novo Nordisk Foundation Center for Protein Research, Faculty of Health and Medical Sciences, University of Copenhagen, Copenhagen, Denmark	
Agnete	Lundgaard	agnete.lundgaard@cpr.ku.dk	Novo Nordisk Foundation Center for Protein Research, Faculty of Health and Medical Sciences, University of Copenhagen, Copenhagen, Denmark	
Susan	Mikkelsen	susanmke@rm.dk	Department of Clinical Immunology, Aarhus University Hospital, Aarhus, Denmark	
Christina	Mikkelsen	christina.mikkelsen@regionh.dk	Department of Clinical Immunology, Copenhagen University Hospital, Rigshospitalet, Copenhagen, Denmark	
Ioanna	Nissen	ioanna.nissen@regionh.dk	Department of Clinical Immunology, Copenhagen University Hospital, Rigshospitalet, Copenhagen, Denmark	
Mette	Nyegaard	nyegaard@hst.aau.dk	Department of Health Science and Technology, Faculty of Medicine, Aalborg Univeristy, Aalborg, Denmark	
Sisse Rye	Ostrowski	sisse.rye.ostrowski@regionh.dk	Department of Clinical Immunology, Copenhagen University Hospital, Rigshospitalet, Copenhagen, Denmark	Department of Clinical Medicine, Faculty of Health and Medical Sciences, University of Copenhagen, Copenhagen, Denmark
Ole Birger	Pedersen	olbp@regionsjaelland.dk	Department of Clinical Immunology, Zealand University Hospital, Køge, Denmark	Department of Clinical Medicine, Faculty of Health and Medical Sciences, University of Copenhagen, Copenhagen, Denmark
Alexander	Pil Henriksen	alexander.henriksen@cpr.ku.dk	Novo Nordisk Foundation Center for Protein Research, Faculty of Health and Medical Sciences, University of Copenhagen, Copenhagen, Denmark	

Palle Duun	Rohde	palledr@hst.aau.dk	Department of Health Science and Technology, Faculty of Medicine, Aalborg Univeristy, Aalborg, Denmark	
Klaus	Rostgaard	klar@cancer.dk	Danish Cancer Society Research Center, Copenhagen, Denmark	Department of Epidemiology Research, Statens Serum Institut, Copenhagen, Denmark
Michael	Schwinn	michael.schwinn@regionh.dk	Department of Clinical Immunology, Copenhagen University Hospital, Rigshospitalet, Copenhagen, Denmark	
Kari	Stefansson	kari.stefansson@decode.is	deCODE Genetics, Reykjavik, Iceland	
Hreinn	Stefánsson	hreinn.stefansson@decode.is	deCODE Genetics, Reykjavik, Iceland	
Erik	Sørensen	Erik.Soerensen@regionh.dk	Department of Clinical Immunology, Copenhagen University Hospital, Rigshospitalet, Copenhagen, Denmark	
Unnur	Þorsteinsdóttir	Unnur.Thorsteinsdottir@decode.is	deCODE Genetics, Reykjavik, Iceland	
Lise Wegner	Thørner	Lise.Wegner.Thoerner@regionh.dk	Department of Clinical Immunology, Copenhagen University Hospital, Rigshospitalet, Copenhagen, Denmark	
Mie	Topholm Bruun	mie.topholm.bruun@rsyd.dk	Department of Clinical Immunology, Odense University Hospital, Odense, Denmark	
Henrik	Ullum	HEUL@ssi.dk	Statens Serum Institut, Copenhagen, Denmark	
Thomas	Werge	thomas.werge@regionh.dk	Institute of Biological Psychiatry, Mental Health Centre, Sct. Hans, Copenhagen University Hospital, Roskilde, Denmark	Department of Clinical Medicine, Faculty of Health and Medical Sciences, University of Copenhagen, Copenhagen, Denmark
David	Westergaard	david.westergaard@cpr.ku.dk	Novo Nordisk Foundation Center for Protein Research, Faculty of Health and Medical Sciences, University of Copenhagen, Copenhagen, Denmark	
